# Supplementary figures and images for: Distinctive genes and signaling pathways associated with type 2 diabetes-related periodontitis: Preliminary study
Source: PLoS One. 2024 Jan 19;19(1):e0296925. doi: 10.1371/journal.pone.0296925 (PMC10798476; doi:10.1371/journal.pone.0296925)

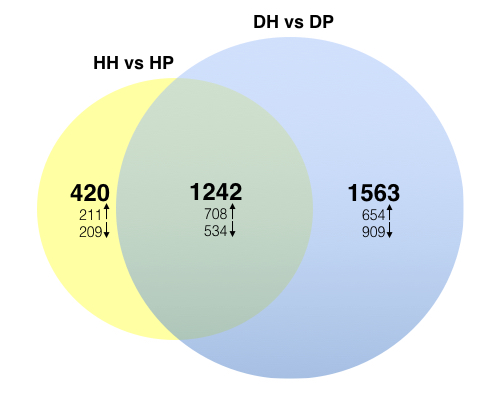

Supplement: S1 Fig — HH: non-diabetic patients without periodontitis; HP: non-diabetic patients with periodontitis; DH: T2DM patients without periodontitis; DP: T2DM patients with periodontitis. (JPEG) [file pone.0296925.s001.jpeg]
