# Supplementary material for: Distinctive genes and signaling pathways associated with type 2 diabetes-related periodontitis: Preliminary study
Source: PLoS One. 2024 Jan 19;19(1):e0296925. doi: 10.1371/journal.pone.0296925 (PMC10798476; doi:10.1371/journal.pone.0296925)
Supplement: S1 Table — (DOCX) [file pone.0296925.s002.docx]

Table S1 - Demographic characteristics, glycemic and periodontal parameters (mean ± SD) of the study population.

| Parameters | Groups | | | |
| --- | --- | --- | --- | --- |
|  | HH  (n=9) | HP  (n=10) | DH  (n=9) | DP  (n=9) |
| Sex (M, n) | 5 | 5 | 4 | 5 |
| Age (years) | 61.0 ± 5.4 | 58.6 ± 7.1 | 59.8 ± 4.3 | 59.4 ± 7.6 |
| DM duration (years) | - | - | 7.8 ± 3.0 | 7.0 ± 2.2 |
| HbA1c (%) | 5.1 ± 0.1^a^ | 5.2 ± 0.1 ^a^ | 7.9 ± 0.2 ^b^ | 8.1 ± 0.5 ^b^ |
| Sites with plaque (FM %) | 18.2 ± 1.1 ^a^ | 65.8 ± 19.9 ^b^ | 16.9 ± 3.4 ^a^ | 70.6 ± 23.1 ^b^ |
| Sites with BoP (FM %) | 8.3 ± 0.9 ^a^ | 43.7 ± 15.1 ^b^ | 7.8 ± 0.3 ^a^ | 38.7 ± 16.2 ^b^ |
| % of sites with PD ≤ 3mm | 99.1 ± 1.0 ^a^ | 50.6 ± 11.7 ^b^ | 98.5 ± 1.1^a^ | 48.7 ± 17.7 ^b^ |
| % of sites with PD (4-6 mm) | 0.9 ± 0.9 ^a^ | 31.8 ± 10.9 ^b^ | 1.4 ± 0.8 ^a^ | 36.5 ± 16.8 ^b^ |
| % of sites with PD (≥ 7 mm) | 0 | 17.4 ± 12.1 | 0 | 14.7 ± 9.1 |
| % of sites with no CA loss | 98.3 ± 1.1 ^a^ | 7.3 ± 5.1 ^b^ | 97.2 ± 1.0 ^a^ | 6.3 ± 3.1 ^b^ |
| % of sites with CA loss 1-2 mm | 1.2 ± 0.9 ^a^ | 21.3 ± 9.9 ^b^ | 1.8 ± 0.7 ^a^ | 17.3 ± 10.2 ^b^ |
| % of sites with CA loss 3-4 mm | 0.5 ± 0.3 ^a^ | 45.6 ± 23.4 ^b^ | 0.9 ± 0.6 ^a^ | 46.5 ± 19.1 ^b^ |
| % of sites with CA loss ≥ 5 mm | 0 | 25.7 ± 12.3 ^b^ | 0 | 29.9 ± 8.6^b^ |
| FM PD (mm) | 2.0 ± 0.2 ^a^ | 3.8 ± 0.5 ^b^ | 2.1 ± 0.3 ^a^ | 3.8 ± 0.5 ^b^ |
| FM CAL (mm) | 2.2 ± 0.2 ^a^ | 5.1 ± 0.9 ^b^ | 2.0 ± 0.3 ^a^ | 4.8 ± 1.1 ^b^ |
| Sampled teeth PD (mm) | 2.2 ± 0.2 ^a^ | 6.2 ± 0.9^b^ | 2.1 ± 0.1 ^a^ | 6.4 ± 1.2 ^b^ |
| Sampled teeth CAL (mm) | 2.3 ± 0.1 ^a^ | 8.2 ± 1.8 ^b^ | 2.0 ± 0.2 ^a^ | 8.3 ± 2.1 ^b^ |

No difference between groups in terms of gender (Chi-square test, p>0.05)

No difference between DH and DP in terms of duration of DM (t-test, p>0.05)

a, b: different letters indicate differences between groups by One-way ANOVA and Tukey test (p<0.05).

PD: probing depth; CA: clinical attachment; CAL: clinical attachment level; BoP: bleeding on probing; HbA1c: glycated hemoglobin; SD: standard deviation; DM: diabetes mellitus; FM: full-mouth

HH: non-diabetic subjects without periodontitis; HP: non-diabetic subjects with periodontitis; DH: T2DM subjects without periodontitis; DP: T2DM subjects with periodontitis.
