# Supplementary material for: Distinctive genes and signaling pathways associated with type 2 diabetes-related periodontitis: Preliminary study
Source: PLoS One. 2024 Jan 19;19(1):e0296925. doi: 10.1371/journal.pone.0296925 (PMC10798476; doi:10.1371/journal.pone.0296925)
Supplement: S5 Table — (DOCX) [file pone.0296925.s006.docx]

| **Table 5S . Ingenuity canonical pathways related to DEGs expressed exclusively in non-diabetic patients and the respective genes that function within each of these signaling pathways** | | | | |
| --- | --- | --- | --- | --- |
| Ingenuity Canonical Pathways | -log(p-value) | Ratio | z-score | Genes |
| B Cell Receptor Signaling | 3.50E+00 | 3.84E-02 | -1 | BLNK,CARD10,EGR1,IGHA1,IGKV1D-17,IGKV2D-30,IGKV3D-11,IGKV5-2,IGKV6D-21,IGLV1-36,IGLV10-54,IGLV3-9,JUN,RASD2 |
| HIF1Œ± Signaling | 3.46E+00 | 4.90E-02 | -0.632 | EDN1,HSP90AA1,HSPA1A/HSPA1B,HSPA6,JUN,MMP13,MMP25,MMP28,RASD2,SERPINE1 |
| IGF-1 Signaling | 3.38E+00 | 6.73E-02 | NaN | CCN1,CCN3,FOS,IGFBP3,IGFBP6,JUN,RASD2 |
| Osteoarthritis Pathway | 3.06E+00 | 4.35E-02 | -0.707 | BMP2,CXCR2,DKK1,FZD8,ITGAL,MMP13,PTCH1,S100A9,SDC4,SPHK1 |
| Pulmonary Fibrosis Idiopathic Signaling Pathway | 2.97E+00 | 3.73E-02 | -0.905 | EDN1,EGR1,FOS,FZD8,JUN,MMP13,MMP25,MMP28,RASD2,SERPINE1,TERC,WNT10B |
| Salvage Pathways of Pyrimidine Ribonucleotides | 2.80E+00 | 6.25E-02 | 0 | AK9,APOBEC3A,APOBEC3F,CMPK2,MAK,UPP1 |
| Tumor Microenvironment Pathway | 2.64E+00 | 4.49E-02 | -1.414 | CSPG4,FGF12,FOS,JUN,MMP13,MMP25,MMP28,RASD2 |
| Systemic Lupus Erythematosus In B Cell Signaling Pathway | 2.63E+00 | 3.11E-02 | 0 | BLNK,FOS,IFIT3,IGHA1,IGKV1D-17,IGKV2D-30,IGKV3D-11,IGKV5-2,IGKV6D-21,IGLV1-36,IGLV10-54,IGLV3-9,JUN,RASD2 |
| IL-15 Signaling | 2.60E+00 | 3.77E-02 | NaN | IGHA1,IGKV1D-17,IGKV2D-30,IGKV3D-11,IGKV5-2,IGKV6D-21,IGLV1-36,IGLV10-54,IGLV3-9,RASD2 |
| B Cell Receptor Signaling | 2.35E+00 | 1.15E-01 | NaN | CCL20,FOS,JUN |
| Axonal Guidance Signaling | 2.30E+00 | 2.86E-02 | NaN | BMP2,EFNA4,FZD8,ITGAL,MMP13,MMP25,MMP28,PTCH1,RASD2,SEMA3C,SEMA4D,TUBB3,WAS,WNT10B |
| Salvage Pathways of Pyrimidine Deoxyribonucleotides | 2.25E+00 | 2.22E-01 | NaN | APOBEC3A,UPP1 |
| Unfolded protein response | 2.22E+00 | 5.62E-02 | NaN | DNAJA4,DNAJB1,HSPA1A/HSPA1B,HSPA6,HSPH1 |
| Role of PKR in Interferon Induction and Antiviral Response | 2.12E+00 | 4.55E-02 | 0 | ATF3,FOS,HSP90AA1,HSPA1A/HSPA1B,HSPA6,JUN |
| PCP (Planar Cell Polarity) Pathway | 2.12E+00 | 6.67E-02 | -1 | FZD8,JUN,JUNB,WNT10B |
| Role of Osteoblasts, Osteoclasts and Chondrocytes in Rheumatoid Arthritis | 2.10E+00 | 3.65E-02 | NaN | BMP2,DKK1,DKK4,FOS,FZD8,JUN,MMP13,WNT10B |
| IL-2 Signaling | 2.09E+00 | 6.56E-02 | NaN | FOS,IL2RA,JUN,RASD2 |
| TGF-Œ≤ Signaling | 2.08E+00 | 5.21E-02 | NaN | BMP2,FOS,JUN,RASD2,SERPINE1 |
| Pyrimidine Ribonucleotides Interconversion | 2.06E+00 | 9.09E-02 | NaN | AK9,CMPK2,ENTPD2 |
| PI3K Signaling in B Lymphocytes | 2.05E+00 | 4.38E-02 | -1.342 | ATF3,BLNK,CARD10,FOS,JUN,RASD2 |
| DNA Methylation and Transcriptional Repression Signaling | 2.02E+00 | 8.82E-02 | NaN | H4C2,H4C3,H4C6 |
| Pyrimidine Ribonucleotides De Novo Biosynthesis | 1.98E+00 | 8.57E-02 | NaN | AK9,CMPK2,ENTPD2 |
| NER (Nucleotide Excision Repair, Enhanced Pathway) | 1.97E+00 | 4.90E-02 | 0.447 | CHAF1B,H4C2,H4C3,H4C6,POLE4 |
| Interferon Signaling | 1.95E+00 | 8.33E-02 | NaN | IFI6,IFIT1,IFIT3 |
| Basal Cell Carcinoma Signaling | 1.89E+00 | 5.71E-02 | NaN | BMP2,FZD8,PTCH1,WNT10B |
| Inhibition of Matrix Metalloproteases | 1.89E+00 | 7.89E-02 | NaN | MMP13,MMP25,MMP28 |
| Role of IL-17A in Psoriasis | 1.86E+00 | 1.43E-01 | NaN | CCL20,S100A9 |
| Bladder Cancer Signaling | 1.78E+00 | 4.39E-02 | NaN | FGF12,MMP13,MMP25,MMP28,RASD2 |
| Coronavirus Replication Pathway | 1.77E+00 | 7.14E-02 | NaN | BAG3,IFITM10,TUBB3 |
| Aldosterone Signaling in Epithelial Cells | 1.74E+00 | 3.75E-02 | NaN | DNAJB1,DUSP1,HSP90AA1,HSPA1A/HSPA1B,HSPA6,HSPH1 |
| Granzyme A Signaling | 1.70E+00 | 1.18E-01 | NaN | H1-1,H1-5 |
| NRF2-mediated Oxidative Stress Response | 1.68E+00 | 3.30E-02 | NaN | DNAJA4,DNAJB1,FOS,HSP90AA1,JUN,JUNB,RASD2 |
| Colorectal Cancer Metastasis Signaling | 1.65E+00 | 3.03E-02 | -1.134 | FOS,FZD8,JUN,MMP13,MMP25,MMP28,RASD2,WNT10B |
| WNT/Œ≤-catenin Signaling | 1.63E+00 | 3.53E-02 | 0 | DKK1,DKK4,FZD8,GJA1,JUN,WNT10B |
| Glucocorticoid Receptor Signaling | 1.61E+00 | 2.41E-02 | NaN | CXCR2,DUSP1,FKBP4,FOS,GJA1,HSP90AA1,HSPA1A/HSPA1B,HSPA6,IL2RA,JUN,MMP13,RASD2,SERPINE1 |
| BMP signaling pathway | 1.61E+00 | 4.71E-02 | NaN | BMP2,JUN,RASD2,SOSTDC1 |
| PDGF Signaling | 1.59E+00 | 4.65E-02 | NaN | FOS,JUN,RASD2,SPHK1 |
| L-dopachrome Biosynthesis | 1.59E+00 | 5.00E-01 | NaN | TYRP1 |
| Cysteine Biosynthesis/Homocysteine Degradation | 1.59E+00 | 5.00E-01 | NaN | CBS/CBSL |
| GDP-L-fucose Biosynthesis I (from GDP-D-mannose) | 1.59E+00 | 5.00E-01 | NaN | GFUS |
| Granulocyte Adhesion and Diapedesis | 1.58E+00 | 3.45E-02 | NaN | CCL20,CXCR2,MMP13,MMP25,MMP28,SDC4 |
| UVC-Induced MAPK Signaling | 1.55E+00 | 5.88E-02 | NaN | FOS,JUN,RASD2 |
| IL-17 Signaling | 1.53E+00 | 3.35E-02 | -1.633 | CCL20,FOS,HSP90AA1,JUN,MMP13,RASD2 |
| Ceramide Signaling | 1.53E+00 | 4.44E-02 | NaN | FOS,JUN,RASD2,SPHK1 |
| Transcriptional Regulatory Network in Embryonic Stem Cells | 1.48E+00 | 5.56E-02 | NaN | H4C2,H4C3,H4C6 |
| G-Protein Coupled Receptor Signaling | 1.46E+00 | 2.18E-02 | -1.291 | ADGRE1,ADGRG7,ADORA2B,ADRA2C,CARD10,CXCR2,DUSP1,FOS,FZD8,GPR1,GPR15,JUN,KCNH2,LPAR3,RASD2 |
| Pyrimidine Deoxyribonucleotides De Novo Biosynthesis I | 1.45E+00 | 8.70E-02 | NaN | AK9,CMPK2 |
| Apelin Cardiac Fibroblast Signaling Pathway | 1.45E+00 | 8.70E-02 | NaN | SERPINE1,SPHK1 |
| Thyronamine and Iodothyronamine Metabolism | 1.42E+00 | 3.33E-01 | NaN | DIO2 |
| Thyroid Hormone Metabolism I (via Deiodination) | 1.42E+00 | 3.33E-01 | NaN | DIO2 |
| L-serine Degradation | 1.42E+00 | 3.33E-01 | NaN | SDS |
| Heparan Sulfate Biosynthesis (Late Stages) | 1.39E+00 | 5.08E-02 | NaN | AADAC,PLA1A,SULT1E1 |
| Hepatic Fibrosis Signaling Pathway | 1.37E+00 | 2.43E-02 | -0.707 | EDN1,FOS,FZD8,ITGAL,JUN,MMP13,PTCH1,RASD2,SERPINE1,WNT10B |
| Pulmonary Healing Signaling Pathway | 1.36E+00 | 3.05E-02 | 0 | FZD8,MMP13,MMP25,MMP28,RASD2,WNT10B |
| Agranulocyte Adhesion and Diapedesis | 1.36E+00 | 3.05E-02 | NaN | CCL20,CXCR2,MMP13,MMP25,MMP28,SDC4 |
| Apelin Liver Signaling Pathway | 1.35E+00 | 7.69E-02 | NaN | EDN1,EDN2 |
| SPINK1 General Cancer Pathway | 1.35E+00 | 4.92E-02 | NaN | MT1E,MT1M,RASD2 |
| PPAR Signaling | 1.33E+00 | 3.85E-02 | NaN | FOS,HSP90AA1,JUN,RASD2 |
| Thrombopoietin Signaling | 1.32E+00 | 4.76E-02 | NaN | FOS,JUN,RASD2 |
| Eumelanin Biosynthesis | 1.29E+00 | 2.50E-01 | NaN | DCT |
| Ovarian Cancer Signaling | 1.28E+00 | 3.21E-02 | NaN | EDN1,FZD8,GJA1,RASD2,WNT10B |
| Role of Macrophages, Fibroblasts and Endothelial Cells in Rheumatoid Arthritis | 1.28E+00 | 2.55E-02 | NaN | DKK1,DKK4,FOS,FZD8,JUN,MMP13,RASD2,WNT10B |
| Phagosome Formation | 1.27E+00 | 2.10E-02 | 0 | ADGRE1,ADGRG7,ADORA2B,ADRA2C,CD209,FZD8,GPR1,GPR15,IGHA1,ITGAL,LPAR3,RASD2,SPHK1,WAS |
| Heparan Sulfate Biosynthesis | 1.27E+00 | 4.55E-02 | NaN | AADAC,PLA1A,SULT1E1 |
| Role of JAK1 and JAK3 in Œ≥c Cytokine Signaling | 1.25E+00 | 4.48E-02 | NaN | BLNK,IL2RA,RASD2 |
| CDK5 Signaling | 1.23E+00 | 3.54E-02 | NaN | EGR1,FOSB,LAMA3,RASD2 |
| TNFR2 Signaling | 1.22E+00 | 6.45E-02 | NaN | FOS,JUN |
| Molecular Mechanisms of Cancer | 1.22E+00 | 2.28E-02 | NaN | ARHGEF18,BMP2,CDK20,FOS,FZD8,ITGAL,JUN,PTCH1,RASD2,WNT10B |
| Role of Tissue Factor in Cancer | 1.20E+00 | 3.48E-02 | NaN | CCN1,EGR1,MMP13,RASD2 |
| Hypoxia Signaling in the Cardiovascular System | 1.17E+00 | 4.17E-02 | NaN | EDN1,HSP90AA1,JUN |
| Role of NANOG in Mammalian Embryonic Stem Cell Pluripotency | 1.17E+00 | 3.39E-02 | NaN | BMP2,FZD8,RASD2,WNT10B |
| Thyroid Hormone Metabolism II (via Conjugation and/or Degradation) | 1.17E+00 | 6.06E-02 | NaN | DIO2,UGT1A6 |
| Glycine Cleavage Complex | 1.12E+00 | 1.67E-01 | NaN | GLDC |
| GDNF Family Ligand-Receptor Interactions | 1.12E+00 | 3.95E-02 | NaN | FOS,JUN,RASD2 |
| Neurotrophin/TRK Signaling | 1.12E+00 | 3.95E-02 | NaN | FOS,JUN,RASD2 |
| Breast Cancer Regulation by Stathmin1 | 1.11E+00 | 2.06E-02 | -1.155 | ADGRE1,ADGRG7,ADORA2B,ADRA2C,ARHGEF18,FZD8,GPR1,GPR15,JUN,LPAR3,RASD2,TUBB3 |
| Thyroid Cancer Signaling | 1.11E+00 | 3.90E-02 | NaN | FOS,JUN,RASD2 |
| Chemokine Signaling | 1.11E+00 | 3.90E-02 | NaN | FOS,JUN,RASD2 |
| Renal Cell Carcinoma Signaling | 1.09E+00 | 3.85E-02 | NaN | FOS,JUN,RASD2 |
| Estrogen-Dependent Breast Cancer Signaling | 1.09E+00 | 3.85E-02 | NaN | FOS,JUN,RASD2 |
| 14-3-3-mediated Signaling | 1.09E+00 | 3.17E-02 | NaN | FOS,JUN,RASD2,TUBB3 |
| IL-3 Signaling | 1.08E+00 | 3.80E-02 | NaN | FOS,JUN,RASD2 |
| Ferroptosis Signaling Pathway | 1.08E+00 | 3.15E-02 | NaN | ALOX5,CBS/CBSL,H2AC21,RASD2 |
| IL-17A Signaling in Fibroblasts | 1.06E+00 | 5.26E-02 | NaN | FOS,JUN |
| BAG2 Signaling Pathway | 1.06E+00 | 3.70E-02 | NaN | HSP90AA1,HSPA1A/HSPA1B,HSPA6 |
| JAK/STAT Signaling | 1.04E+00 | 3.66E-02 | NaN | FOS,JUN,RASD2 |
| HGF Signaling | 1.04E+00 | 3.05E-02 | NaN | FOS,ITGAL,JUN,RASD2 |
| Acute Phase Response Signaling | 1.04E+00 | 2.72E-02 | NaN | FOS,JUN,RASD2,SAA2,SERPINE1 |
| LPS-stimulated MAPK Signaling | 1.01E+00 | 3.53E-02 | NaN | FOS,JUN,RASD2 |
| Regulation Of The Epithelial Mesenchymal Transition In Development Pathway | 1.01E+00 | 3.53E-02 | NaN | FZD8,PTCH1,WNT10B |
| Superoxide Radicals Degradation | 1.00E+00 | 1.25E-01 | NaN | TYRP1 |
| Leukocyte Extravasation Signaling | 1.00E+00 | 2.65E-02 | 0.447 | ITGAL,MMP13,MMP25,MMP28,WAS |
| Prolactin Signaling | 9.96E-01 | 3.49E-02 | NaN | FOS,JUN,RASD2 |
| Hepatic Fibrosis / Hepatic Stellate Cell Activation | 9.91E-01 | 2.63E-02 | NaN | EDN1,IGFBP3,KLF6,MMP13,SERPINE1 |
| April Mediated Signaling | 9.87E-01 | 4.76E-02 | NaN | FOS,JUN |
| Triacylglycerol Degradation | 9.87E-01 | 4.76E-02 | NaN | AADAC,PLA1A |
| Regulation Of The Epithelial Mesenchymal Transition By Growth Factors Pathway | 9.79E-01 | 2.60E-02 | -1 | EGR1,FGF12,FOS,JUN,RASD2 |
| Natural Killer Cell Signaling | 9.71E-01 | 2.59E-02 | 0.447 | HSPA1A/HSPA1B,HSPA6,ITGAL,RASD2,WAS |
| B Cell Activating Factor Signaling | 9.71E-01 | 4.65E-02 | NaN | FOS,JUN |
| Oncostatin M Signaling | 9.71E-01 | 4.65E-02 | NaN | MMP13,RASD2 |
| Regulation of the Epithelial-Mesenchymal Transition Pathway | 9.71E-01 | 2.59E-02 | NaN | EGR1,FGF12,FZD8,RASD2,WNT10B |
| MIF Regulation of Innate Immunity | 9.55E-01 | 4.55E-02 | NaN | FOS,JUN |
| PI3K/AKT Signaling | 9.43E-01 | 2.54E-02 | NaN | CXCR2,HSP90AA1,IL2RA,ITGAL,RASD2 |
| Actin Nucleation by ARP-WASP Complex | 9.39E-01 | 3.30E-02 | NaN | ITGAL,RASD2,WAS |
| Corticotropin Releasing Hormone Signaling | 9.21E-01 | 2.76E-02 | NaN | FOS,JUN,NR4A1,PTCH1 |
| iNOS Signaling | 9.21E-01 | 4.35E-02 | NaN | FOS,JUN |
| ERBB Signaling | 9.21E-01 | 3.23E-02 | NaN | FOS,JUN,RASD2 |
| Factors Promoting Cardiogenesis in Vertebrates | 9.14E-01 | 2.74E-02 | 0 | BMP2,DKK1,FZD8,WNT10B |
| Glycine Betaine Degradation | 9.14E-01 | 1.00E-01 | NaN | SDS |
| Sertoli Cell-Sertoli Cell Junction Signaling | 9.10E-01 | 2.48E-02 | NaN | ITGAL,JUN,RASD2,TUBB3,WAS |
| Aryl Hydrocarbon Receptor Signaling | 9.07E-01 | 2.72E-02 | NaN | DCT,FOS,HSP90AA1,JUN |
| Melanocyte Development and Pigmentation Signaling | 8.89E-01 | 3.12E-02 | NaN | DCT,RASD2,TYRP1 |
| UVA-Induced MAPK Signaling | 8.79E-01 | 3.09E-02 | NaN | FOS,JUN,RASD2 |
| eNOS Signaling | 8.66E-01 | 2.63E-02 | NaN | HSP90AA1,HSPA1A/HSPA1B,HSPA6,LPAR3 |
| Primary Immunodeficiency Signaling | 8.63E-01 | 4.00E-02 | NaN | BLNK,IGHA1 |
| TNFR1 Signaling | 8.63E-01 | 4.00E-02 | NaN | FOS,JUN |
| Protein Ubiquitination Pathway | 8.54E-01 | 2.22E-02 | NaN | DNAJB1,HSP90AA1,HSPA1A/HSPA1B,HSPA6,HSPH1,USP18 |
| ERK/MAPK Signaling | 8.42E-01 | 2.35E-02 | NaN | DUSP1,DUSP2,FOS,ITGAL,RASD2 |
| UVB-Induced MAPK Signaling | 8.36E-01 | 3.85E-02 | NaN | FOS,JUN |
| Communication between Innate and Adaptive Immune Cells | 8.30E-01 | 1.95E-02 | NaN | IGHA1,IGKV1D-17,IGKV2D-30,IGKV3D-11,IGKV5-2,IGKV6D-21,IGLV1-36,IGLV10-54,IGLV3-9 |
| HMGB1 Signaling | 8.18E-01 | 2.52E-02 | NaN | FOS,JUN,RASD2,SERPINE1 |
| Cardiac Hypertrophy Signaling (Enhanced) | 8.18E-01 | 1.89E-02 | -0.707 | ADRA2C,CXCR2,EDN1,FGF12,FZD8,IL2RA,ITGAL,JUN,RASD2,WNT10B |
| HOTAIR Regulatory Pathway | 8.18E-01 | 2.52E-02 | 0 | MMP13,MMP25,MMP28,WNT10B |
| Telomerase Signaling | 8.15E-01 | 2.88E-02 | NaN | HSP90AA1,IL2RA,RASD2 |
| CSDE1 Signaling Pathway | 7.99E-01 | 3.64E-02 | NaN | FOS,SDC4 |
| CD27 Signaling in Lymphocytes | 7.99E-01 | 3.64E-02 | NaN | FOS,JUN |
| EGF Signaling | 7.99E-01 | 3.64E-02 | NaN | FOS,JUN |
| Human Embryonic Stem Cell Pluripotency | 7.90E-01 | 2.45E-02 | NaN | BMP2,FZD8,SPHK1,WNT10B |
| Colanic Acid Building Blocks Biosynthesis | 7.77E-01 | 7.14E-02 | NaN | GFUS |
| Prostate Cancer Signaling | 7.75E-01 | 2.75E-02 | NaN | HSP90AA1,NKX3-1,RASD2 |
| CXCR4 Signaling | 7.72E-01 | 2.41E-02 | NaN | EGR1,FOS,JUN,RASD2 |
| Role of IL-17A in Arthritis | 7.72E-01 | 3.51E-02 | NaN | CCL20,MMP13 |
| Polyamine Regulation in Colon Cancer | 7.62E-01 | 3.45E-02 | NaN | FOS,JUN |
| Leukotriene Biosynthesis | 7.50E-01 | 6.67E-02 | NaN | ALOX5 |
| MSP-RON Signaling In Macrophages Pathway | 7.35E-01 | 2.63E-02 | NaN | FOS,JUN,RASD2 |
| Neuregulin Signaling | 7.26E-01 | 2.61E-02 | NaN | HSP90AA1,ITGAL,RASD2 |
| FAK Signaling | 7.19E-01 | 2.59E-02 | NaN | ITGAL,RASD2,WAS |
| Cholecystokinin/Gastrin-mediated Signaling | 7.12E-01 | 2.56E-02 | NaN | FOS,JUN,RASD2 |
| ERB2-ERBB3 Signaling | 6.97E-01 | 3.12E-02 | NaN | JUN,RASD2 |
| Renin-Angiotensin Signaling | 6.84E-01 | 2.48E-02 | NaN | FOS,JUN,RASD2 |
| Isoleucine Degradation I | 6.80E-01 | 5.56E-02 | NaN | SDS |
| CD40 Signaling | 6.76E-01 | 3.03E-02 | NaN | FOS,JUN |
| Synaptogenesis Signaling Pathway | 6.72E-01 | 1.94E-02 | -0.447 | EFNA4,NLGN1,NRXN1,RASD2,SNAP25,WAS |
| Agrin Interactions at Neuromuscular Junction | 6.58E-01 | 2.94E-02 | NaN | JUN,RASD2 |
| Ethanol Degradation IV | 6.58E-01 | 5.26E-02 | NaN | TYRP1 |
| Endothelin-1 Signaling | 6.54E-01 | 2.15E-02 | NaN | EDN1,FOS,JUN,RASD2 |
| Atherosclerosis Signaling | 6.50E-01 | 2.38E-02 | NaN | ALOX5,MMP13,TNFRSF12A |
| GNRH Signaling | 6.48E-01 | 2.14E-02 | NaN | EGR1,FOS,JUN,RASD2 |
| Inflammasome pathway | 6.38E-01 | 5.00E-02 | NaN | NLRC4 |
| IL-6 Signaling | 6.36E-01 | 2.34E-02 | NaN | FOS,JUN,RASD2 |
| Growth Hormone Signaling | 6.29E-01 | 2.82E-02 | NaN | FOS,IGFBP3 |
| P2Y Purigenic Receptor Signaling Pathway | 6.23E-01 | 2.31E-02 | NaN | FOS,JUN,RASD2 |
| IL-10 Signaling | 6.22E-01 | 2.78E-02 | NaN | FOS,JUN |
| TREM1 Signaling | 6.22E-01 | 2.78E-02 | NaN | LAT2,NLRC4 |
| ERK5 Signaling | 6.22E-01 | 2.78E-02 | NaN | FOS,RASD2 |
| Valine Degradation I | 6.20E-01 | 4.76E-02 | NaN | SDS |
| Gap Junction Signaling | 6.13E-01 | 2.06E-02 | NaN | CCN3,GJA1,RASD2,TUBB3 |
| RAR Activation | 6.07E-01 | 2.05E-02 | NaN | DUSP1,FOS,IGFBP3,JUN |
| Protein Kinase A Signaling | 6.07E-01 | 1.79E-02 | 0 | ADD2,DUSP1,DUSP2,DUSP22,H1-1,H1-5,PTCH1 |
| GŒ±12/13 Signaling | 6.06E-01 | 2.26E-02 | NaN | JUN,LPAR3,RASD2 |
| Adipogenesis pathway | 6.06E-01 | 2.26E-02 | NaN | BMP2,FZD8,WNT10B |
| Role of WNT/GSK-3Œ≤ Signaling in the Pathogenesis of Influenza | 6.04E-01 | 2.70E-02 | NaN | FZD8,WNT10B |
| Cardiomyocyte Differentiation via BMP Receptors | 6.02E-01 | 4.55E-02 | NaN | BMP2 |
| STAT3 Pathway | 5.93E-01 | 2.22E-02 | NaN | CXCR2,IL2RA,RASD2 |
| Ephrin Receptor Signaling | 5.88E-01 | 2.01E-02 | NaN | EFNA4,ITGAL,RASD2,WAS |
| Cysteine Biosynthesis III (mammalia) | 5.87E-01 | 4.35E-02 | NaN | CBS/CBSL |
| Signaling by Rho Family GTPases | 5.83E-01 | 1.89E-02 | -1 | ARHGEF18,FOS,ITGAL,JUN,WAS |
| Estrogen Receptor Signaling | 5.83E-01 | 1.75E-02 | -0.816 | FOS,HSP90AA1,JUN,MMP13,MMP25,MMP28,RASD2 |
| RAC Signaling | 5.82E-01 | 2.19E-02 | NaN | ITGAL,JUN,RASD2 |
| MSP-RON Signaling In Cancer Cells Pathway | 5.82E-01 | 2.19E-02 | NaN | FOS,JUN,RASD2 |
| VDR/RXR Activation | 5.80E-01 | 2.60E-02 | NaN | IGFBP3,IGFBP6 |
| Toll-like Receptor Signaling | 5.80E-01 | 2.60E-02 | NaN | FOS,JUN |
| Tumoricidal Function of Hepatic Natural Killer Cells | 5.70E-01 | 4.17E-02 | NaN | ITGAL |
| GŒ±i Signaling | 5.64E-01 | 2.14E-02 | NaN | ADRA2C,CXCR2,RASD2 |
| Apelin Endothelial Signaling Pathway | 5.64E-01 | 2.14E-02 | NaN | FOS,JUN,RASD2 |
| Xenobiotic Metabolism AHR Signaling Pathway | 5.64E-01 | 2.53E-02 | NaN | HSP90AA1,UGT1A6 |
| IL-8 Signaling | 5.51E-01 | 1.93E-02 | NaN | CXCR2,FOS,JUN,RASD2 |
| Huntington's Disease Signaling | 5.41E-01 | 1.81E-02 | NaN | DNAJB1,HSPA1A/HSPA1B,HSPA6,JUN,SNAP25 |
| NAD Signaling Pathway | 5.38E-01 | 2.07E-02 | NaN | GJA1,H1-1,H1-5 |
| Semaphorin Neuronal Repulsive Signaling Pathway | 5.38E-01 | 2.07E-02 | NaN | CSPG4,ITGAL,SEMA4D |
| FcŒ≥RIIB Signaling in B Lymphocytes | 5.35E-01 | 2.41E-02 | NaN | BLNK,RASD2 |
| TR/RXR Activation | 5.27E-01 | 2.38E-02 | NaN | DIO2,RAB3B |
| VEGF Family Ligand-Receptor Interactions | 5.27E-01 | 2.38E-02 | NaN | FOS,RASD2 |
| Sonic Hedgehog Signaling | 5.02E-01 | 3.45E-02 | NaN | PTCH1 |
| Regulation of Cellular Mechanics by Calpain Protease | 5.00E-01 | 2.27E-02 | NaN | ITGAL,RASD2 |
| RANK Signaling in Osteoclasts | 4.93E-01 | 2.25E-02 | NaN | FOS,JUN |
| Crosstalk between Dendritic Cells and Natural Killer Cells | 4.81E-01 | 2.20E-02 | NaN | CD209,ITGAL |
| Senescence Pathway | 4.76E-01 | 1.70E-02 | 0.447 | ATF3,JUN,RASD2,SAA2,SERPINE1 |
| Inhibition of Angiogenesis by TSP1 | 4.67E-01 | 3.12E-02 | NaN | JUN |
| cAMP-mediated signaling | 4.62E-01 | 1.75E-02 | 0 | ADORA2B,ADRA2C,CXCR2,DUSP1 |
| Airway Inflammation in Asthma | 4.56E-01 | 3.03E-02 | NaN | CCL20 |
| IL-1 Signaling | 4.51E-01 | 2.08E-02 | NaN | FOS,JUN |
| 4-1BB Signaling in T Lymphocytes | 4.46E-01 | 2.94E-02 | NaN | JUN |
| Fatty Acid Œ≤-oxidation I | 4.46E-01 | 2.94E-02 | NaN | SDS |
| CREB Signaling in Neurons | 4.40E-01 | 1.52E-02 | -1 | ADGRE1,ADGRG7,ADORA2B,ADRA2C,FZD8,GPR1,GPR15,LPAR3,RASD2 |
| Coagulation System | 4.35E-01 | 2.86E-02 | NaN | SERPINE1 |
| Wound Healing Signaling Pathway | 4.29E-01 | 1.68E-02 | 0 | FOS,JUN,LAMA3,RASD2 |
| Erythropoietin Signaling Pathway | 4.29E-01 | 1.79E-02 | NaN | FOS,JUN,RASD2 |
| Androgen Signaling | 4.29E-01 | 1.79E-02 | NaN | DNAJB1,HSP90AA1,JUN |
| Glioblastoma Multiforme Signaling | 4.29E-01 | 1.79E-02 | NaN | FZD8,RASD2,WNT10B |
| Neuropathic Pain Signaling In Dorsal Horn Neurons | 4.28E-01 | 2.00E-02 | NaN | FOS,KCNH2 |
| TWEAK Signaling | 4.26E-01 | 2.78E-02 | NaN | TNFRSF12A |
| Superpathway of Methionine Degradation | 4.26E-01 | 2.78E-02 | NaN | CBS/CBSL |
| Sumoylation Pathway | 4.24E-01 | 1.98E-02 | NaN | FOS,JUN |
| Actin Cytoskeleton Signaling | 4.23E-01 | 1.67E-02 | NaN | FGF12,ITGAL,RASD2,WAS |
| Xenobiotic Metabolism CAR Signaling Pathway | 4.13E-01 | 1.74E-02 | NaN | HSP90AA1,SULT1E1,UGT1A6 |
| Mouse Embryonic Stem Cell Pluripotency | 4.12E-01 | 1.94E-02 | NaN | FZD8,RASD2 |
| Paxillin Signaling | 3.98E-01 | 1.89E-02 | NaN | ITGAL,RASD2 |
| Xenobiotic Metabolism PXR Signaling Pathway | 3.98E-01 | 1.70E-02 | NaN | HSP90AA1,SULT1E1,UGT1A6 |
| Tight Junction Signaling | 3.95E-01 | 1.69E-02 | NaN | FOS,JUN,SNAP25 |
| Role of MAPK Signaling in Promoting the Pathogenesis of Influenza | 3.83E-01 | 1.83E-02 | NaN | JUN,RASD2 |
| Retinol Biosynthesis | 3.82E-01 | 2.44E-02 | NaN | AADAC |
| Regulation of Actin-based Motility by Rho | 3.79E-01 | 1.82E-02 | NaN | ITGAL,WAS |
| Dermatan Sulfate Biosynthesis (Late Stages) | 3.75E-01 | 2.38E-02 | NaN | SULT1E1 |
| PPARŒ±/RXRŒ± Activation | 3.73E-01 | 1.64E-02 | NaN | HSP90AA1,JUN,RASD2 |
| Airway Pathology in Chronic Obstructive Pulmonary Disease | 3.65E-01 | 1.77E-02 | NaN | CCL20,FGF12 |
| Role of IL-17F in Allergic Inflammatory Airway Diseases | 3.60E-01 | 2.27E-02 | NaN | MMP13 |
| Chondroitin Sulfate Biosynthesis (Late Stages) | 3.53E-01 | 2.22E-02 | NaN | SULT1E1 |
| PAK Signaling | 3.48E-01 | 1.71E-02 | NaN | ITGAL,RASD2 |
| PFKFB4 Signaling Pathway | 3.45E-01 | 2.17E-02 | NaN | AMPD1 |
| ILK Signaling | 3.39E-01 | 1.55E-02 | NaN | BMP2,FOS,JUN |
| Ephrin A Signaling | 3.38E-01 | 2.13E-02 | NaN | EFNA4 |
| Adrenomedullin signaling pathway | 3.36E-01 | 1.55E-02 | NaN | FOS,KCNH2,RASD2 |
| Coronavirus Pathogenesis Pathway | 3.33E-01 | 1.54E-02 | NaN | FOS,JUN,SERPINE1 |
| Xenobiotic Metabolism Signaling | 3.31E-01 | 1.48E-02 | NaN | HSP90AA1,RASD2,SULT1E1,UGT1A6 |
| RHOA Signaling | 3.27E-01 | 1.64E-02 | NaN | LPAR3,RTKN |
| GŒ±s Signaling | 3.27E-01 | 1.64E-02 | NaN | ADD2,ADORA2B |
| Opioid Signaling Pathway | 3.20E-01 | 1.45E-02 | NaN | FOS,FOSB,RASD2,RGS1 |
| Melanoma Signaling | 3.19E-01 | 2.00E-02 | NaN | RASD2 |
| Nicotine Degradation III | 3.19E-01 | 2.00E-02 | NaN | UGT1A6 |
| Gustation Pathway | 3.18E-01 | 1.50E-02 | NaN | ENTPD2,KCNH2,TAS2R14 |
| Endocannabinoid Developing Neuron Pathway | 3.16E-01 | 1.60E-02 | NaN | RASD2,STMN2 |
| Integrin Signaling | 3.03E-01 | 1.46E-02 | NaN | ITGAL,RASD2,WAS |
| Chondroitin Sulfate Biosynthesis | 3.01E-01 | 1.89E-02 | NaN | SULT1E1 |
| fMLP Signaling in Neutrophils | 2.98E-01 | 1.54E-02 | NaN | RASD2,WAS |
| Sirtuin Signaling Pathway | 2.98E-01 | 1.41E-02 | NaN | H1-1,H1-5,JUN,TIMM8B |
| Melatonin Degradation I | 2.96E-01 | 1.85E-02 | NaN | UGT1A6 |
| Dermatan Sulfate Biosynthesis | 2.91E-01 | 1.82E-02 | NaN | SULT1E1 |
| Nicotine Degradation II | 2.91E-01 | 1.82E-02 | NaN | UGT1A6 |
| Th2 Pathway | 2.87E-01 | 1.50E-02 | NaN | IL2RA,JUN |
| CNTF Signaling | 2.85E-01 | 1.79E-02 | NaN | RASD2 |
| Cell Cycle Control of Chromosomal Replication | 2.85E-01 | 1.79E-02 | NaN | CDK20 |
| IL-12 Signaling and Production in Macrophages | 2.84E-01 | 1.49E-02 | NaN | FOS,JUN |
| Neurovascular Coupling Signaling Pathway | 2.81E-01 | 1.41E-02 | NaN | ADORA2B,ENTPD2,GJA1 |
| Serotonin Degradation | 2.80E-01 | 1.75E-02 | NaN | UGT1A6 |
| Cancer Drug Resistance By Drug Efflux | 2.75E-01 | 1.72E-02 | NaN | RASD2 |
| Xenobiotic Metabolism General Signaling Pathway | 2.71E-01 | 1.45E-02 | NaN | RASD2,UGT1A6 |
| Semaphorin Signaling in Neurons | 2.70E-01 | 1.69E-02 | NaN | SEMA4D |
| Superpathway of Melatonin Degradation | 2.70E-01 | 1.69E-02 | NaN | UGT1A6 |
| Endometrial Cancer Signaling | 2.65E-01 | 1.67E-02 | NaN | RASD2 |
| HER-2 Signaling in Breast Cancer | 2.56E-01 | 1.35E-02 | NaN | FOS,JUN,RASD2 |
| Phospholipases | 2.56E-01 | 1.61E-02 | NaN | PLA1A |
| Activation of IRF by Cytosolic Pattern Recognition Receptors | 2.51E-01 | 1.59E-02 | NaN | JUN |
| Mitotic Roles of Polo-Like Kinase | 2.51E-01 | 1.59E-02 | NaN | HSP90AA1 |
| WNT/Ca+ pathway | 2.51E-01 | 1.59E-02 | NaN | FZD8 |
| Pyridoxal 5'-phosphate Salvage Pathway | 2.43E-01 | 1.54E-02 | NaN | MAK |
| Eicosanoid Signaling | 2.43E-01 | 1.54E-02 | NaN | ALOX5 |
| ERBB4 Signaling | 2.38E-01 | 1.52E-02 | NaN | RASD2 |
| Remodeling of Epithelial Adherens Junctions | 2.38E-01 | 1.52E-02 | NaN | TUBB3 |
| Phagosome Maturation | 2.38E-01 | 1.34E-02 | NaN | SNAP25,TUBB3 |
| PTEN Signaling | 2.36E-01 | 1.33E-02 | NaN | ITGAL,RASD2 |
| IL-17A Signaling in Airway Cells | 2.34E-01 | 1.49E-02 | NaN | CCL20 |
| Relaxin Signaling | 2.33E-01 | 1.32E-02 | NaN | FOS,JUN |
| GM-CSF Signaling | 2.23E-01 | 1.43E-02 | NaN | RASD2 |
| Glioma Invasiveness Signaling | 2.18E-01 | 1.41E-02 | NaN | RASD2 |
| IL-7 Signaling Pathway | 2.11E-01 | 1.37E-02 | NaN | JUN |
| Antiproliferative Role of Somatostatin Receptor 2 | 2.08E-01 | 1.35E-02 | NaN | RASD2 |
| Role of MAPK Signaling in Inhibiting the Pathogenesis of Influenza | 2.08E-01 | 1.35E-02 | NaN | JUN |
| Caveolar-mediated Endocytosis Signaling | 2.04E-01 | 1.33E-02 | NaN | ITGAL |
| Angiopoietin Signaling | 2.01E-01 | 1.32E-02 | NaN | RASD2 |
| Macropinocytosis Signaling | 2.01E-01 | 1.32E-02 | NaN | RASD2 |
| Maturity Onset Diabetes of Young (MODY) Signaling | 1.98E-01 | 1.30E-02 | NaN | APOL6 |
| Dilated Cardiomyopathy Signaling Pathway | 0.00E+00 | 6.76E-03 | NaN | BAG3 |
| Oxytocin Signaling Pathway | 0.00E+00 | 1.10E-02 | NaN | FOS,GJA1,RASD2 |
| Pyroptosis Signaling Pathway | 0.00E+00 | 1.11E-02 | NaN | NLRC4 |
| Circadian Rhythm Signaling | 0.00E+00 | 3.83E-03 | NaN | RASD2 |
| Synaptic Long Term Potentiation | 0.00E+00 | 7.94E-03 | NaN | RASD2 |
| Fc Epsilon RI Signaling | 0.00E+00 | 8.47E-03 | NaN | RASD2 |
| Synaptic Long Term Depression | 0.00E+00 | 5.32E-03 | NaN | RASD2 |
| LPS/IL-1 Mediated Inhibition of RXR Function | 0.00E+00 | 9.01E-03 | NaN | JUN,SULT1E1 |
| p53 Signaling | 0.00E+00 | 1.02E-02 | NaN | JUN |
| Hepatic Cholestasis | 0.00E+00 | 5.41E-03 | NaN | JUN |
| LXR/RXR Activation | 0.00E+00 | 8.13E-03 | NaN | SAA2 |
| FXR/RXR Activation | 0.00E+00 | 8.00E-03 | NaN | SAA2 |
| Œ±-Adrenergic Signaling | 0.00E+00 | 9.43E-03 | NaN | RASD2 |
| Clathrin-mediated Endocytosis Signaling | 0.00E+00 | 5.21E-03 | NaN | FGF12 |
| FcŒ≥ Receptor-mediated Phagocytosis in Macrophages and Monocytes | 0.00E+00 | 1.08E-02 | NaN | WAS |
| Role of Pattern Recognition Receptors in Recognition of Bacteria and Viruses | 0.00E+00 | 6.85E-03 | NaN | NLRC4 |
| Role of NFAT in Regulation of the Immune Response | 0.00E+00 | 1.05E-02 | NaN | BLNK,FOS,JUN,RASD2 |
| NF-Œ∫B Activation by Viruses | 0.00E+00 | 1.28E-02 | NaN | RASD2 |
| CCR5 Signaling in Macrophages | 0.00E+00 | 6.73E-03 | NaN | FOS,JUN |
| Calcium-induced T Lymphocyte Apoptosis | 0.00E+00 | 3.94E-03 | NaN | NR4A1 |
| T Helper Cell Differentiation | 0.00E+00 | 3.75E-03 | NaN | IL2RA |
| CCR3 Signaling in Eosinophils | 0.00E+00 | 7.63E-03 | NaN | RASD2 |
| CD28 Signaling in T Helper Cells | 0.00E+00 | 9.62E-03 | NaN | FOS,JUN,WAS |
| Virus Entry via Endocytic Pathways | 0.00E+00 | 9.62E-03 | NaN | RASD2 |
| Cellular Effects of Sildenafil (Viagra) | 0.00E+00 | 6.94E-03 | NaN | KCNH2 |
| Thrombin Signaling | 0.00E+00 | 4.83E-03 | NaN | RASD2 |
| Cardiac Hypertrophy Signaling | 0.00E+00 | 1.18E-02 | NaN | ADRA2C,JUN,RASD2 |
| ICOS-ICOSL Signaling in T Helper Cells | 0.00E+00 | 3.31E-03 | NaN | IL2RA |
| FLT3 Signaling in Hematopoietic Progenitor Cells | 0.00E+00 | 1.27E-02 | NaN | RASD2 |
| ATM Signaling | 0.00E+00 | 1.04E-02 | NaN | JUN |
| Germ Cell-Sertoli Cell Junction Signaling | 0.00E+00 | 1.20E-02 | NaN | RASD2,TUBB3 |
| Glioma Signaling | 0.00E+00 | 8.20E-03 | NaN | RASD2 |
| Acute Myeloid Leukemia Signaling | 0.00E+00 | 1.11E-02 | NaN | RASD2 |
| Chronic Myeloid Leukemia Signaling | 0.00E+00 | 9.43E-03 | NaN | RASD2 |
| Non-Small Cell Lung Cancer Signaling | 0.00E+00 | 1.08E-02 | NaN | RASD2 |
| Production of Nitric Oxide and Reactive Oxygen Species in Macrophages | 0.00E+00 | 1.06E-02 | NaN | FOS,JUN |
| p70S6K Signaling | 0.00E+00 | 7.75E-03 | NaN | RASD2 |
| mTOR Signaling | 0.00E+00 | 4.88E-03 | NaN | RASD2 |
| G Beta Gamma Signaling | 0.00E+00 | 7.75E-03 | NaN | RASD2 |
| Sphingosine-1-phosphate Signaling | 0.00E+00 | 8.55E-03 | NaN | SPHK1 |
| Systemic Lupus Erythematosus Signaling | 0.00E+00 | 7.28E-03 | NaN | FOS,JUN,RASD2 |
| CDC42 Signaling | 0.00E+00 | 1.19E-02 | NaN | FOS,ITGAL,JUN,WAS |
| EIF2 Signaling | 0.00E+00 | 9.39E-03 | NaN | ATF3,RASD2 |
| AMPK Signaling | 0.00E+00 | 8.30E-03 | NaN | ADRA2C,AK9 |
| Hereditary Breast Cancer Signaling | 0.00E+00 | 7.19E-03 | NaN | RASD2 |
| Phospholipase C Signaling | 0.00E+00 | 8.58E-03 | NaN | ARHGEF18,BLNK,ITGAL,RASD2 |
| Regulation of eIF4 and p70S6K Signaling | 0.00E+00 | 1.14E-02 | NaN | ITGAL,RASD2 |
| Role of NFAT in Cardiac Hypertrophy | 0.00E+00 | 4.57E-03 | NaN | RASD2 |
| Regulation of IL-2 Expression in Activated and Anergic T Lymphocytes | 0.00E+00 | 1.14E-02 | NaN | FOS,JUN,RASD2 |
| NUR77 Signaling in T Lymphocytes | 0.00E+00 | 3.64E-03 | NaN | NR4A1 |
| PKCŒ∏ Signaling in T Lymphocytes | 0.00E+00 | 8.50E-03 | NaN | FOS,JUN,RASD2 |
| Role of MAPK Signaling in the Pathogenesis of Influenza | 0.00E+00 | 1.27E-02 | NaN | RASD2 |
| Role of Hypercytokinemia/hyperchemokinemia in the Pathogenesis of Influenza | 0.00E+00 | 1.25E-02 | NaN | IFIT3 |
| OX40 Signaling Pathway | 0.00E+00 | 4.10E-03 | NaN | JUN |
| NGF Signaling | 0.00E+00 | 8.55E-03 | NaN | RASD2 |
| RHOGDI Signaling | 0.00E+00 | 9.43E-03 | NaN | ARHGEF18,ITGAL |
| Hematopoiesis from Pluripotent Stem Cells | 0.00E+00 | 4.26E-03 | NaN | IGHA1 |
| D-myo-inositol-5-phosphate Metabolism | 0.00E+00 | 1.05E-02 | NaN | DUSP1,DUSP2 |
| D-myo-inositol (1,4,5,6)-Tetrakisphosphate Biosynthesis | 0.00E+00 | 1.15E-02 | NaN | DUSP1,DUSP2 |
| Superpathway of Inositol Phosphate Compounds | 0.00E+00 | 8.77E-03 | NaN | DUSP1,DUSP2 |
| D-myo-inositol (3,4,5,6)-tetrakisphosphate Biosynthesis | 0.00E+00 | 1.15E-02 | NaN | DUSP1,DUSP2 |
| 3-phosphoinositide Degradation | 0.00E+00 | 1.08E-02 | NaN | DUSP1,DUSP2 |
| 3-phosphoinositide Biosynthesis | 0.00E+00 | 1.00E-02 | NaN | DUSP1,DUSP2 |
| Epithelial Adherens Junction Signaling | 0.00E+00 | 1.29E-02 | NaN | RASD2,WAS |
| PEDF Signaling | 0.00E+00 | 1.19E-02 | NaN | RASD2 |
| TEC Kinase Signaling | 0.00E+00 | 8.00E-03 | NaN | FOS,ITGAL,WAS |
| SAPK/JNK Signaling | 0.00E+00 | 6.64E-03 | NaN | JUN,RASD2 |
| Nitric Oxide Signaling in the Cardiovascular System | 0.00E+00 | 8.77E-03 | NaN | HSP90AA1 |
| FGF Signaling | 0.00E+00 | 1.19E-02 | NaN | FGF12 |
| GABA Receptor Signaling | 0.00E+00 | 7.58E-03 | NaN | KCNH2 |
| IL-4 Signaling | 0.00E+00 | 1.11E-02 | NaN | RASD2 |
| Insulin Receptor Signaling | 0.00E+00 | 7.35E-03 | NaN | RASD2 |
| p38 MAPK Signaling | 0.00E+00 | 8.47E-03 | NaN | DUSP1 |
| Apoptosis Signaling | 0.00E+00 | 9.80E-03 | NaN | RASD2 |
| NF-Œ∫B Signaling | 0.00E+00 | 8.17E-03 | NaN | BMP2,CARD10,RASD2 |
| VEGF Signaling | 0.00E+00 | 1.03E-02 | NaN | RASD2 |
| T Cell Receptor Signaling | 0.00E+00 | 1.06E-02 | 0 | FOS,ITGAL,JUN,RASD2 |
| Autophagy | 0.00E+00 | 9.48E-03 | NaN | FOS,JUN |
| PD-1, PD-L1 cancer immunotherapy pathway | 0.00E+00 | 9.71E-03 | NaN | IL2RA |
| Th1 and Th2 Activation Pathway | 0.00E+00 | 1.20E-02 | NaN | IL2RA,JUN |
| GP6 Signaling Pathway | 0.00E+00 | 8.06E-03 | NaN | LAMA3 |
| Neuroinflammation Signaling Pathway | 0.00E+00 | 6.56E-03 | NaN | FOS,JUN |
| Iron homeostasis signaling pathway | 0.00E+00 | 7.52E-03 | NaN | BMP2 |
| Th17 Activation Pathway | 0.00E+00 | 7.12E-03 | NaN | CCL20,HSP90AA1 |
| Endocannabinoid Cancer Inhibition Pathway | 0.00E+00 | 6.94E-03 | NaN | ATF3 |
| T Cell Exhaustion Signaling Pathway | 0.00E+00 | 8.33E-03 | NaN | FOS,JUN,RASD2 |
| Systemic Lupus Erythematosus In T Cell Signaling Pathway | 0.00E+00 | 9.95E-03 | 1 | FOS,ITGAL,JUN,RASD2 |
| White Adipose Tissue Browning Pathway | 0.00E+00 | 7.41E-03 | NaN | DIO2 |
| BEX2 Signaling Pathway | 0.00E+00 | 1.25E-02 | NaN | JUN |
| Necroptosis Signaling Pathway | 0.00E+00 | 6.54E-03 | NaN | TIMM8B |
| Insulin Secretion Signaling Pathway | 0.00E+00 | 3.77E-03 | NaN | SNAP25 |
| Kinetochore Metaphase Signaling Pathway | 0.00E+00 | 9.35E-03 | NaN | H2AC21 |
