# Supplementary material for: Distinctive genes and signaling pathways associated with type 2 diabetes-related periodontitis: Preliminary study
Source: PLoS One. 2024 Jan 19;19(1):e0296925. doi: 10.1371/journal.pone.0296925 (PMC10798476; doi:10.1371/journal.pone.0296925)
Supplement: S6 Table — (DOCX) [file pone.0296925.s007.docx]

| **Table 6S. Ingenuity canonical pathways related to the DEGs expressed exclusively in patients with T2DM and the respective genes that function within these signaling pathways** | | | | |
| --- | --- | --- | --- | --- |
| Ingenuity Canonical Pathways | -log(p-value) | Ratio | z-score | Molecules |
| Granulocyte Adhesion and Diapedesis | 5.55E+00 | 1.61E-01 | NaN | C5AR1,CCL22,CCL3,CCR1,CCR2,CCR7,CLDN17,CLDN4,CX3CL1,CXCL12,CXCL13,CXCL5,CXCL6,CXCL8,CXCR4,HRH2,IL18RAP,IL1A,IL1RN,IL36A,ITGA4,ITGAM,MMP7,MMP9,SDC2,SELE,SELP,SELPLG |
| Phagosome Formation | 4.75E+00 | 1.03E-01 | 3.25 | ABHD3,ADGRD1,ADGRE5,ADGRF4,ADGRF5,ADGRG1,ADGRL4,AP1M2,AP1S2,APBB1IP,APLNR,AVPR1A,C3,C5AR1,CALCRL,CCR1,CCR2,CCR7,CELSR1,CLEC4E,CR1,CR2,EDNRB,F2RL1,F2RL2,FCER1G,FCGR1A,FCGR1B,FCGR2C,FZD3,GPR162,GPR180,GPR182,GPR22,GPR34,GPR4,GPR52,GPR65,HRH2,ITGA1,ITGA4,ITGAM,ITGAX,LBP,LGR4,LYN,MAPK15,MAPK7,MCHR1,MSR1,P2RY11,P2RY2,PIK3R2,PLA2G2F,PLA2G4B,PLA2G4C,PLAAT1,PLD2,PTGFR,S1PR3,S1PR4,SMO,SRC,TLR2,TLR4,TLR8,VAV3,VIPR1,WIPF1 |
| Atherosclerosis Signaling | 4.55E+00 | 1.67E-01 | NaN | ABHD3,ALOX12,APOD,CCR2,CXCL12,CXCL8,CXCR4,IL1A,IL1RN,IL36A,ITGA4,MMP9,MSR1,PLA2G2F,PLA2G4B,PLA2G4C,PLAAT1,SELE,SELP,SELPLG,SERPINA1 |
| HIPPO signaling | 4.33E+00 | 1.90E-01 | 1.667 | DLG2,DLG3,DLG5,PARD3,PATJ,PPM1J,PPP1R14B,PPP1R14C,PPP1R3C,PPP2R2B,PPP2R3A,SCRIB,STK4,TEAD3,TJP2,WWC1 |
| Cardiac Hypertrophy Signaling (Enhanced) | 4.23E+00 | 1.06E-01 | -0.59 | ACVR1B,CACNA2D3,CACNB4,CAMK2A,CXCL8,EDA,EDNRB,FGF7,FGFR3,FZD3,GDPD3,GNA11,GUCY1B1,HDAC11,HSPB1,IGF1,IKBKE,IL12RB2,IL17RC,IL17RD,IL18RAP,IL1A,IL20RA,IL20RB,IL22RA1,IL36A,IL4R,IL6R,IL9R,ITGA1,ITGA4,ITGAM,ITGAX,MAP3K9,MAPK13,MAPK7,OSM,PDE10A,PDE1A,PDE4A,PDE4B,PDE4D,PDE5A,PDE6A,PDE7B,PIK3R2,PLCD1,PLCD3,PLCH2,PLCL2,PRKACB,PRKAR1B,PTGS2,SMO,TNFSF8,WNT4 |
| Adrenomedullin signaling pathway | 3.80E+00 | 1.34E-01 | -2.2 | BCL2,C3,CALCRL,CASP3,GNA11,GPR182,GUCY1B1,GUCY2C,IL1A,IL1RN,IL36A,MAPK13,MAPK15,MAPK7,PIK3R2,PLCD1,PLCD3,PLCH2,PLCL2,PPARG,PRKACB,PRKAR1B,RAMP2,RXRA,TFAP2A,TFAP2C |
| PI3K/AKT Signaling | 3.69E+00 | 1.32E-01 | 2.111 | BCL2,CCND1,CDKN1B,FOXO1,GDF15,IKBKE,IL12RB2,IL17RC,IL17RD,IL18RAP,IL20RA,IL20RB,IL22RA1,IL4R,IL6R,IL9R,INPP5J,ITGA1,ITGA4,ITGAM,ITGAX,PIK3R2,PPM1J,PPP2R2B,PPP2R3A,PTGS2 |
| Superpathway of Cholesterol Biosynthesis | 3.68E+00 | 2.86E-01 | -2.828 | ACAT2, DHCR24, DHCR7,MVD,MVK,PMVK,SQLE,TM7SF2 |
| STAT3 Pathway | 3.64E+00 | 1.48E-01 | -1.155 | BCL2,EGFR,FGFR3,FLT1,IGF1,IL12RB2,IL17RC,IL17RD,IL18RAP,IL1A,IL20RA,IL20RB,IL22RA1,IL4R,IL6R,IL9R,MAP3K9,MAPK13,PTPN2,SRC |
| Neuroinflammation Signaling Pathway | 3.52E+00 | 1.15E-01 | 0 | ACVR1B,APH1B,BCL2,BDNF,CASP3,CCL3,CD200,CD200R1,CREB5,CX3CL1,CXCL12,CXCL8,GABRQ,GLS,HLA-DRB5,IKBKE,IL6R,IRAK3,JMJD7-PLA2G4B,MAPK13,MAPK15,MAPK7,MMP9,NOS2,PIK3R2,PLA2G2F,PLA2G4B,PLA2G4C,PTGS2,PYCARD,S100B,STAT1,TLR2,TLR4,TLR8 |
| Breast Cancer Regulation by Stathmin1 | 3.43E+00 | 9.79E-02 | 0.927 | ADGRD1,ADGRE5,ADGRF4,ADGRF5,ADGRG1,ADGRL4,APLNR,ARHGEF4,ARHGEF5,AVPR1A,C5AR1,CALCRL,CAMK2A,CCND1,CCR1,CCR2,CCR7,CDKN1B,CELSR1,CREB5,E2F2,E2F8,EDNRB,F2RL1,F2RL2,FZD3,GPR162,GPR180,GPR182,GPR22,GPR34,GPR4,GPR52,GPR65,HRH2,IGF1,LGR4,MCHR1,MMP9,P2RY11,P2RY2,PIK3R2,PPM1J,PPP1R14B,PPP1R14C,PPP1R3C,PPP2R2B,PPP2R3A,PRKACB,PRKAR1B,PTGFR,S1PR3,S1PR4,SMO,TUBB2B,TUBB6,VIPR1 |
| Agranulocyte Adhesion and Diapedesis | 3.32E+00 | 1.27E-01 | NaN | C5AR1,CCL22,CCL3,CCR1,CCR2,CCR7,CLDN17,CLDN4,CX3CL1,CXCL12,CXCL13,CXCL5,CXCL6,CXCL8,CXCR4,IL1A,IL1RN,IL36A,ITGA1,ITGA4,MMP7,MMP9,SELE,SELP,SELPLG |
| LXR/RXR Activation | 3.27E+00 | 1.46E-01 | -0.832 | ABCG4,ACACA,APOD,C3,IL18RAP,IL1A,IL1RN,IL36A,LBP,MMP9,MSR1,NOS2,PTGS2,RXRA,SCD,SERPINA1,SREBF1,TLR4 |
| Sperm Motility | 3.27E+00 | 1.19E-01 | 1 | ABHD3,ALK,DDR1,EGFR,EPHA1,EPHA2,FGFR3,FLT1,GNA11,GUCY1B1,LTK,LYN,PDE1A,PDE4A,PDE4B,PDE4D,PLA2G2F,PLA2G4B,PLA2G4C,PLAAT1,PLCD1,PLCD3,PLCH2,PLCL2,PRKACB,PRKAR1B,SLC16A10,SRC,TEK |
| Role of Macrophages, Fibroblasts and Endothelial Cells in Rheumatoid Arthritis | 2.99E+00 | 1.08E-01 | NaN | C5AR1,CAMK2A,CCND1,CREB5,CXCL12,CXCL8,F2RL1,FCGR1A,FRZB,FZD3,IKBKE,IL17RC,IL18RAP,IL1A,IL1RN,IL36A,IL6R,IRAK3,LTBR,NLK,NOS2,OSM,PIK3R2,PLCD1,PLCD3,PLCH2,PLCL2,SELE,SMO,SRC,TLR2,TLR4,TLR8,WNT4 |
| G-Protein Coupled Receptor Signaling | 2.98E+00 | 9.17E-02 | -0.126 | ADGRD1,ADGRE5,ADGRF4,ADGRF5,ADGRG1,ADGRL4,APLNR,AVPR1A,C5AR1,CALCRL,CAMK2A,CCND1,CCR1,CCR2,CCR7,CELSR1,CREB5,EDNRB,F2RL1,F2RL2,FOXO1,FZD3,GDPD3,GNA11,GPR162,GPR180,GPR182,GPR22,GPR34,GPR4,GPR52,GPR65,GUCY1B1,HRH2,IKBKE,LGR4,MAP3K9,MAPK13,MAPK7,MCHR1,P2RY11,P2RY2,PDE10A,PDE1A,PDE4A,PDE4B,PDE4D,PDE5A,PDE6A,PDE7B,PIK3R2,PREX2,PRKACB,PRKAR1B,PTGFR,RGS12,RGS18,RGS2,S1PR3,S1PR4,SMO,SRC,VIPR1 |
| Role of IL-17A in Psoriasis | 2.95E+00 | 3.57E-01 | 0.447 | CXCL5,CXCL6,CXCL8,DEFB4A/DEFB4B,IL17RC |
| Cardiac Œ≤-adrenergic Signaling | 2.90E+00 | 1.25E-01 | 3 | AKAP12,CACNA2D3,CACNB4,GDPD3,GNA11,GUCY1B1,PDE10A,PDE1A,PDE4A,PDE4B,PDE4D,PDE5A,PDE6A,PDE7B,PPM1J,PPP1R14B,PPP1R14C,PPP1R3C,PPP2R2B,PPP2R3A,PRKACB,PRKAR1B |
| tRNA Splicing | 2.83E+00 | 2.00E-01 | 2.121 | GDPD3,PDE10A,PDE1A,PDE4A,PDE4B,PDE4D,PDE5A,PDE6A,PDE7B |
| CDK5 Signaling | 2.82E+00 | 1.42E-01 | 0.258 | BDNF,CAPN1,CDK5R1,GUCY1B1,LAMA1,MAPK13,MAPK15,MAPK7,PPM1J,PPP1R14B,PPP1R14C,PPP1R3C,PPP2R2B,PPP2R3A,PRKACB,PRKAR1B |
| Glucocorticoid Receptor Signaling | 2.79E+00 | 9.44E-02 | NaN | A2M,BCL2,CCL3,CXCL8,DNALI1,EGFR,FCGR1A,GTF2A1,GTF2H2,HLA-DRB5,HSPA2,IGF1,IKBKE,IL12RB2,IL17RC,IL17RD,IL18RAP,IL1A,IL1RN,IL20RA,IL20RB,IL22RA1,IL4R,IL6R,IL9R,JMJD7-PLA2G4B,KRT13,KRT23,KRT3,KRT76,KRT78,KRT80,MAPK13,MMP9,MT-ND3,MT-ND4L,NOS2,PIK3R2,PLA2G2F,PLA2G4B,PLA2G4C,PPARG,PRKACB,PTGS2,RXRA,SELE,SRC,STAT1,TLR2,TSC22D3,VIPR1 |
| Axonal Guidance Signaling | 2.75E+00 | 9.59E-02 | NaN | ABLIM1,ABLIM2,ACE2,ADAM12,ADAM23,ADAM28,ADAMDEC1,ADAMTS12,ADAMTS6,BAIAP2,BDNF,BMP7,BMP8A,CXCL12,CXCR4,EFNA5,EFNB3,EPHA1,EPHA2,FZD3,GLI2,GNA11,IGF1,ITGA1,ITGA4,ITGAM,ITGAX,MMP7,MMP9,PIK3R2,PLCD1,PLCD3,PLCH2,PLCL2,PLXNB1,PRKACB,PRKAR1B,ROBO2,RTN4R,SDC2,SEMA3A,SMO,TUBB2B,TUBB6,UNC5B,WIPF1,WNT4 |
| Superpathway of Inositol Phosphate Compounds | 2.73E+00 | 1.14E-01 | -2.132 | ALPL,DUSP14,EPHX2,EYA4,INPP5J,IPPK,ITPKC,NUDT1,NUDT4,PIK3R2,PLCD1,PLCD3,PLCH2,PPFIA3,PPM1J,PPP1R14B,PPP1R14C,PPP1R16B,PPP1R3C,PPP2R2B,PPP2R3A,PTPN13,PTPN2,SGPP2,SSH3 |
| Osteoarthritis Pathway | 2.68E+00 | 1.13E-01 | 1.789 | ALPL,CASP3,CCN4,CREB5,CXCL8,FGFR3,FRZB,FZD3,GLI2,HES1,IL18RAP,ITGA1,ITGA4,ITGAM,ITGAX,JAG1,MMP9,NOS2,PPARD,PPARG,PTGS2,S1PR3,SMO,SPP1,TLR2,TLR4 |
| Apelin Adipocyte Signaling Pathway | 2.64E+00 | 1.51E-01 | -1.155 | APLNR,CLIC2,GNA11,GPX3,GPX7,GUCY1B1,MAPK13,MAPK15,MAPK7,MGST1,MGST2,PRKACB,PRKAR1B |
| Antioxidant Action of Vitamin C | 2.63E+00 | 1.40E-01 | 1.508 | ABHD3,GSTO2,IKBKE,MAPK13,PLA2G2F,PLA2G4B,PLA2G4C,PLAAT1,PLCD1,PLCD3,PLCH2,PLCL2,PLD2,SELENOT,SLC2A3 |
| p38 MAPK Signaling | 2.62E+00 | 1.36E-01 | -0.5 | CREB5,DDIT3,HSPB1,IL18RAP,IL1A,IL1RN,IL36A,IRAK3,JMJD7-PLA2G4B,MAP4K1,MAPK13,PLA2G2F,PLA2G4B,PLA2G4C,RPS6KA4,STAT1 |
| Endothelin-1 Signaling | 2.60E+00 | 1.18E-01 | -1.528 | ABHD3,CASP3,EDNRB,GNA11,GUCY1B1,GUCY2C,MAPK13,MAPK15,MAPK7,NOS2,PIK3R2,PLA2G2F,PLA2G4B,PLA2G4C,PLAAT1,PLCD1,PLCD3,PLCH2,PLCL2,PLD2,PTGS2,SRC |
| D-myo-inositol-5-phosphate Metabolism | 2.48E+00 | 1.16E-01 | -1.414 | ALPL,DUSP14,EPHX2,EYA4,NUDT1,NUDT4,PIP4P2,PLCD1,PLCD3,PLCH2,PPFIA3,PPM1J,PPP1R14B,PPP1R14C,PPP1R16B,PPP1R3C,PPP2R2B,PPP2R3A,PTPN13,PTPN2,SGPP2,SSH3 |
| CREB Signaling in Neurons | 2.37E+00 | 8.94E-02 | 1.26 | ADGRD1,ADGRE5,ADGRF4,ADGRF5,ADGRG1,ADGRL4,APLNR,AVPR1A,C5AR1,CACNA2D3,CACNB4,CALCRL,CAMK2A,CCR1,CCR2,CCR7,CELSR1,CREB5,EDNRB,EGFR,F2RL1,F2RL2,FGFR3,FLT1,FZD3,GNA11,GPR162,GPR180,GPR182,GPR22,GPR34,GPR4,GPR52,GPR65,GUCY1B1,HRH2,IGF1,LGR4,MCHR1,P2RY11,P2RY2,PIK3R2,PLCD1,PLCD3,PLCH2,PLCL2,PRKACB,PRKAR1B,PTGFR,S1PR3,S1PR4,SMO,VIPR1 |
| Phospholipases | 2.36E+00 | 1.61E-01 | -1 | ABHD3,PLA2G2F,PLA2G4B,PLA2G4C,PLAAT1,PLCD1,PLCD3,PLCH2,PLCL2,PLD2 |
| ERK/MAPK Signaling | 2.19E+00 | 1.08E-01 | 0.229 | CREB5,HSPB1,ITGA1,ITGA4,ITGAM,ITGAX,JMJD7-PLA2G4B,PIK3R2,PLA2G2F,PLA2G4B,PLA2G4C,PPARG,PPM1J,PPP1R14B,PPP1R14C,PPP1R3C,PPP2R2B,PPP2R3A,PRKACB,PRKAR1B,RPS6KA4,SRC,STAT1 |
| Cholesterol Biosynthesis I | 2.18E+00 | 3.08E-01 | -2 | DHCR24,DHCR7,SQLE,TM7SF2 |
| Mevalonate Pathway I | 2.18E+00 | 3.08E-01 | -2 | ACAT2,MVD,MVK,PMVK |
| Cholesterol Biosynthesis II (via 24,25-dihydrolanosterol) | 2.18E+00 | 3.08E-01 | -2 | DHCR24,DHCR7,SQLE,TM7SF2 |
| Cholesterol Biosynthesis III (via Desmosterol) | 2.18E+00 | 3.08E-01 | -2 | DHCR24,DHCR7,SQLE,TM7SF2 |
| Ceramide Biosynthesis | 2.17E+00 | 4.29E-01 | NaN | DEGS2,SPTLC3,SPTSSB |
| Sertoli Cell-Sertoli Cell Junction Signaling | 2.17E+00 | 1.09E-01 | NaN | A2M,CDH1,CGN,CLDN17,CLDN4,GUCY1B1,ITGA1,ITGA4,ITGAM,ITGAX,MAP3K9,MAPK13,NOS2,PALS2,PRKACB,PRKAR1B,RAB8B,SRC,TJP2,TUBB2B,TUBB6,YBX3 |
| Hepatic Fibrosis / Hepatic Stellate Cell Activation | 2.16E+00 | 1.11E-01 | NaN | A2M,BCL2,CCN2,CCR7,COL24A1,COL6A6,COL9A3,CXCL8,EDNRB,EGFR,FLT1,IGF1,IL18RAP,IL1A,IL4R,IL6R,LAMA1,LBP,MMP9,STAT1,TLR4 |
| cAMP-mediated signaling | 2.11E+00 | 1.05E-01 | 1.528 | AKAP12,APLNR,CAMK2A,CREB5,CREM,GDPD3,GUCY1B1,HRH2,PDE10A,PDE1A,PDE4A,PDE4B,PDE4D,PDE5A,PDE6A,PDE7B,PRKACB,PRKAR1B,RGS12,RGS18,RGS2,S1PR3,SRC,VIPR1 |
| MSP-RON Signaling Pathway | 2.06E+00 | 1.55E-01 | NaN | CCR2,ITGAM,KLK12,KLK14,KLK7,NOS2,PIK3R2,TLR2,TLR4 |
| Aryl Hydrocarbon Receptor Signaling | 2.04E+00 | 1.16E-01 | -1 | AHRR,ALDH3A1,ALDH3A2,ALDH4A1,CCND1,CDKN1B,CYP3A5,GSTO2,HSPB1,IL1A,MGST1,MGST2,NFIC,NR2F1,RXRA,SRC,TGM2 |
| Endocannabinoid Neuronal Synapse Pathway | 2.01E+00 | 1.15E-01 | -1.941 | CACNA2D3,CACNB4,DNALI1,FAAH,GNA11,GUCY1B1,MAPK13,MAPK15,MAPK7,MGLL,PLCD1,PLCD3,PLCH2,PLCL2,PRKACB,PRKAR1B,PTGS2 |
| Hepatic Cholestasis | 1.98E+00 | 1.08E-01 | NaN | CXCL8,CYP3A5,EDA,GUCY1B1,IKBKE,IL18RAP,IL1A,IL1RN,IL36A,IRAK3,LBP,OSM,PRKACB,PRKAR1B,RXRA,SLC10A1,SREBF1,TJP2,TLR4,TNFSF8 |
| Proline Degradation | 1.96E+00 | 6.67E-01 | NaN | ALDH4A1,LOC102724788/PRODH |
| 3-phosphoinositide Degradation | 1.96E+00 | 1.08E-01 | -1 | ALPL,DUSP14,EPHX2,EYA4,INPP5J,NUDT1,NUDT4,PIP4P2,PPFIA3,PPM1J,PPP1R14B,PPP1R14C,PPP1R16B,PPP1R3C,PPP2R2B,PPP2R3A,PTPN13,PTPN2,SGPP2,SSH3 |
| PTEN Signaling | 1.95E+00 | 1.13E-01 | 0.277 | BCL2,CASP3,CCND1,CDKN1B,CNKSR3,EGFR,FGFR3,FLT1,FOXO1,IKBKE,INPP5J,ITGA1,ITGA4,ITGAM,ITGAX,PIK3R2,PREX2 |
| Relaxin Signaling | 1.93E+00 | 1.13E-01 | -0.378 | GDPD3,GNA11,GUCY1B1,GUCY2C,MMP9,NOS2,PDE10A,PDE1A,PDE4A,PDE4B,PDE4D,PDE5A,PDE6A,PDE7B,PIK3R2,PRKACB,PRKAR1B |
| Synaptic Long Term Depression | 1.91E+00 | 1.06E-01 | -0.229 | ABHD3,CACNA2D3,CACNB4,GNA11,GUCY1B1,GUCY2C,IGF1,LYN,NOS2,PLA2G2F,PLA2G4B,PLA2G4C,PLAAT1,PLCD1,PLCD3,PLCH2,PLCL2,PPM1J,PPP2R2B,PPP2R3A |
| IL-10 Signaling | 1.90E+00 | 1.39E-01 | NaN | CCR1,FCGR2C,IKBKE,IL18RAP,IL1A,IL1RN,IL36A,IL4R,LBP,MAPK13 |
| Dopamine-DARPP32 Feedback in cAMP Signaling | 1.88E+00 | 1.07E-01 | -0.258 | CACNA2D3,CACNB4,CAMKK1,CREB5,CREM,GUCY1B1,KCNJ11,PLCD1,PLCD3,PLCH2,PLCL2,PPM1J,PPP1R14B,PPP1R14C,PPP1R3C,PPP2R2B,PPP2R3A,PRKACB,PRKAR1B |
| Glutathione Redox Reactions I | 1.85E+00 | 2.08E-01 | -0.447 | CLIC2,GPX3,GPX7,MGST1,MGST2 |
| Hepatic Fibrosis Signaling Pathway | 1.85E+00 | 8.98E-02 | 0.18 | ACVR1B,BCL2,CACNA2D3,CACNB4,CASP3,CCL3,CCN2,CCND1,CDKN1B,CREB5,CXCL8,FLT1,FOXO1,FTL,FZD3,GLI2,IKBKE,IL18RAP,IL1A,IL1RN,IL36A,IRAK3,ITGA1,ITGA4,ITGAM,ITGAX,MAPK13,PIK3R2,PPARG,PRKACB,PRKAR1B,RHOQ,SMO,SNAI1,SPP1,TLR4,WNT4 |
| Fatty Acid Œ±-oxidation | 1.84E+00 | 2.50E-01 | -1 | ALDH3A1,ALDH3A2,ALDH4A1,PTGS2 |
| WNT/Ca+ pathway | 1.84E+00 | 1.43E-01 | -1 | CAMK2A,CREB5,FZD3,PDE6A,PLCD1,PLCD3,PLCH2,PLCL2,SMO |
| GDP-glucose Biosynthesis | 1.83E+00 | 3.33E-01 | NaN | HK1,HK2,PGM2 |
| Role of MAPK Signaling in Inhibiting the Pathogenesis of Influenza | 1.82E+00 | 1.35E-01 | 0.632 | ABHD3,CASP3,CXCL8,MAPK13,PLA2G2F,PLA2G4B,PLA2G4C,PLAAT1,PTGS2,TLR4 |
| Cellular Effects of Sildenafil (Viagra) | 1.79E+00 | 1.11E-01 | NaN | CACNA2D3,CACNB4,GUCY1B1,GUCY2C,PDE1A,PDE4A,PDE4B,PDE4D,PDE5A,PLCD1,PLCD3,PLCH2,PLCL2,PRKACB,PRKAR1B,SLC4A11 |
| MIF Regulation of Innate Immunity | 1.76E+00 | 1.59E-01 | -0.378 | JMJD7-PLA2G4B,NOS2,PLA2G2F,PLA2G4B,PLA2G4C,PTGS2,TLR4 |
| Role of IL-17F in Allergic Inflammatory Airway Diseases | 1.76E+00 | 1.59E-01 | 1.342 | CREB5,CXCL5,CXCL6,CXCL8,IGF1,IL17RC,RPS6KA4 |
| WNT/Œ≤-catenin Signaling | 1.75E+00 | 1.06E-01 | -0.258 | ACVR1B,CCND1,CDH1,FRZB,FZD3,KREMEN1,MAP4K1,MMP7,NLK,PPARD,PPM1J,PPP2R2B,PPP2R3A,SMO,SOX17,SOX21,SRC,WNT4 |
| Eicosanoid Signaling | 1.75E+00 | 1.38E-01 | 0 | ABHD3,ALOX12,DPEP1,PLA2G2F,PLA2G4B,PLA2G4C,PLAAT1,PTGFR,PTGS2 |
| Superpathway of Geranylgeranyldiphosphate Biosynthesis I (via Mevalonate) | 1.75E+00 | 2.35E-01 | -2 | ACAT2,MVD,MVK,PMVK |
| Oxytocin Signaling Pathway | 1.73E+00 | 9.52E-02 | -1.961 | CACNA2D3,CACNB4,CAMKK1,CREB5,CXCL8,EGFR,GNA11,GUCY1B1,GUCY2C,HSPB1,JMJD7-PLA2G4B,KCNA5,KCNT2,MAPK13,MAPK15,MAPK7,PIK3R2,PLA2G2F,PLA2G4B,PLA2G4C,PPARD,PPARG,PRKACB,PRKAR1B,PTGFR,PTGS2 |
| LPS/IL-1 Mediated Inhibition of RXR Function | 1.72E+00 | 9.91E-02 | 1.89 | ACSL4,ALDH3A1,ALDH3A2,ALDH4A1,CHST1,CHST15,CYP2J2,CYP3A5,FMO2,GSTO2,IL18RAP,IL1A,IL1RN,IL36A,LBP,MGST1,MGST2,RXRA,SLC10A1,SLC27A4,SREBF1,TLR4 |
| Toll-like Receptor Signaling | 1.71E+00 | 1.30E-01 | 0.378 | IL1A,IL1RN,IL36A,IRAK3,LBP,MAPK13,TLR2,TLR4,TLR8,TOLLIP |
| Xenobiotic Metabolism CAR Signaling Pathway | 1.71E+00 | 1.05E-01 | -2.357 | ALDH3A1,ALDH3A2,ALDH4A1,CHST1,CHST15,CYP3A5,EGFR,FMO2,GRIP1,GSTO2,MGST1,MGST2,NOS2,PPM1J,PPP2R2B,PPP2R3A,RXRA,SRC |
| Glucose and Glucose-1-phosphate Degradation | 1.69E+00 | 3.00E-01 | NaN | HK1,HK2,PGM2 |
| Unfolded protein response | 1.68E+00 | 1.24E-01 | -0.707 | BCL2,CD82,DDIT3,DNAJB11,DNAJC1,DNAJC10,HSPA2,PDIA6,PPARG,SREBF1,SREBF2 |
| Dopamine Receptor Signaling | 1.67E+00 | 1.28E-01 | NaN | GUCY1B1,NCS1,PPM1J,PPP1R14B,PPP1R14C,PPP1R3C,PPP2R2B,PPP2R3A,PRKACB,PRKAR1B |
| MIF-mediated Glucocorticoid Regulation | 1.67E+00 | 1.67E-01 | 0 | JMJD7-PLA2G4B,PLA2G2F,PLA2G4B,PLA2G4C,PTGS2,TLR4 |
| Complement System | 1.67E+00 | 1.67E-01 | NaN | C3,C5AR1,CR1,CR2,ITGAM,ITGAX |
| iNOS Signaling | 1.67E+00 | 1.52E-01 | -0.816 | IKBKE,IRAK3,LBP,MAPK13,NOS2,STAT1,TLR4 |
| D-myo-inositol (1,4,5,6)-Tetrakisphosphate Biosynthesis | 1.66E+00 | 1.03E-01 | -1.069 | ALPL,DUSP14,EPHX2,EYA4,NUDT1,NUDT4,PPFIA3,PPM1J,PPP1R14B,PPP1R14C,PPP1R16B,PPP1R3C,PPP2R2B,PPP2R3A,PTPN13,PTPN2,SGPP2,SSH3 |
| D-myo-inositol (3,4,5,6)-tetrakisphosphate Biosynthesis | 1.66E+00 | 1.03E-01 | -1.069 | ALPL,DUSP14,EPHX2,EYA4,NUDT1,NUDT4,PPFIA3,PPM1J,PPP1R14B,PPP1R14C,PPP1R16B,PPP1R3C,PPP2R2B,PPP2R3A,PTPN13,PTPN2,SGPP2,SSH3 |
| Insulin Secretion Signaling Pathway | 1.64E+00 | 9.43E-02 | -0.853 | CACNA2D3,CACNB4,CAMK2A,CREB5,GNA11,GUCY1B1,KCNJ11,LYN,MAPK13,PIK3R2,PLCD1,PLCD3,PLCH2,PLCL2,PRKACB,PRKAR1B,SCNN1A,SCNN1B,SLC2A3,SRC,SRP19,SRP54,SSR1,SSR2,STAT1 |
| Role of IL-17A in Arthritis | 1.64E+00 | 1.40E-01 | NaN | CXCL5,CXCL6,CXCL8,IL17RC,MAPK13,NOS2,PIK3R2,PTGS2 |
| MSP-RON Signaling In Macrophages Pathway | 1.63E+00 | 1.14E-01 | -0.277 | CREB5,HLA-DRB5,IKBKE,ITGAM,KLK12,KLK14,KLK7,NOS2,PIK3R2,PTGS2,ST14,STAT1,TLR4 |
| Xenobiotic Metabolism PXR Signaling Pathway | 1.62E+00 | 1.02E-01 | -2.357 | ALDH3A1,ALDH3A2,ALDH4A1,CAMK2A,CHST1,CHST15,CYP3A5,GRIP1,GSTO2,MGST1,MGST2,NOS2,PPP1R14B,PPP1R14C,PPP1R3C,PRKACB,PRKAR1B,RXRA |
| Cell Cycle Regulation by BTG Family Proteins | 1.61E+00 | 1.62E-01 | NaN | CCND1,E2F2,E2F8,PPM1J,PPP2R2B,PPP2R3A |
| IL-6 Signaling | 1.58E+00 | 1.09E-01 | -0.277 | A2M,CXCL8,CYP19A1,HSPB1,IKBKE,IL18RAP,IL1A,IL1RN,IL36A,IL6R,LBP,MAPK13,PIK3R2,TNFAIP6 |
| Ethanol Degradation IV | 1.58E+00 | 2.11E-01 | -1 | ALDH3A1,ALDH3A2,ALDH4A1,GPX7 |
| Tumor Microenvironment Pathway | 1.58E+00 | 1.01E-01 | -1.886 | BCL2,CCND1,CXCL12,CXCL8,CXCR4,FGF7,FOXO1,IGF1,IL6R,MMP7,MMP9,NOS2,OSM,PIK3R2,PTGS2,SLC2A3,SPP1,TIAM1 |
| p70S6K Signaling | 1.55E+00 | 1.09E-01 | -0.535 | EGFR,F2RL1,F2RL2,IL4R,LYN,PIK3R2,PLCD1,PLCD3,PLCH2,PLCL2,PPM1J,PPP2R2B,PPP2R3A,SRC |
| Xenobiotic Metabolism Signaling | 1.54E+00 | 9.23E-02 | NaN | AHRR,ALDH3A1,ALDH3A2,ALDH4A1,CAMK2A,CHST1,CHST15,CYP3A5,FMO2,FTL,GRIP1,GSTO2,IL1A,MAF,MAP3K9,MAPK13,MAPK7,MGST1,MGST2,NOS2,PIK3R2,PPM1J,PPP2R2B,PPP2R3A,RXRA |
| Glioblastoma Multiforme Signaling | 1.52E+00 | 1.01E-01 | -1.807 | CCND1,CDKN1B,E2F2,E2F8,EGFR,FOXO1,FZD3,IGF1,PIK3R2,PLCD1,PLCD3,PLCH2,PLCL2,RHOQ,SMO,SRC,WNT4 |
| CMP-N-acetylneuraminate Biosynthesis I (Eukaryotes) | 1.47E+00 | 4.00E-01 | NaN | GNE,NANS |
| Th2 Pathway | 1.46E+00 | 1.05E-01 | 0.277 | ACVR1B,APH1B,BHLHE41,CCR1,CXCR4,GATA3,HLA-DRB5,IL12RB2,IL24,IL4R,JAG1,MAF,NOTCH3,PIK3R2 |
| UVA-Induced MAPK Signaling | 1.44E+00 | 1.13E-01 | -2.121 | CASP3,EGFR,MAPK13,PIK3R2,PLCD1,PLCD3,PLCH2,PLCL2,RPS6KA4,SMPD3,STAT1 |
| IL-12 Signaling and Production in Macrophages | 1.43E+00 | 1.04E-01 | NaN | ALOX12,APOD,IKBKE,IL12RB2,MAF,MAPK13,NOS2,PIK3R2,PPARG,RXRA,SERPINA1,STAT1,TLR2,TLR4 |
| GPCR-Mediated Integration of Enteroendocrine Signaling Exemplified by an L Cell | 1.42E+00 | 1.22E-01 | 0.707 | GNA11,GUCY1B1,PLCD1,PLCD3,PLCH2,PLCL2,PRKACB,PRKAR1B,VIPR1 |
| Apelin Cardiomyocyte Signaling Pathway | 1.41E+00 | 1.12E-01 | -2.111 | APLNR,GNA11,MAPK13,MAPK15,MAPK7,PIK3R2,PLCD1,PLCD3,PLCH2,PLCL2,SLC9A3 |
| 3-phosphoinositide Biosynthesis | 1.39E+00 | 9.50E-02 | -1.291 | ALPL,DUSP14,EPHX2,EYA4,NUDT1,NUDT4,PIK3R2,PPFIA3,PPM1J,PPP1R14B,PPP1R14C,PPP1R16B,PPP1R3C,PPP2R2B,PPP2R3A,PTPN13,PTPN2,SGPP2,SSH3 |
| Leptin Signaling in Obesity | 1.39E+00 | 1.20E-01 | NaN | FOXO1,GUCY1B1,PIK3R2,PLCD1,PLCD3,PLCH2,PLCL2,PRKACB,PRKAR1B |
| Neuroprotective Role of THOP1 in Alzheimer's Disease | 1.39E+00 | 1.08E-01 | -0.905 | FAP,GZMB,HTRA3,KLK12,KLK7,MMP9,PRKACB,PRKAR1B,PRSS16,ST14,TMPRSS2,TMPRSS4 |
| Histamine Degradation | 1.38E+00 | 2.31E-01 | NaN | ALDH3A1,ALDH3A2,ALDH4A1 |
| Protein Kinase A Signaling | 1.36E+00 | 8.42E-02 | 1.89 | AKAP12,CAMK2A,CREB5,CREM,DUSP7,GDPD3,GUCY1B1,H1-0,KDELR2,KDELR3,PDE10A,PDE1A,PDE4A,PDE4B,PDE4D,PDE5A,PDE6A,PDE7B,PLCD1,PLCD3,PLCH2,PLCL2,PPP1R14B,PPP1R14C,PPP1R3C,PRKACB,PRKAR1B,PTGS2,PTPN13,PTPN2,PTPN3,PTPRB,SMO |
| PI3K Signaling in B Lymphocytes | 1.36E+00 | 1.02E-01 | -1.155 | C3,CAMK2A,CR2,IKBKE,IL4R,LYN,PIK3R2,PLCD1,PLCD3,PLCH2,PLCL2,PLEKHA4,TLR4,VAV3 |
| Inhibition of Angiogenesis by TSP1 | 1.35E+00 | 1.56E-01 | NaN | CASP3,GUCY1B1,MAPK13,MMP9,SDC2 |
| VDR/RXR Activation | 1.33E+00 | 1.17E-01 | -1 | CDKN1B,FOXO1,HES1,PPARD,RXRA,SERPINB1,SPP1,THBD,TRPV6 |
| Thyroid Cancer Signaling | 1.33E+00 | 1.17E-01 | -0.333 | ALK,BDNF,CCND1,CDH1,CXCL8,CXCR4,FOXO1,IGF1,PIK3R2 |
| Trehalose Degradation II (Trehalase) | 1.32E+00 | 3.33E-01 | NaN | HK1,HK2 |
| Differential Regulation of Cytokine Production in Intestinal Epithelial Cells by IL-17A and IL-17F | 1.30E+00 | 1.74E-01 | 0 | CCL3,DEFB4A/DEFB4B,IL1A,LCN2 |
| Vitamin-C Transport | 1.30E+00 | 1.74E-01 | NaN | GSTO2,LRRC8C,SELENOT,SLC2A3 |
| IL-17 Signaling | 1.30E+00 | 9.50E-02 | -0.728 | CCL22,CXCL5,CXCL8,DEFB4A/DEFB4B,EDA,IL17RC,IL1A,IL36A,LCN2,MAPK13,MMP9,MUC5B,NOS2,OSM,PIK3R2,PTGS2,TNFSF8 |
| Role of Tissue Factor in Cancer | 1.29E+00 | 1.04E-01 | NaN | CASP3,CCN2,CXCL8,EGFR,F2RL1,GNA11,LYN,MAPK13,PDIA6,PIK3R2,RPS6KA4,SRC |
| IL-17A Signaling in Airway Cells | 1.28E+00 | 1.19E-01 | -0.816 | CXCL5,CXCL6,DEFB4A/DEFB4B,IKBKE,IL17RC,MAPK13,MUC5B,PIK3R2 |
| Th1 and Th2 Activation Pathway | 1.27E+00 | 9.58E-02 | NaN | ACVR1B,APH1B,BHLHE41,CCR1,CXCR4,GATA3,HLA-DRB5,IL12RB2,IL24,IL4R,IL6R,JAG1,MAF,NOTCH3,PIK3R2,STAT1 |
| Role of MAPK Signaling in the Pathogenesis of Influenza | 1.27E+00 | 1.14E-01 | NaN | ABHD3,BCL2,CASP3,MAPK13,PLA2G2F,PLA2G4B,PLA2G4C,PLAAT1,PTGS2 |
| IL-8 Signaling | 1.27E+00 | 9.18E-02 | 1.213 | BCL2,CCND1,CDH1,CR2,CXCL8,EGFR,FLT1,GNA11,IKBKE,IRAK3,ITGAM,ITGAX,MMP9,PIK3R2,PLD2,PTGS2,RHOQ,SRC,TEK |
| Coronavirus Pathogenesis Pathway | 1.25E+00 | 9.23E-02 | 1.886 | ACE2,BCL2,BST2,CASP3,CCND1,CCR2,CXCL8,DDIT3,E2F2,E2F8,HDAC11,MAPK13,OAS1,PTGS2,PYCARD,RPS27L,STAT1,TMPRSS2 |
| Role of Hypercytokinemia/hyperchemokinemia in the Pathogenesis of Influenza | 1.24E+00 | 1.13E-01 | -0.333 | CCL3,CXCL8,IL1A,IL1RN,IL36A,OAS1,PYCARD,STAT1,TLR4 |
| Ovarian Cancer Signaling | 1.24E+00 | 9.62E-02 | -0.378 | BCL2,CCND1,E2F2,EGFR,FZD3,HDAC11,MMP7,MMP9,PIK3R2,PRKACB,PRKAR1B,PTGS2,SMO,SRC,WNT4 |
| Colorectal Cancer Metastasis Signaling | 1.22E+00 | 8.71E-02 | 0.218 | CASP3,CCND1,CDH1,EGFR,FZD3,GNA11,GUCY1B1,IL6R,MMP7,MMP9,NOS2,PIK3R2,PRKACB,PRKAR1B,PTGS2,RHOQ,SMO,SRC,STAT1,TLR2,TLR4,TLR8,WNT4 |
| Superpathway of Citrulline Metabolism | 1.21E+00 | 2.00E-01 | NaN | GLS,LOC102724788/PRODH,NOS2 |
| Pulmonary Healing Signaling Pathway | 1.21E+00 | 9.14E-02 | 0.471 | CCND1,CDH1,CXCL12,CXCR4,EGFR,FGF7,FZD3,JAG1,LYN,MAPK13,MMP7,MMP9,NOTCH3,SMO,SRC,TLR2,TLR4,WNT4 |
| Threonine Degradation II | 1.21E+00 | 1.00E+00 | NaN | GCAT |
| Sorbitol Degradation I | 1.21E+00 | 1.00E+00 | NaN | SORD |
| Coagulation System | 1.21E+00 | 1.43E-01 | -1.342 | A2M,F5,SERPINA1,TFPI,THBD |
| Semaphorin Neuronal Repulsive Signaling Pathway | 1.20E+00 | 9.66E-02 | 1.155 | GUCY1B1,ITGA1,ITGA4,ITGAM,ITGAX,PDE4A,PDE4B,PDE4D,PIK3R2,PLXNB1,PRKACB,PRKAR1B,SEMA3A,VCAN |
| Role of JAK family kinases in IL-6-type Cytokine Signaling | 1.19E+00 | 1.60E-01 | NaN | IL6R,MAPK13,OSM,STAT1 |
| Superpathway of Serine and Glycine Biosynthesis I | 1.19E+00 | 2.86E-01 | NaN | SHMT1,UBAC2 |
| IL-15 Production | 1.18E+00 | 1.00E-01 | -1.155 | ALK,DDR1,EGFR,EPHA1,EPHA2,FGFR3,FLT1,LTK,LYN,SRC,STAT1,TEK |
| Adipogenesis pathway | 1.18E+00 | 9.77E-02 | NaN | AGPAT2,BMP7,DDIT3,FGFR3,FOXO1,FZD3,GTF2H2,HDAC11,KLF5,NR1D1,PPARG,SMO,SREBF1 |
| Interferon Signaling | 1.17E+00 | 1.39E-01 | -1.342 | BCL2,IFITM2,OAS1,PTPN2,STAT1 |
| Granzyme B Signaling | 1.14E+00 | 1.88E-01 | NaN | CASP3,GZMB,LMNB2 |
| Extrinsic Prothrombin Activation Pathway | 1.14E+00 | 1.88E-01 | NaN | F5,TFPI,THBD |
| IL-17A Signaling in Gastric Cells | 1.14E+00 | 1.54E-01 | -1 | CXCL8,EGFR,IL17RC,MAPK13 |
| Estrogen-mediated S-phase Entry | 1.14E+00 | 1.54E-01 | -2 | CCND1,CDKN1B,E2F2,E2F8 |
| Production of Nitric Oxide and Reactive Oxygen Species in Macrophages | 1.14E+00 | 9.04E-02 | 0.728 | APOD,IKBKE,MAP3K9,MAPK13,NOS2,PIK3R2,PPM1J,PPP1R14B,PPP1R14C,PPP1R3C,PPP2R2B,PPP2R3A,RHOQ,SERPINA1,STAT1,TLR2,TLR4 |
| White Adipose Tissue Browning Pathway | 1.13E+00 | 9.63E-02 | -1.387 | BDNF,BMP7,CACNA2D3,CACNB4,CREB5,FCER1G,FGFR3,GUCY1B1,MAPK13,PPARG,PRKACB,PRKAR1B,RXRA |
| TREM1 Signaling | 1.13E+00 | 1.11E-01 | 1.414 | CCL3,CD83,CXCL8,DEFB4A/DEFB4B,ITGAX,TLR2,TLR4,TLR8 |
| Notch Signaling | 1.12E+00 | 1.35E-01 | NaN | APH1B,DTX2,HES1,JAG1,NOTCH3 |
| IL-7 Signaling Pathway | 1.10E+00 | 1.10E-01 | -1.134 | BCL2,CCND1,CDKN1B,FOXO1,LYN,MAPK13,PIK3R2,STAT1 |
| Pancreatic Adenocarcinoma Signaling | 1.09E+00 | 9.68E-02 | -0.707 | BCL2,CCND1,CDKN1B,E2F2,E2F8,EGFR,HDAC11,MMP9,PIK3R2,PLD2,PTGS2,STAT1 |
| Docosahexaenoic Acid (DHA) Signaling | 1.09E+00 | 1.32E-01 | NaN | BCL2,BCL2A1,CASP3,FOXO1,PIK3R2 |
| IL-17A Signaling in Fibroblasts | 1.09E+00 | 1.32E-01 | NaN | CXCL5,IKBKE,IL17RC,LCN2,MAPK13 |
| Inhibition of Matrix Metalloproteases | 1.09E+00 | 1.32E-01 | -1 | A2M,ADAM12,MMP7,MMP9,SDC2 |
| Histidine Degradation III | 1.08E+00 | 2.50E-01 | NaN | HAL,MTHFD2L |
| Ketolysis | 1.08E+00 | 2.50E-01 | NaN | ACAT2,BDH1 |
| 1D-myo-inositol Hexakisphosphate Biosynthesis II (Mammalian) | 1.08E+00 | 1.76E-01 | NaN | INPP5J,IPPK,ITPKC |
| Putrescine Degradation III | 1.08E+00 | 1.76E-01 | NaN | ALDH3A1,ALDH3A2,ALDH4A1 |
| Role of Osteoblasts, Osteoclasts and Chondrocytes in Rheumatoid Arthritis | 1.08E+00 | 8.68E-02 | NaN | ALPL,BCL2,BMP7,BMP8A,FOXO1,FRZB,FZD3,GSN,IGF1,IKBKE,IL18RAP,IL1A,IL1RN,IL36A,PIK3R2,SMO,SPP1,SRC,WNT4 |
| FXR/RXR Activation | 1.07E+00 | 9.60E-02 | NaN | APOD,C3,CYP19A1,FOXO1,IL1A,IL1RN,IL36A,PPARG,RXRA,SERPINA1,SLC10A1,SREBF1 |
| Endocannabinoid Developing Neuron Pathway | 1.07E+00 | 9.60E-02 | -1.667 | CCND1,CDKN1B,CREB5,GUCY1B1,MAPK13,MAPK15,MAPK7,MGLL,PIK3R2,PRKACB,PRKAR1B,SRC |
| Circadian Rhythm Signaling | 1.07E+00 | 8.43E-02 | NaN | BDNF,BHLHE41,CACNA2D3,CACNB4,CAMK2A,CIART,CREB5,GNA11,GUCY1B1,GUCY2C,HLF,LYN,NR1D1,PER1,PER3,PLCD1,PLCD3,PLCH2,PLCL2,PRKACB,PRKAR1B,SRC |
| Amyloid Processing | 1.07E+00 | 1.20E-01 | NaN | APH1B,CAPN1,CDK5R1,MAPK13,PRKACB,PRKAR1B |
| Synaptic Long Term Potentiation | 1.05E+00 | 9.52E-02 | 0 | CAMK2A,CREB5,GNA11,PLCD1,PLCD3,PLCH2,PLCL2,PPP1R14B,PPP1R14C,PPP1R3C,PRKACB,PRKAR1B |
| Neuropathic Pain Signaling In Dorsal Horn Neurons | 1.05E+00 | 1.00E-01 | -1.897 | BDNF,CAMK2A,PIK3R2,PLCD1,PLCD3,PLCH2,PLCL2,PRKACB,PRKAR1B,SRC |
| Airway Pathology in Chronic Obstructive Pulmonary Disease | 1.05E+00 | 9.73E-02 | NaN | APOD,CXCL8,EDA,FGF7,GZMB,IL1A,IL36A,LCN2,MMP9,OSM,TNFSF8 |
| Gap Junction Signaling | 1.04E+00 | 8.76E-02 | NaN | EGFR,GJC1,GUCY1B1,GUCY2C,MAPK7,PIK3R2,PLCD1,PLCD3,PLCH2,PLCL2,PRKACB,PRKAR1B,SP3,SRC,TJP2,TUBB2B,TUBB6 |
| PXR/RXR Activation | 1.04E+00 | 1.11E-01 | NaN | ALDH3A2,CYP3A5,FOXO1,PRKACB,PRKAR1B,RXRA,SCD |
| Regulation of Cellular Mechanics by Calpain Protease | 1.04E+00 | 1.02E-01 | 0 | CAPN1,CCND1,CDKN1B,EGFR,ITGA1,ITGA4,ITGAM,ITGAX,SRC |
| Pulmonary Fibrosis Idiopathic Signaling Pathway | 1.01E+00 | 8.07E-02 | -0.392 | ACVR1B,BCL2,CCN2,CCN4,CCND1,CDH1,COL24A1,COL6A6,COL9A3,CXCL12,EGFR,FGFR3,FOXO1,FZD3,GLI2,HES1,JAG1,MAPK13,MMP7,MMP9,NOTCH3,PIK3R2,RPS6KA4,SMO,SNAI1,WNT4 |
| B Cell Receptor Signaling | 1.01E+00 | 7.95E-02 | -0.577 | APBB1IP,BCL2A1,CAMK2A,CREB5,FCGR2C,FOXO1,IGHV1-24,IGHV1-46,IGHV1-69,IGHV2-5,IGHV3-20,IGHV3-33,IGHV3-48,IGHV3-66,IGHV3-72,IGHV3-74,IGHV4-4,IGHV7-4-1,IGKV1D-16,IGKV2-24,IGKV2D-29,IGLV3-21,IKBKE,INPP5J,LYN,MAP3K9,MAPK13,PIK3R2,VAV3 |
| Oxidative Ethanol Degradation III | 1.00E+00 | 1.38E-01 | NaN | ALDH3A1,ALDH3A2,ALDH4A1,CYP2J2 |
| Sonic Hedgehog Signaling | 1.00E+00 | 1.38E-01 | -1 | GLI2,PRKACB,PRKAR1B,SMO |
| Molecular Mechanisms of Cancer | 9.96E-01 | 7.74E-02 | NaN | APH1B,ARHGEF4,ARHGEF5,BCL2,BMP7,BMP8A,CAMK2A,CASP3,CCND1,CDH1,CDK17,CDKN1B,E2F2,E2F8,FOXO1,FZD3,GNA11,GUCY1B1,HDAC11,HIPK2,ITGA1,ITGA4,ITGAM,ITGAX,MAPK13,NLK,PIK3R2,PRKACB,PRKAR1B,RASGRF2,RHOQ,SMO,SRC,WNT4 |
| Pyroptosis Signaling Pathway | 9.91E-01 | 1.00E-01 | 1 | CASP3,IL1A,MAPK13,PRKACB,PRKAR1B,PYCARD,TLR2,TLR4,TLR8 |
| Pathogenesis of Multiple Sclerosis | 9.91E-01 | 2.22E-01 | NaN | CCL3,CCR1 |
| Citrulline Biosynthesis | 9.91E-01 | 2.22E-01 | NaN | GLS,LOC102724788/PRODH |
| Salvage Pathways of Pyrimidine Deoxyribonucleotides | 9.91E-01 | 2.22E-01 | NaN | TK1,TYMP |
| Folate Transformations I | 9.91E-01 | 2.22E-01 | NaN | MTHFD2L,SHMT1 |
| Intrinsic Prothrombin Activation Pathway | 9.75E-01 | 1.22E-01 | -1 | F5,KLK12,KLK14,KLK7,THBD |
| Sphingosine-1-phosphate Signaling | 9.71E-01 | 9.40E-02 | -0.632 | CASP3,GUCY1B1,PIK3R2,PLCD1,PLCD3,PLCH2,PLCL2,RHOQ,S1PR3,S1PR4,SMPD3 |
| Tryptophan Degradation X (Mammalian, via Tryptamine) | 9.67E-01 | 1.58E-01 | NaN | ALDH3A1,ALDH3A2,ALDH4A1 |
| PPAR Signaling | 9.67E-01 | 9.62E-02 | -0.333 | IKBKE,IL18RAP,IL1A,IL1RN,IL36A,NR2F1,PPARD,PPARG,PTGS2,RXRA |
| Endocannabinoid Cancer Inhibition Pathway | 9.67E-01 | 9.03E-02 | 1.155 | CASP3,CCND1,CDH1,CDKN1B,CREB5,DDIT3,GUCY1B1,NOS2,PIK3R2,PRKACB,PRKAR1B,SMPD3,SRC |
| Corticotropin Releasing Hormone Signaling | 9.51E-01 | 8.97E-02 | -0.577 | BDNF,CACNA2D3,CACNB4,CREB5,GLI2,GUCY1B1,GUCY2C,MAPK13,NOS2,PRKACB,PRKAR1B,PTGS2,SMO |
| Xenobiotic Metabolism AHR Signaling Pathway | 9.47E-01 | 1.01E-01 | -1.414 | AHRR,ALDH3A1,ALDH3A2,ALDH4A1,GSTO2,IL1A,MGST1,MGST2 |
| Coronavirus Replication Pathway | 9.43E-01 | 1.19E-01 | -2.236 | ACE2,IFITM2,TMPRSS2,TUBB2B,TUBB6 |
| Role of Pattern Recognition Receptors in Recognition of Bacteria and Viruses | 9.36E-01 | 8.90E-02 | 1.342 | C3,C5AR1,CXCL8,EDA,IL1A,IL36A,OAS1,OSM,PIK3R2,TLR2,TLR4,TLR8,TNFSF8 |
| Factors Promoting Cardiogenesis in Vertebrates | 9.36E-01 | 8.90E-02 | -1.387 | ACVR1B,BMP7,BMP8A,CAMK2A,CCND1,CREB5,FZD3,PLCD1,PLCD3,PLCH2,PLCL2,SMO,WNT4 |
| Aldosterone Signaling in Epithelial Cells | 9.28E-01 | 8.75E-02 | -1.89 | DNAJB11,DNAJC1,DNAJC10,DNAJC25,HSPA2,HSPB1,PIK3R2,PLCD1,PLCD3,PLCH2,PLCL2,SCNN1A,SCNN1B,SMO |
| Epoxysqualene Biosynthesis | 9.24E-01 | 5.00E-01 | NaN | SQLE |
| 4-hydroxyproline Degradation I | 9.24E-01 | 5.00E-01 | NaN | ALDH4A1 |
| GDP-L-fucose Biosynthesis I (from GDP-D-mannose) | 9.24E-01 | 5.00E-01 | NaN | GMDS |
| Glycine Biosynthesis I | 9.24E-01 | 5.00E-01 | NaN | SHMT1 |
| Glutamine Degradation I | 9.24E-01 | 5.00E-01 | NaN | GLS |
| SPINK1 Pancreatic Cancer Pathway | 9.17E-01 | 1.09E-01 | 2.449 | CPA4,CPA6,F2RL1,KLK12,KLK14,KLK7 |
| Oleate Biosynthesis II (Animals) | 9.10E-01 | 2.00E-01 | NaN | FADS6,SCD |
| Small Cell Lung Cancer Signaling | 9.03E-01 | 9.57E-02 | -0.816 | BCL2,CCND1,CDKN1B,E2F2,HDAC11,IKBKE,PIK3R2,PTGS2,RXRA |
| Systemic Lupus Erythematosus In B Cell Signaling Pathway | 8.93E-01 | 7.56E-02 | -0.471 | BCL2,CCND1,CXCL8,EDA,FCGR2C,FOXO1,IGHV1-24,IGHV1-46,IGHV1-69,IGHV2-5,IGHV3-20,IGHV3-33,IGHV3-48,IGHV3-66,IGHV3-72,IGHV3-74,IGHV4-4,IGHV7-4-1,IGKV1D-16,IGKV2-24,IGKV2D-29,IGLV3-21,IL1A,IL36A,IL6R,INPP5J,LILRB3,LYN,OSM,PIK3R2,SRC,STAT1,TLR8,TNFSF8 |
| Acetone Degradation I (to Methylglyoxal) | 8.79E-01 | 1.25E-01 | NaN | CYP19A1,CYP2J2,CYP3A5,PTGR1 |
| IL-1 Signaling | 8.63E-01 | 9.38E-02 | 0.378 | GNA11,GUCY1B1,IKBKE,IL1A,IRAK3,MAPK13,PRKACB,PRKAR1B,TOLLIP |
| Melatonin Signaling | 8.57E-01 | 1.00E-01 | -0.378 | CAMK2A,PLCD1,PLCD3,PLCH2,PLCL2,PRKACB,PRKAR1B |
| Cyclins and Cell Cycle Regulation | 8.57E-01 | 9.64E-02 | -0.378 | CCND1,CDKN1B,E2F2,E2F8,HDAC11,PPM1J,PPP2R2B,PPP2R3A |
| Apelin Pancreas Signaling Pathway | 8.48E-01 | 1.11E-01 | -0.447 | APLNR,DDIT3,PIK3R2,PRKACB,PRKAR1B |
| NAD biosynthesis II (from tryptophan) | 8.42E-01 | 1.82E-01 | NaN | KMO,NMNAT3 |
| Ketogenesis | 8.42E-01 | 1.82E-01 | NaN | ACAT2,BDH1 |
| Glycogen Degradation II | 8.42E-01 | 1.82E-01 | NaN | PGM2,TYMP |
| TR/RXR Activation | 8.36E-01 | 9.52E-02 | NaN | ACACA,ME1,PFKP,PIK3R2,RXRA,SREBF1,SREBF2,UCP3 |
| Necroptosis Signaling Pathway | 8.24E-01 | 8.50E-02 | -1.387 | CAMK2A,CAPN1,JMJD7-PLA2G4B,MLKL,PLA2G2F,PLA2G4B,PLA2G4C,PPIF,PYCARD,SLC25A10,STAT1,TIMM13,TLR4 |
| PFKFB4 Signaling Pathway | 8.18E-01 | 1.09E-01 | -0.447 | CREB5,HK1,HK2,PRKACB,PRKAR1B |
| Regulation Of The Epithelial Mesenchymal Transition In Development Pathway | 8.15E-01 | 9.41E-02 | 0 | APH1B,CDH1,FZD3,GLI2,JAG1,SMO,SNAI1,WNT4 |
| Netrin Signaling | 8.12E-01 | 9.72E-02 | -1.89 | ABLIM1,ABLIM2,CACNA2D3,CACNB4,PRKACB,PRKAR1B,UNC5B |
| Epithelial Adherens Junction Signaling | 7.93E-01 | 8.39E-02 | -0.277 | ACVR1B,BAIAP2,CDH1,EGFR,NOTCH3,PPM1J,PPP2R2B,PPP2R3A,SNAI1,SRC,STK4,TIAM1,WWC1 |
| Ephrin A Signaling | 7.90E-01 | 1.06E-01 | NaN | EFNA5,EPHA1,EPHA2,PIK3R2,VAV3 |
| PCP (Planar Cell Polarity) Pathway | 7.88E-01 | 1.00E-01 | -0.447 | CELSR1,CTHRC1,FZD3,LGR4,SMO,WNT4 |
| Bladder Cancer Signaling | 7.83E-01 | 8.77E-02 | NaN | CCND1,CDH1,CXCL8,E2F2,EGFR,FGF7,FGFR3,HDAC11,MMP7,MMP9 |
| Nitric Oxide Signaling in the Cardiovascular System | 7.83E-01 | 8.77E-02 | 0 | CACNA2D3,CACNB4,FLT1,GUCY1B1,GUCY2C,PDE1A,PDE5A,PIK3R2,PRKACB,PRKAR1B |
| Apelin Cardiac Fibroblast Signaling Pathway | 7.80E-01 | 1.30E-01 | NaN | ACE2,APLNR,CCN2 |
| UDP-N-acetyl-D-galactosamine Biosynthesis II | 7.77E-01 | 1.67E-01 | NaN | HK1,HK2 |
| Ephrin Receptor Signaling | 7.75E-01 | 8.04E-02 | 1.667 | CREB5,CXCL12,CXCR4,EFNA5,EFNB3,EPHA1,EPHA2,GNA11,ITGA1,ITGA4,ITGAM,ITGAX,PTPN13,SDC2,SRC,WIPF1 |
| Gustation Pathway | 7.62E-01 | 8.00E-02 | -1.807 | CACNA2D3,CACNB4,GABRQ,GUCY1B1,KCNJ11,LIPK,LRRC8C,P2RX5,P2RY11,P2RY2,PRKACB,PRKAR1B,SCNN1A,SCNN1B,TAS2R30,TRPM4 |
| Ascorbate Recycling (Cytosolic) | 7.59E-01 | 3.33E-01 | NaN | GSTO2 |
| Anandamide Degradation | 7.59E-01 | 3.33E-01 | NaN | FAAH |
| Biotin-carboxyl Carrier Protein Assembly | 7.59E-01 | 3.33E-01 | NaN | ACACA |
| 1D-myo-inositol Hexakisphosphate Biosynthesis V (from Ins(1,3,4)P3) | 7.59E-01 | 3.33E-01 | NaN | IPPK |
| P2Y Purigenic Receptor Signaling Pathway | 7.50E-01 | 8.46E-02 | -1.265 | CREB5,GUCY1B1,P2RY11,P2RY2,PIK3R2,PLCD1,PLCD3,PLCH2,PLCL2,PRKACB,PRKAR1B |
| Caveolar-mediated Endocytosis Signaling | 7.47E-01 | 9.33E-02 | NaN | CD48,EGFR,ITGA1,ITGA4,ITGAM,ITGAX,SRC |
| IL-22 Signaling | 7.40E-01 | 1.25E-01 | NaN | IL22RA1,MAPK13,STAT1 |
| Tumoricidal Function of Hepatic Natural Killer Cells | 7.40E-01 | 1.25E-01 | NaN | CASP3,GZMB,LYVE1 |
| Bupropion Degradation | 7.40E-01 | 1.25E-01 | NaN | CYP19A1,CYP2J2,CYP3A5 |
| Dopamine Degradation | 7.40E-01 | 1.25E-01 | NaN | ALDH3A1,ALDH3A2,ALDH4A1 |
| Opioid Signaling Pathway | 7.38E-01 | 7.64E-02 | -0.943 | CACNA2D3,CACNB4,CAMK2A,CDKN1B,CLTCL1,CREB5,GNA11,GUCY1B1,LYN,MAPK15,MAPK7,PDE1A,PLD2,PRKACB,PRKAR1B,RGS11,RGS12,RGS18,RGS5,RPS6KA4,SRC |
| GPCR-Mediated Nutrient Sensing in Enteroendocrine Cells | 7.33E-01 | 8.55E-02 | -1.667 | CACNA2D3,CACNB4,GNA11,GUCY1B1,PLCD1,PLCD3,PLCH2,PLCL2,PRKACB,PRKAR1B |
| Glycogen Degradation III | 7.24E-01 | 1.54E-01 | NaN | PGM2,TYMP |
| Œ≥-glutamyl Cycle | 7.24E-01 | 1.54E-01 | NaN | CHAC1,OPLAH |
| Guanosine Nucleotides Degradation III | 7.24E-01 | 1.54E-01 | NaN | ACP3,GDA |
| Leukocyte Extravasation Signaling | 7.21E-01 | 7.94E-02 | 0.577 | CLDN17,CLDN4,CXCL12,CXCR4,DLC1,ITGA4,ITGAM,MAPK13,MMP7,MMP9,PIK3R2,SELPLG,SRC,VAV3,WIPF1 |
| Ceramide Signaling | 7.21E-01 | 8.89E-02 | -0.378 | BCL2,PIK3R2,PPM1J,PPP2R2B,PPP2R3A,S1PR3,S1PR4,SMPD3 |
| Mitotic Roles of Polo-Like Kinase | 7.19E-01 | 9.52E-02 | NaN | CAPN1,ESPL1,PKMYT1,PPM1J,PPP2R2B,PPP2R3A |
| Fc Epsilon RI Signaling | 7.17E-01 | 8.47E-02 | -0.632 | FCER1G,INPP5J,JMJD7-PLA2G4B,LYN,MAPK13,PIK3R2,PLA2G2F,PLA2G4B,PLA2G4C,VAV3 |
| MYC Mediated Apoptosis Signaling | 7.14E-01 | 1.00E-01 | 0.447 | BCL2,CASP3,IKBKE,PRKACB,PRKAR1B |
| Crosstalk between Dendritic Cells and Natural Killer Cells | 7.03E-01 | 8.79E-02 | -0.816 | CAMK2A,CCR7,CD83,FSCN2,HLA-DRB5,LTBR,MICB,TLR4 |
| Senescence Pathway | 6.97E-01 | 7.48E-02 | -0.447 | ACVR1B,CACNA2D3,CACNB4,CAPN1,CCND1,CDKN1B,CGAS,CXCL8,DHCR24,E2F2,E2F8,HIPK2,IKBKE,IL1A,MAPK15,MAPK7,PIK3R2,PPM1J,PPP2R2B,PPP2R3A,RPS6KA4,TLR2 |
| Tight Junction Signaling | 6.93E-01 | 7.91E-02 | NaN | CGN,CLDN17,CLDN4,CNKSR3,F2RL2,PATJ,PPM1J,PPP2R2B,PPP2R3A,PRKACB,PRKAR1B,TIAM1,TJP2,YBX3 |
| IL-15 Signaling | 6.90E-01 | 7.55E-02 | -1 | CXCL8,GZMB,IGHV1-24,IGHV1-46,IGHV1-69,IGHV2-5,IGHV3-20,IGHV3-33,IGHV3-48,IGHV3-66,IGHV3-72,IGHV3-74,IGHV4-4,IGHV7-4-1,IGKV1D-16,IGKV2-24,IGKV2D-29,IGLV3-21,MAPK13,PIK3R2 |
| Estrogen-Dependent Breast Cancer Signaling | 6.88E-01 | 8.97E-02 | -1.134 | CCND1,CREB5,CYP19A1,EGFR,IGF1,PIK3R2,SRC |
| Thrombin Signaling | 6.82E-01 | 7.73E-02 | -2.138 | ARHGEF4,ARHGEF5,CAMK2A,EGFR,F2RL2,GATA3,GNA11,GUCY1B1,MAPK13,PIK3R2,PLCD1,PLCD3,PLCH2,PLCL2,RHOQ,SRC |
| ILK Signaling | 6.74E-01 | 7.77E-02 | 0.277 | CASP3,CCND1,CDH1,CREB5,MMP9,NOS2,PIK3R2,PPM1J,PPP1R14B,PPP2R2B,PPP2R3A,PTGS2,RHOQ,RPS6KA4,SNAI1 |
| Regulation of the Epithelial-Mesenchymal Transition Pathway | 6.74E-01 | 7.77E-02 | NaN | APH1B,CDH1,EGFR,FGF7,FGFR3,FZD3,JAG1,MMP9,NOTCH3,PARD6G,PIK3R2,SMO,SNAI1,WNT4,ZEB1 |
| D-myo-inositol (1,4,5)-Trisphosphate Biosynthesis | 6.70E-01 | 1.15E-01 | NaN | PLCD1,PLCD3,PLCH2 |
| Kinetochore Metaphase Signaling Pathway | 6.70E-01 | 8.41E-02 | 1.633 | CENPP,CENPW,DNALI1,ESPL1,MIS12,NEK2,PPP1R14B,PPP1R14C,PPP1R3C |
| Role of Cytokines in Mediating Communication between Immune Cells | 6.68E-01 | 9.62E-02 | NaN | CXCL8,IL1A,IL1RN,IL24,IL36A |
| Wound Healing Signaling Pathway | 6.64E-01 | 7.56E-02 | 0.943 | ACVR1B,COL24A1,COL6A6,COL9A3,CXCL8,EDA,EGFR,FGF7,IL18RAP,IL1A,IL1RN,IL36A,LAMA1,MMP9,OSM,STAT1,TNFSF8,TRPA1 |
| Insulin Receptor Signaling | 6.62E-01 | 8.09E-02 | -1.508 | FOXO1,INPP5J,PIK3R2,PPP1R14B,PPP1R14C,PPP1R3C,PRKACB,PRKAR1B,RHOQ,SCNN1A,SCNN1B |
| BEX2 Signaling Pathway | 6.54E-01 | 8.75E-02 | 1.89 | BCL2,CCND1,MAP2,PPM1J,PPP2R2B,PPP2R3A,SPP1 |
| Phenylethylamine Degradation I | 6.48E-01 | 2.50E-01 | NaN | ALDH3A2 |
| Arginine Degradation I (Arginase Pathway) | 6.48E-01 | 2.50E-01 | NaN | ALDH4A1 |
| Oxidized GTP and dGTP Detoxification | 6.48E-01 | 2.50E-01 | NaN | NUDT1 |
| NAD Biosynthesis III | 6.48E-01 | 2.50E-01 | NaN | NMNAT3 |
| Phototransduction Pathway | 6.44E-01 | 9.43E-02 | NaN | GUCY1B1,GUCY2C,PDE6A,PRKACB,PRKAR1B |
| Prostate Cancer Signaling | 6.40E-01 | 8.26E-02 | NaN | BCL2,CCND1,CDKN1B,CREB5,E2F2,FOXO1,HDAC11,PIK3R2,SRD5A1 |
| Role of MAPK Signaling in Promoting the Pathogenesis of Influenza | 6.40E-01 | 8.26E-02 | -0.333 | ABHD3,BCL2,CASP3,MAPK13,PLA2G2F,PLA2G4B,PLA2G4C,PLAAT1,PTGS2 |
| Glutathione-mediated Detoxification | 6.38E-01 | 1.11E-01 | NaN | GSTO2,MGST1,MGST2 |
| Ethanol Degradation II | 6.38E-01 | 1.11E-01 | NaN | ALDH3A1,ALDH3A2,ALDH4A1 |
| Autophagy | 6.38E-01 | 7.58E-02 | 0 | ATG9B,BCL2,CDKN1B,CREB5,DDIT3,DRAM1,FOXO1,IGF1,PIK3R2,PPM1J,PPP2R2B,PPP2R3A,PRKACB,PRKAR1B,RAB7B,TLR4 |
| Cell Cycle: G1/S Checkpoint Regulation | 6.36E-01 | 8.96E-02 | 2.449 | CCND1,CDKN1B,E2F2,E2F8,FOXO1,HDAC11 |
| AMPK Signaling | 6.35E-01 | 7.47E-02 | 0.832 | ACACA,AK4,AK6,CCND1,CREB5,FOXO1,GNA11,MAPK13,PFKP,PHLPP1,PIK3R2,PPM1J,PPP2R2B,PPP2R3A,PRKACB,PRKAR1B,RAB27A,TBC1D1 |
| Fatty Acid Activation | 6.29E-01 | 1.33E-01 | NaN | ACSL4,SLC27A4 |
| Leukotriene Biosynthesis | 6.29E-01 | 1.33E-01 | NaN | DPEP1,MGST2 |
| GP6 Signaling Pathway | 6.29E-01 | 8.06E-02 | 2.333 | APBB1IP,COL24A1,COL6A6,COL9A3,FCER1G,LAMA1,LAMC3,LYN,PIK3R2,VAV3 |
| Melanocyte Development and Pigmentation Signaling | 6.20E-01 | 8.33E-02 | -0.378 | BCL2,CREB5,GUCY1B1,PIK3R2,PRKACB,PRKAR1B,RPS6KA4,SRC |
| Reelin Signaling in Neurons | 6.16E-01 | 8.00E-02 | -1 | ARHGEF4,ARHGEF5,CAMK2A,CDK5R1,DNALI1,LYN,MAP3K9,MAP4K1,PIK3R2,SRC |
| Acute Phase Response Signaling | 6.11E-01 | 7.61E-02 | 1.387 | A2M,C3,CRABP2,FTL,IKBKE,IL1A,IL1RN,IL36A,IL6R,LBP,MAPK13,OSM,PIK3R2,SERPINA1 |
| Mechanisms of Viral Exit from Host Cells | 6.11E-01 | 9.76E-02 | NaN | CHMP4C,LMNB2,SH3GL1,SH3GL3 |
| 14-3-3-mediated Signaling | 6.02E-01 | 7.94E-02 | -1.633 | CDKN1B,FOXO1,PIK3R2,PLCD1,PLCD3,PLCH2,PLCL2,SRC,TUBB2B,TUBB6 |
| Stearate Biosynthesis I (Animals) | 6.02E-01 | 9.09E-02 | 0 | ACSL4,DHCR24,ELOVL2,PTGR1,SLC27A4 |
| EGF Signaling | 6.02E-01 | 9.09E-02 | -2.236 | EGFR,MAPK13,PIK3R2,SRC,STAT1 |
| Ferroptosis Signaling Pathway | 5.90E-01 | 7.87E-02 | 1.265 | ACACA,ACSL4,ALOX12,ANGPTL4,ARF4,CHAC1,FTL,HSPB1,SREBF2,TFAP2C |
| Parkinson's Signaling | 5.87E-01 | 1.25E-01 | NaN | CASP3,MAPK13 |
| PEDF Signaling | 5.85E-01 | 8.33E-02 | -1.89 | BCL2,BDNF,IKBKE,MAPK13,PIK3R2,PPARG,ZEB1 |
| Basal Cell Carcinoma Signaling | 5.82E-01 | 8.57E-02 | 0 | BMP7,BMP8A,FZD3,GLI2,SMO,WNT4 |
| GM-CSF Signaling | 5.82E-01 | 8.57E-02 | -0.816 | BCL2A1,CAMK2A,CCND1,LYN,PIK3R2,STAT1 |
| NAD Biosynthesis from 2-amino-3-carboxymuconate Semialdehyde | 5.65E-01 | 2.00E-01 | NaN | NMNAT3 |
| Creatine-phosphate Biosynthesis | 5.65E-01 | 2.00E-01 | NaN | CKMT1A/CKMT1B |
| Tetrahydrofolate Salvage from 5,10-methenyltetrahydrofolate | 5.65E-01 | 2.00E-01 | NaN | MTHFD2L |
| Serine Biosynthesis | 5.65E-01 | 2.00E-01 | NaN | UBAC2 |
| NAD Salvage Pathway III | 5.65E-01 | 2.00E-01 | NaN | NMNAT3 |
| Citrulline-Nitric Oxide Cycle | 5.65E-01 | 2.00E-01 | NaN | NOS2 |
| dTMP De Novo Biosynthesis | 5.65E-01 | 2.00E-01 | NaN | SHMT1 |
| Folate Polyglutamylation | 5.65E-01 | 2.00E-01 | NaN | SHMT1 |
| Role of CHK Proteins in Cell Cycle Checkpoint Control | 5.62E-01 | 8.77E-02 | -0.447 | E2F2,E2F8,PPM1J,PPP2R2B,PPP2R3A |
| Neuregulin Signaling | 5.56E-01 | 7.83E-02 | -2.236 | CDK5R1,CDKN1B,EGFR,ITGA1,ITGA4,ITGAM,ITGAX,PIK3R2,SRC |
| Signaling by Rho Family GTPases | 5.53E-01 | 7.17E-02 | -1.069 | ARHGEF4,ARHGEF5,BAIAP2,CDC42EP1,CDH1,CDH6,CIT,GNA11,ITGA1,ITGA4,ITGAM,ITGAX,MAP3K9,PARD3,PIK3R2,RHOQ,SEPTIN4,SEPTIN6,WIPF1 |
| Noradrenaline and Adrenaline Degradation | 5.51E-01 | 1.00E-01 | NaN | ALDH3A1,ALDH3A2,ALDH4A1 |
| RAN Signaling | 5.50E-01 | 1.18E-01 | NaN | KPNA5,RANGAP1 |
| D-myo-inositol (1,3,4)-trisphosphate Biosynthesis | 5.50E-01 | 1.18E-01 | NaN | INPP5J,ITPKC |
| Cholecystokinin/Gastrin-mediated Signaling | 5.30E-01 | 7.69E-02 | -0.333 | CREM,EGFR,IL1A,IL1RN,IL36A,MAPK7,PTGS2,RHOQ,SRC |
| Role of PKR in Interferon Induction and Antiviral Response | 5.27E-01 | 7.58E-02 | 0.707 | CASP3,FCGR1A,HSPA2,IKBKE,IL24,MAPK13,MSR1,PYCARD,STAT1,TLR4 |
| Regulation Of The Epithelial Mesenchymal Transition By Growth Factors Pathway | 5.27E-01 | 7.29E-02 | 0.277 | CDH1,EGFR,FGF7,FGFR3,FOXO1,IKBKE,IL6R,MAPK13,MMP9,PARD6G,PIK3R2,SNAI1,TNFSF8,ZEB1 |
| HER-2 Signaling in Breast Cancer | 5.21E-01 | 7.17E-02 | -2 | ARF4,CASP3,CCND1,CDKN1B,COX4I2,EGFR,FCER1G,FOXO1,IKBKE,IL1A,LYN,PARD3,PARD6G,PIK3R2,PTGS2,SRC |
| Human Embryonic Stem Cell Pluripotency | 5.16E-01 | 7.36E-02 | NaN | BDNF,BMP7,BMP8A,FGFR3,FOXO1,FZD3,GNA11,PIK3R2,S1PR3,S1PR4,SMO,WNT4 |
| Isoleucine Degradation I | 5.14E-01 | 1.11E-01 | NaN | ACAT2,BCAT1 |
| Glycine Cleavage Complex | 4.99E-01 | 1.67E-01 | NaN | OCA2 |
| UDP-N-acetyl-D-glucosamine Biosynthesis II | 4.99E-01 | 1.67E-01 | NaN | GFPT1 |
| Tryptophan Degradation to 2-amino-3-carboxymuconate Semialdehyde | 4.99E-01 | 1.67E-01 | NaN | KMO |
| Zymosterol Biosynthesis | 4.99E-01 | 1.67E-01 | NaN | TM7SF2 |
| Actin Nucleation by ARP-WASP Complex | 4.83E-01 | 7.69E-02 | NaN | BAIAP2,ITGA1,ITGA4,ITGAM,ITGAX,RHOQ,WIPF1 |
| Œ≥-linolenate Biosynthesis II (Animals) | 4.83E-01 | 1.05E-01 | NaN | ACSL4,SLC27A4 |
| Purine Nucleotides Degradation II (Aerobic) | 4.83E-01 | 1.05E-01 | NaN | ACP3,GDA |
| Mitochondrial L-carnitine Shuttle Pathway | 4.83E-01 | 1.05E-01 | NaN | ACSL4,SLC27A4 |
| Triacylglycerol Biosynthesis | 4.83E-01 | 8.51E-02 | -1 | AGPAT2,DGAT2,ELOVL2,LPIN3 |
| Chronic Myeloid Leukemia Signaling | 4.81E-01 | 7.55E-02 | NaN | CBLC,CCND1,CDKN1B,E2F2,E2F8,HDAC11,IKBKE,PIK3R2 |
| Airway Inflammation in Asthma | 4.78E-01 | 9.09E-02 | NaN | CXCL8,MMP9,OSM |
| Retinoate Biosynthesis I | 4.78E-01 | 9.09E-02 | NaN | RDH12,RDH13,SDR16C5 |
| Neurovascular Coupling Signaling Pathway | 4.75E-01 | 7.04E-02 | 0.258 | ABHD3,CACNA2D3,CACNB4,GABRQ,GUCY1B1,GUCY2C,KCNJ11,PLA2G2F,PLA2G4B,PLA2G4C,PLAAT1,PLD2,PRKACB,PRKAR1B,PTGS2 |
| RHOA Signaling | 4.71E-01 | 7.38E-02 | -0.707 | BAIAP2,CDC42EP1,CIT,DLC1,EPHA1,IGF1,PLEKHG5,SEPTIN4,SEPTIN6 |
| GŒ±s Signaling | 4.71E-01 | 7.38E-02 | -0.378 | CREB5,GNA11,GUCY1B1,HRH2,PRKACB,PRKAR1B,RGS2,SRC,VIPR1 |
| PPARŒ±/RXRŒ± Activation | 4.70E-01 | 7.10E-02 | -0.378 | ACVR1B,GNA11,GUCY1B1,IKBKE,IL18RAP,NR2F1,PLCD1,PLCD3,PLCH2,PLCL2,PRKACB,PRKAR1B,RXRA |
| Xenobiotic Metabolism General Signaling Pathway | 4.62E-01 | 7.25E-02 | -2.53 | FTL,GSTO2,MAF,MAP3K9,MAPK13,MAPK7,MGST1,MGST2,PIK3R2,RXRA |
| Induction of Apoptosis by HIV1 | 4.58E-01 | 7.94E-02 | -0.447 | BCL2,CASP3,CXCR4,IKBKE,SLC25A10 |
| Non-Small Cell Lung Cancer Signaling | 4.57E-01 | 7.53E-02 | -2 | CCND1,E2F2,EGFR,HDAC11,PIK3R2,RXRA,STK4 |
| The Visual Cycle | 4.53E-01 | 1.00E-01 | NaN | RDH12,RDH13 |
| Inflammasome pathway | 4.53E-01 | 1.00E-01 | NaN | PYCARD,TLR4 |
| Synaptogenesis Signaling Pathway | 4.51E-01 | 6.80E-02 | -1.606 | BDNF,CACNB4,CAMK2A,CDH1,CDH6,CHN1,CREB5,EFNA5,EFNB3,EPHA1,EPHA2,GUCY1B1,LYN,PIK3R2,PRKACB,PRKAR1B,SRC,SYT11,SYT7,SYT8,TIAM1 |
| Thioredoxin Pathway | 4.45E-01 | 1.43E-01 | NaN | SELENOT |
| Inositol Pyrophosphates Biosynthesis | 4.45E-01 | 1.43E-01 | NaN | IPPK |
| Œ±-tocopherol Degradation | 4.45E-01 | 1.43E-01 | NaN | CYP4F12 |
| Purine Ribonucleosides Degradation to Ribose-1-phosphate | 4.45E-01 | 1.43E-01 | NaN | PGM2 |
| Glycoaminoglycan-protein Linkage Region Biosynthesis | 4.45E-01 | 1.43E-01 | NaN | B3GAT2 |
| FLT3 Signaling in Hematopoietic Progenitor Cells | 4.44E-01 | 7.59E-02 | -0.816 | CREB5,FLT3,MAPK13,PIK3R2,RPS6KA4,STAT1 |
| Regulation of Actin-based Motility by Rho | 4.35E-01 | 7.27E-02 | 1 | BAIAP2,GSN,ITGA1,ITGA4,ITGAM,ITGAX,RHOQ,WIPF1 |
| IL-9 Signaling | 4.35E-01 | 8.57E-02 | NaN | IL9R,PIK3R2,STAT1 |
| GNRH Signaling | 4.34E-01 | 6.95E-02 | -1.897 | CACNA2D3,CACNB4,CAMK2A,CREB5,EGFR,GNA11,GUCY1B1,MAP3K9,MAPK13,MAPK7,PRKACB,PRKAR1B,SRC |
| Role of NFAT in Cardiac Hypertrophy | 4.26E-01 | 6.85E-02 | -1.941 | CACNA2D3,CACNB4,CAMK2A,GUCY1B1,HDAC11,IGF1,MAPK13,PIK3R2,PLCD1,PLCD3,PLCH2,PLCL2,PRKACB,PRKAR1B,SRC |
| Endoplasmic Reticulum Stress Pathway | 4.26E-01 | 9.52E-02 | NaN | CASP3,DDIT3 |
| TWEAK Signaling | 4.16E-01 | 8.33E-02 | NaN | CASP3,IKBKE,TNFRSF25 |
| UVC-Induced MAPK Signaling | 4.13E-01 | 7.84E-02 | -2 | EGFR,MAPK13,SMPD3,SRC |
| HMGB1 Signaling | 4.08E-01 | 6.92E-02 | 1.134 | CXCL8,EDA,IL1A,IL36A,MAPK13,OSM,PIK3R2,RHOQ,SELE,TLR4,TNFSF8 |
| NAD Salvage Pathway II | 4.01E-01 | 9.09E-02 | NaN | ACP3,NMNAT3 |
| Superpathway of D-myo-inositol (1,4,5)-trisphosphate Metabolism | 4.01E-01 | 9.09E-02 | NaN | INPP5J,ITPKC |
| Phosphatidylethanolamine Biosynthesis II | 3.99E-01 | 1.25E-01 | NaN | ETNK1 |
| Superoxide Radicals Degradation | 3.99E-01 | 1.25E-01 | NaN | GPX7 |
| Sphingomyelin Metabolism | 3.99E-01 | 1.25E-01 | NaN | SMPD3 |
| Cardiac Hypertrophy Signaling | 3.93E-01 | 6.67E-02 | -1.069 | CACNA2D3,CACNB4,GNA11,GUCY1B1,HSPB1,IGF1,IL6R,MAP3K9,MAPK13,PIK3R2,PLCD1,PLCD3,PLCH2,PLCL2,PRKACB,PRKAR1B,RHOQ |
| VEGF Family Ligand-Receptor Interactions | 3.81E-01 | 7.14E-02 | -0.816 | FLT1,JMJD7-PLA2G4B,PIK3R2,PLA2G2F,PLA2G4B,PLA2G4C |
| FAK Signaling | 3.73E-01 | 6.90E-02 | NaN | CAPN1,EGFR,ITGA1,ITGA4,ITGAM,ITGAX,PIK3R2,SRC |
| RAR Activation | 3.71E-01 | 6.67E-02 | NaN | CRABP2,GTF2H2,GUCY1B1,MAPK13,NR2F1,PIK3R2,PRKACB,PRKAR1B,RDH12,RDH13,RXRA,SDR16C5,SRC |
| BMP signaling pathway | 3.70E-01 | 7.06E-02 | -0.447 | BMP7,BMP8A,MAPK13,PITX2,PRKACB,PRKAR1B |
| Lymphotoxin Œ≤ Receptor Signaling | 3.69E-01 | 7.41E-02 | -1 | CASP3,IKBKE,LTBR,PIK3R2 |
| Dilated Cardiomyopathy Signaling Pathway | 3.68E-01 | 6.76E-02 | -1.134 | BCL2,CACNA2D3,CACNB4,CAMK2A,GUCY1B1,MAPK13,PRKACB,PRKAR1B,RBM20,SGCD |
| Th1 Pathway | 3.63E-01 | 6.84E-02 | -0.707 | APH1B,GATA3,HLA-DRB5,IL12RB2,IL6R,NOTCH3,PIK3R2,STAT1 |
| GŒ±12/13 Signaling | 3.61E-01 | 6.77E-02 | -1.667 | CDH1,CDH6,F2RL1,F2RL2,IKBKE,MAPK7,PIK3R2,SRC,VAV3 |
| Sphingosine and Sphingosine-1-phosphate Metabolism | 3.61E-01 | 1.11E-01 | NaN | SGPP2 |
| Leucine Degradation I | 3.61E-01 | 1.11E-01 | NaN | BCAT1 |
| Dermatan Sulfate Biosynthesis | 3.55E-01 | 7.27E-02 | 2 | B3GAT2,CHST1,CHST15,DSEL |
| Nicotine Degradation II | 3.55E-01 | 7.27E-02 | -2 | CYP19A1,CYP2J2,CYP3A5,FMO2 |
| Germ Cell-Sertoli Cell Junction Signaling | 3.50E-01 | 6.63E-02 | NaN | A2M,CDH1,GSN,LAMC3,MAP3K9,PIK3R2,RAB8B,RHOQ,SRC,TUBB2B,TUBB6 |
| Growth Hormone Signaling | 3.50E-01 | 7.04E-02 | -1 | A2M,IGF1,PIK3R2,RPS6KA4,STAT1 |
| Cell Cycle Control of Chromosomal Replication | 3.41E-01 | 7.14E-02 | -1 | CDK17,CDT1,MCM4,ORC1 |
| ERK5 Signaling | 3.38E-01 | 6.94E-02 | -1.342 | CREB5,EGFR,MAPK7,RPS6KA4,SRC |
| Ephrin B Signaling | 3.38E-01 | 6.94E-02 | -0.447 | CXCL12,CXCR4,EFNB3,GNA11,VAV3 |
| CDP-diacylglycerol Biosynthesis I | 3.35E-01 | 8.00E-02 | NaN | AGPAT2,CDS1 |
| RAC Signaling | 3.27E-01 | 6.57E-02 | -1.342 | BAIAP2,CDK5R1,ITGA1,ITGA4,ITGAM,ITGAX,PARD3,PIK3R2,TIAM1 |
| RANK Signaling in Osteoclasts | 3.27E-01 | 6.74E-02 | -2.449 | GSN,IKBKE,MAP3K9,MAPK13,PIK3R2,SRC |
| Embryonic Stem Cell Differentiation into Cardiac Lineages | 3.27E-01 | 1.00E-01 | NaN | SP4 |
| Prostanoid Biosynthesis | 3.27E-01 | 1.00E-01 | NaN | PTGS2 |
| Calcium Transport I | 3.27E-01 | 1.00E-01 | NaN | ATP2C2 |
| NAD Phosphorylation and Dephosphorylation | 3.27E-01 | 1.00E-01 | NaN | ACP3 |
| Dolichyl-diphosphooligosaccharide Biosynthesis | 3.27E-01 | 1.00E-01 | NaN | ALG5 |
| Glycine Betaine Degradation | 3.27E-01 | 1.00E-01 | NaN | SHMT1 |
| MSP-RON Signaling In Cancer Cells Pathway | 3.27E-01 | 6.57E-02 | -2.333 | CCND1,CREB5,EGFR,KLK12,KLK14,KLK7,PIK3R2,SRC,ST14 |
| Glioma Signaling | 3.18E-01 | 6.56E-02 | -1 | CAMK2A,CCND1,E2F2,E2F8,EGFR,HDAC11,IGF1,PIK3R2 |
| Role of JAK1, JAK2 and TYK2 in Interferon Signaling | 3.17E-01 | 7.69E-02 | NaN | PTPN2,STAT1 |
| Paxillin Signaling | 3.17E-01 | 6.60E-02 | -1 | ITGA1,ITGA4,ITGAM,ITGAX,MAPK13,PIK3R2,SRC |
| Antiproliferative Role of Somatostatin Receptor 2 | 3.16E-01 | 6.76E-02 | NaN | CDKN1B,GUCY1B1,GUCY2C,PIK3R2,SRC |
| April Mediated Signaling | 3.15E-01 | 7.14E-02 | NaN | IKBKE,MAPK13,TNFRSF13B |
| Estrogen Biosynthesis | 3.15E-01 | 7.14E-02 | NaN | CYP19A1,CYP2J2,CYP3A5 |
| Dermatan Sulfate Biosynthesis (Late Stages) | 3.15E-01 | 7.14E-02 | NaN | CHST1,CHST15,DSEL |
| Communication between Innate and Adaptive Immune Cells | 3.12E-01 | 6.28E-02 | NaN | CCL3,CCR7,CD83,CXCL8,FCER1G,HLA-DRB5,IGHV1-24,IGHV1-46,IGHV1-69,IGHV2-5,IGHV3-20,IGHV3-33,IGHV3-48,IGHV3-66,IGHV3-72,IGHV3-74,IGHV4-4,IGHV7-4-1,IGKV1D-16,IGKV2-24,IGKV2D-29,IGLV3-21,IL1A,IL1RN,IL36A,TLR2,TLR4,TLR8,TNFRSF13B |
| HIF1Œ± Signaling | 3.08E-01 | 6.37E-02 | 0.832 | CAMK2A,EGLN3,FLT1,HK1,HK2,HSPA2,IGF1,IL6R,MMP7,MMP9,NOS2,PIK3R2,SLC2A3 |
| B Cell Activating Factor Signaling | 3.02E-01 | 6.98E-02 | NaN | IKBKE,MAPK13,TNFRSF13B |
| Oncostatin M Signaling | 3.02E-01 | 6.98E-02 | NaN | CHI3L1,OSM,STAT1 |
| Phosphatidylglycerol Biosynthesis II (Non-plastidic) | 2.99E-01 | 7.41E-02 | NaN | AGPAT2,CDS1 |
| Tryptophan Degradation III (Eukaryotic) | 2.99E-01 | 7.41E-02 | NaN | ACAT2,KMO |
| Purine Nucleotides De Novo Biosynthesis II | 2.98E-01 | 9.09E-02 | NaN | ADSS1 |
| Angiopoietin Signaling | 2.95E-01 | 6.58E-02 | 1 | ANGPTL1,FOXO1,IKBKE,PIK3R2,TEK |
| FcŒ≥ Receptor-mediated Phagocytosis in Macrophages and Monocytes | 2.88E-01 | 6.45E-02 | -0.816 | FCGR1A,LYN,PIK3R2,PLD2,SRC,VAV3 |
| Chemokine Signaling | 2.85E-01 | 6.49E-02 | -0.447 | CAMK2A,CXCL12,CXCR4,MAPK13,SRC |
| Inhibition of ARE-Mediated mRNA Degradation Pathway | 2.83E-01 | 6.29E-02 | 0 | MAPK13,MAPK15,MAPK7,PPM1J,PPP2R2B,PPP2R3A,PRKACB,PRKAR1B,TNFSF8,XRN1 |
| Clathrin-mediated Endocytosis Signaling | 2.81E-01 | 6.25E-02 | NaN | AP1M2,AP1S2,APOD,CLTCL1,FGF7,IGF1,PIK3R2,SERPINA1,SH3GL1,SH3GL3,SRC,STON2 |
| Natural Killer Cell Signaling | 2.76E-01 | 6.22E-02 | -0.577 | CD48,FCER1G,HSPA2,IL12RB2,IL18RAP,MAP3K9,MAPK13,MICB,PIK3R2,RAET1L,VAV3,WIPF1 |
| nNOS Signaling in Neurons | 2.64E-01 | 6.52E-02 | NaN | CAMK2A,CAPN1,DLG2 |
| ATM Signaling | 2.63E-01 | 6.25E-02 | 0.816 | CREB5,MAPK13,PPM1J,PPP2R2B,PPP2R3A,ZEB1 |
| TGF-Œ≤ Signaling | 2.63E-01 | 6.25E-02 | 0 | ACVR1B,BCL2,BMP7,MAP4K1,MAPK13,PITX2 |
| ERB2-ERBB3 Signaling | 2.50E-01 | 6.25E-02 | NaN | CCND1,CDKN1B,FOXO1,PIK3R2 |
| Chondroitin Sulfate Degradation (Metazoa) | 2.29E-01 | 7.14E-02 | NaN | ARSB |
| Choline Biosynthesis III | 2.29E-01 | 7.14E-02 | NaN | PLD2 |
| Urate Biosynthesis/Inosine 5'-phosphate Degradation | 2.29E-01 | 7.14E-02 | NaN | ACP3 |
| Phenylalanine Degradation IV (Mammalian, via Side Chain) | 2.29E-01 | 7.14E-02 | NaN | ALDH3A2 |
| Colanic Acid Building Blocks Biosynthesis | 2.29E-01 | 7.14E-02 | NaN | GMDS |
| Telomere Extension by Telomerase | 2.11E-01 | 6.67E-02 | NaN | PINX1 |
| Androgen Biosynthesis | 2.11E-01 | 6.67E-02 | NaN | SRD5A1 |
| D-myo-inositol (1,4,5)-trisphosphate Degradation | 1.94E-01 | 6.25E-02 | NaN | INPP5J |
| Methylglyoxal Degradation III | 1.94E-01 | 6.25E-02 | NaN | PTGR1 |
| Adenosine Nucleotides Degradation II | 1.94E-01 | 6.25E-02 | NaN | ACP3 |
| NAD Signaling Pathway | 0.00E+00 | 4.14E-02 | -0.816 | BST1,H1-0,IGF1,NMNAT3,PIK3R2,SREBF1 |
| CSDE1 Signaling Pathway | 0.00E+00 | 1.82E-02 | NaN | SNAI1 |
| Amyotrophic Lateral Sclerosis Signaling | 0.00E+00 | 6.14E-02 | -1.342 | BCL2,CACNA2D3,CACNB4,CAPN1,CASP3,IGF1,PIK3R2 |
| Actin Cytoskeleton Signaling | 0.00E+00 | 5.83E-02 | -0.707 | ARHGEF4,BAIAP2,FGF7,GSN,ITGA1,ITGA4,ITGAM,ITGAX,LBP,NCKAP1L,PIK3R2,SSH3,TIAM1,VAV3 |
| Huntington's Disease Signaling | 0.00E+00 | 5.43E-02 | -1.667 | BDNF,CAPN1,CASP3,CDK5R1,CLTCL1,CREB5,EGFR,GLS,GNA11,HDAC11,HSPA2,IGF1,PIK3R2,SH3GL3,TGM2 |
| NRF2-mediated Oxidative Stress Response | 0.00E+00 | 6.13E-02 | NaN | CYP2J2,CYP3A5,DNAJB11,DNAJC1,DNAJC10,ENC1,FTL,GSTO2,MAF,MAPK7,MGST1,MGST2,PIK3R2 |
| p53 Signaling | 0.00E+00 | 5.10E-02 | NaN | BCL2,CCND1,DRAM1,HIPK2,PIK3R2 |
| Mitochondrial Dysfunction | 0.00E+00 | 4.24E-02 | NaN | APH1B,BCL2,CASP3,COX4I2,GPX7,MT-ND3,MT-ND4L |
| Role of BRCA1 in DNA Damage Response | 0.00E+00 | 3.75E-02 | NaN | E2F2,E2F8,STAT1 |
| Erythropoietin Signaling Pathway | 0.00E+00 | 5.95E-02 | -1.897 | CCND1,CXCL8,EDA,IL1A,IL36A,MACROH2A2,OSM,PIK3R2,SRC,TNFSF8 |
| Œ±-Adrenergic Signaling | 0.00E+00 | 3.77E-02 | NaN | GNA11,GUCY1B1,PRKACB,PRKAR1B |
| Activation of IRF by Cytosolic Pattern Recognition Receptors | 0.00E+00 | 3.17E-02 | NaN | IKBKE,STAT1 |
| Role of RIG1-like Receptors in Antiviral Innate Immunity | 0.00E+00 | 2.27E-02 | NaN | IKBKE |
| Role of NFAT in Regulation of the Immune Response | 0.00E+00 | 2.62E-02 | 0.333 | FCER1G,FCGR1A,FCGR1B,FCGR2C,GNA11,HLA-DRB5,IKBKE,KPNA5,LYN,PIK3R2 |
| FcŒ≥RIIB Signaling in B Lymphocytes | 0.00E+00 | 4.82E-02 | NaN | CACNA2D3,CACNB4,LYN,PIK3R2 |
| LPS-stimulated MAPK Signaling | 0.00E+00 | 5.88E-02 | -1 | IKBKE,LBP,MAPK13,PIK3R2,TLR4 |
| NF-Œ∫B Activation by Viruses | 0.00E+00 | 3.85E-02 | NaN | CR2,IKBKE,PIK3R2 |
| CCR5 Signaling in Macrophages | 0.00E+00 | 2.02E-02 | NaN | CACNA2D3,CACNB4,CCL3,FCER1G,GNA11,MAPK13 |
| CD40 Signaling | 0.00E+00 | 6.06E-02 | NaN | IKBKE,MAPK13,PIK3R2,PTGS2 |
| Calcium-induced T Lymphocyte Apoptosis | 0.00E+00 | 7.87E-03 | NaN | FCER1G,HLA-DRB5 |
| Cytotoxic T Lymphocyte-mediated Apoptosis of Target Cells | 0.00E+00 | 1.79E-02 | 0 | BCL2,CASP3,FCER1G,GZMB |
| CD27 Signaling in Lymphocytes | 0.00E+00 | 5.45E-02 | NaN | CASP3,IKBKE,MAP3K9 |
| IL-3 Signaling | 0.00E+00 | 3.80E-02 | NaN | FOXO1,PIK3R2,STAT1 |
| fMLP Signaling in Neutrophils | 0.00E+00 | 1.54E-02 | NaN | GNA11,PIK3R2 |
| CXCR4 Signaling | 0.00E+00 | 4.82E-02 | 0.816 | CXCL12,CXCR4,GNA11,GUCY1B1,LYN,PIK3R2,RHOQ,SRC |
| 4-1BB Signaling in T Lymphocytes | 0.00E+00 | 5.88E-02 | NaN | IKBKE,MAPK13 |
| Thrombopoietin Signaling | 0.00E+00 | 3.17E-02 | NaN | PIK3R2,STAT1 |
| CTLA4 Signaling in Cytotoxic T Lymphocytes | 0.00E+00 | 2.89E-02 | NaN | AP1M2,AP1S2,CLTCL1,FCER1G,PIK3R2,PPM1J,PPP2R2B,PPP2R3A |
| T Helper Cell Differentiation | 0.00E+00 | 2.62E-02 | NaN | FCER1G,GATA3,HLA-DRB5,IL12RB2,IL4R,IL6R,STAT1 |
| CCR3 Signaling in Eosinophils | 0.00E+00 | 5.34E-02 | -1.633 | GNA11,JMJD7-PLA2G4B,MAPK13,PIK3R2,PLA2G2F,PLA2G4B,PLA2G4C |
| CD28 Signaling in T Helper Cells | 0.00E+00 | 1.28E-02 | NaN | FCER1G,HLA-DRB5,IKBKE,PIK3R2 |
| Virus Entry via Endocytic Pathways | 0.00E+00 | 5.77E-02 | NaN | AP1M2,AP1S2,CLTCL1,CXADR,PIK3R2,SRC |
| Dendritic Cell Maturation | 0.00E+00 | 6.05E-02 | -0.853 | CCR7,CD83,CREB5,FCER1G,FCGR1A,FCGR1B,FCGR2C,FSCN2,HLA-DRB5,IKBKE,IL1A,IL1RN,IL36A,LTBR,MAPK13,PIK3R2,PLCD1,PLCD3,PLCH2,PLCL2,STAT1,TLR2,TLR4 |
| CNTF Signaling | 0.00E+00 | 5.36E-02 | NaN | PIK3R2,RPS6KA4,STAT1 |
| Agrin Interactions at Neuromuscular Junction | 0.00E+00 | 4.41E-02 | NaN | EGFR,ITGA4,SRC |
| Cardiomyocyte Differentiation via BMP Receptors | 0.00E+00 | 4.55E-02 | NaN | BMP7 |
| Renin-Angiotensin Signaling | 0.00E+00 | 4.96E-02 | -1 | GUCY1B1,MAPK13,PIK3R2,PRKACB,PRKAR1B,STAT1 |
| Semaphorin Signaling in Neurons | 0.00E+00 | 5.08E-02 | NaN | PLXNB1,RHOQ,SEMA3A |
| ICOS-ICOSL Signaling in T Helper Cells | 0.00E+00 | 1.99E-02 | -2 | CAMK2A,FCER1G,HLA-DRB5,IKBKE,PIK3R2,PLEKHA4 |
| Lipid Antigen Presentation by CD1 | 0.00E+00 | 4.67E-03 | NaN | FCER1G |
| HGF Signaling | 0.00E+00 | 6.11E-02 | -1 | CCND1,ITGA1,ITGA4,ITGAM,ITGAX,MAP3K9,PIK3R2,PTGS2 |
| Maturity Onset Diabetes of Young (MODY) Signaling | 0.00E+00 | 5.19E-02 | NaN | APOD,CACNA2D3,CACNB4,KCNJ11 |
| Polyamine Regulation in Colon Cancer | 0.00E+00 | 1.72E-02 | NaN | PPARG |
| Androgen Signaling | 0.00E+00 | 5.36E-02 | -0.816 | CACNA2D3,CACNB4,CCND1,GNA11,GTF2A1,GTF2H2,PRKACB,PRKAR1B,SRC |
| Role of OCT4 in Mammalian Embryonic Stem Cell Pluripotency | 0.00E+00 | 4.44E-02 | NaN | NR2F1,SPP1 |
| Role of NANOG in Mammalian Embryonic Stem Cell Pluripotency | 0.00E+00 | 5.08E-02 | NaN | BMP7,BMP8A,FZD3,PIK3R2,SMO,WNT4 |
| Prolactin Signaling | 0.00E+00 | 2.33E-02 | NaN | PIK3R2,STAT1 |
| Melanoma Signaling | 0.00E+00 | 6.00E-02 | NaN | CCND1,CDH1,PIK3R2 |
| Renal Cell Carcinoma Signaling | 0.00E+00 | 2.56E-02 | NaN | EGLN3,PIK3R2 |
| Type I Diabetes Mellitus Signaling | 0.00E+00 | 2.96E-02 | -1.134 | BCL2,CASP3,FCER1G,GZMB,HLA-DRB5,IKBKE,MAPK13,NOS2,STAT1 |
| Endometrial Cancer Signaling | 0.00E+00 | 5.00E-02 | NaN | CCND1,CDH1,PIK3R2 |
| Primary Immunodeficiency Signaling | 0.00E+00 | 4.00E-02 | NaN | RFXAP,TNFRSF13B |
| Allograft Rejection Signaling | 0.00E+00 | 1.22E-02 | NaN | FCER1G,GZMB,HLA-DRB5 |
| Autoimmune Thyroid Disease Signaling | 0.00E+00 | 1.21E-02 | NaN | FCER1G,GZMB,HLA-DRB5 |
| Acute Myeloid Leukemia Signaling | 0.00E+00 | 3.33E-02 | NaN | CCND1,FLT3,PIK3R2 |
| Graft-versus-Host Disease Signaling | 0.00E+00 | 2.48E-02 | NaN | FCER1G,GZMB,HLA-DRB5,IL1A,IL1RN,IL36A |
| Type II Diabetes Mellitus Signaling | 0.00E+00 | 5.96E-02 | NaN | ACSL4,CACNA2D3,CACNB4,IKBKE,KCNJ11,PIK3R2,PPARG,SLC27A4,SMPD3 |
| mTOR Signaling | 0.00E+00 | 3.90E-02 | 0.378 | PIK3R2,PLD2,PPM1J,PPP2R2B,PPP2R3A,RHOQ,RPS27L,RPS6KA4 |
| G Beta Gamma Signaling | 0.00E+00 | 5.43E-02 | -1.89 | CACNA2D3,CACNB4,EGFR,GNA11,PRKACB,PRKAR1B,SRC |
| G Protein Signaling Mediated by Tubby | 0.00E+00 | 2.33E-02 | NaN | GNA11 |
| Systemic Lupus Erythematosus Signaling | 0.00E+00 | 2.91E-02 | NaN | CREM,FCER1G,FCGR1A,FCGR1B,FCGR2C,IL1A,IL1RN,IL36A,IL6R,LYN,PIK3R2,RNU2-2P |
| CDC42 Signaling | 0.00E+00 | 3.56E-02 | -0.816 | BAIAP2,EXOC6,FCER1G,HLA-DRB5,ITGA1,ITGA4,ITGAM,ITGAX,MAPK13,PARD3,SRC,WIPF1 |
| EIF2 Signaling | 0.00E+00 | 3.29E-02 | 0 | BCL2,CCND1,DDIT3,PIK3R2,RPL39L,RPS27L,SREBF1 |
| Retinoic acid Mediated Apoptosis Signaling | 0.00E+00 | 5.26E-02 | NaN | CASP3,CRABP2,RXRA |
| PAK Signaling | 0.00E+00 | 5.13E-02 | NaN | CASP3,ITGA1,ITGA4,ITGAM,ITGAX,PIK3R2 |
| Hereditary Breast Cancer Signaling | 0.00E+00 | 2.16E-02 | NaN | CCND1,HDAC11,PIK3R2 |
| Phospholipase C Signaling | 0.00E+00 | 4.72E-02 | 0 | ARHGEF4,ARHGEF5,CREB5,FCER1G,FCGR2C,GUCY1B1,HDAC11,ITGA1,ITGA4,ITGAM,ITGAX,JMJD7-PLA2G4B,LYN,PLA2G2F,PLA2G4B,PLA2G4C,PLCD1,PLCD3,PLD2,RHOQ,SRC,TGM2 |
| Altered T Cell and B Cell Signaling in Rheumatoid Arthritis | 0.00E+00 | 4.24E-02 | NaN | CXCL13,FCER1G,HLA-DRB5,IL1A,IL1RN,IL36A,SLAMF1,SPP1,TLR2,TLR4,TLR8,TNFRSF13B |
| Regulation of eIF4 and p70S6K Signaling | 0.00E+00 | 5.68E-02 | 1.342 | ITGA1,ITGA4,ITGAM,ITGAX,MAPK13,PIK3R2,PPM1J,PPP2R2B,PPP2R3A,RPS27L |
| Glioma Invasiveness Signaling | 0.00E+00 | 4.23E-02 | NaN | MMP9,PIK3R2,RHOQ |
| B Cell Development | 0.00E+00 | 2.70E-02 | NaN | HLA-DRB5 |
| Regulation of IL-2 Expression in Activated and Anergic T Lymphocytes | 0.00E+00 | 1.14E-02 | NaN | FCER1G,IKBKE,VAV3 |
| Granzyme A Signaling | 0.00E+00 | 5.88E-02 | NaN | H1-0 |
| Role of WNT/GSK-3Œ≤ Signaling in the Pathogenesis of Influenza | 0.00E+00 | 4.05E-02 | NaN | FZD3,SMO,WNT4 |
| NUR77 Signaling in T Lymphocytes | 0.00E+00 | 2.18E-02 | -0.447 | BCL2,CASP3,FCER1G,HLA-DRB5,MAPK7,RXRA |
| PKCŒ∏ Signaling in T Lymphocytes | 0.00E+00 | 2.55E-02 | -1.89 | CACNA2D3,CACNB4,CAMK2A,FCER1G,HLA-DRB5,IKBKE,MAP3K9,PIK3R2,VAV3 |
| TNFR1 Signaling | 0.00E+00 | 4.00E-02 | NaN | CASP3,IKBKE |
| TNFR2 Signaling | 0.00E+00 | 3.23E-02 | NaN | IKBKE |
| Role of PI3K/AKT Signaling in the Pathogenesis of Influenza | 0.00E+00 | 1.59E-02 | NaN | PIK3R2 |
| Antiproliferative Role of TOB in T Cell Signaling | 0.00E+00 | 8.89E-03 | NaN | CDKN1B,FCER1G |
| OX40 Signaling Pathway | 0.00E+00 | 1.23E-02 | NaN | BCL2,FCER1G,HLA-DRB5 |
| Assembly of RNA Polymerase II Complex | 0.00E+00 | 4.00E-02 | NaN | GTF2A1,GTF2H2 |
| Spliceosomal Cycle | 0.00E+00 | 2.04E-02 | NaN | YJU2 |
| Differential Regulation of Cytokine Production in Macrophages and T Helper Cells by IL-17A and IL-17F | 0.00E+00 | 5.56E-02 | NaN | CCL3 |
| Role of JAK2 in Hormone-like Cytokine Signaling | 0.00E+00 | 3.12E-02 | NaN | STAT1 |
| Role of JAK1 and JAK3 in Œ≥c Cytokine Signaling | 0.00E+00 | 5.97E-02 | NaN | IL4R,IL9R,PIK3R2,STAT1 |
| NGF Signaling | 0.00E+00 | 5.98E-02 | -1.89 | CREB5,IKBKE,MAP3K9,MAPK7,PIK3R2,RPS6KA4,SMPD3 |
| RHOGDI Signaling | 0.00E+00 | 6.13E-02 | 0 | ARHGEF4,ARHGEF5,CDH1,CDH6,DLC1,GNA11,GRIP1,ITGA1,ITGA4,ITGAM,ITGAX,RHOQ,SRC |
| Telomerase Signaling | 0.00E+00 | 5.77E-02 | 0.447 | EGFR,HDAC11,PIK3R2,PPM1J,PPP2R2B,PPP2R3A |
| Mouse Embryonic Stem Cell Pluripotency | 0.00E+00 | 3.88E-02 | -1 | FZD3,MAPK13,PIK3R2,SMO |
| Hematopoiesis from Pluripotent Stem Cells | 0.00E+00 | 1.28E-02 | NaN | CXCL8,FCER1G,IL1A |
| Transcriptional Regulatory Network in Embryonic Stem Cells | 0.00E+00 | 1.85E-02 | NaN | MEIS1 |
| eNOS Signaling | 0.00E+00 | 4.61E-02 | 1.134 | CASP3,FLT1,GUCY1B1,HSPA2,PIK3R2,PRKACB,PRKAR1B |
| nNOS Signaling in Skeletal Muscle Cells | 0.00E+00 | 4.26E-02 | NaN | CACNA2D3,CACNB4 |
| ERBB Signaling | 0.00E+00 | 4.30E-02 | -2 | EGFR,FOXO1,MAPK13,PIK3R2 |
| ERBB4 Signaling | 0.00E+00 | 3.03E-02 | NaN | APH1B,PIK3R2 |
| GADD45 Signaling | 0.00E+00 | 5.26E-02 | NaN | CCND1 |
| GDNF Family Ligand-Receptor Interactions | 0.00E+00 | 3.95E-02 | NaN | DOK4,GFRA1,PIK3R2 |
| Heparan Sulfate Biosynthesis | 0.00E+00 | 6.06E-02 | 2 | B3GAT2,CHST1,CHST15,EXTL2 |
| Nicotine Degradation III | 0.00E+00 | 6.00E-02 | NaN | CYP19A1,CYP2J2,CYP3A5 |
| Heparan Sulfate Biosynthesis (Late Stages) | 0.00E+00 | 5.08E-02 | NaN | CHST1,CHST15,EXTL2 |
| Pyrimidine Ribonucleotides De Novo Biosynthesis | 0.00E+00 | 2.86E-02 | NaN | AK4 |
| Glutaryl-CoA Degradation | 0.00E+00 | 5.00E-02 | NaN | ACAT2 |
| Melatonin Degradation I | 0.00E+00 | 5.56E-02 | NaN | CYP19A1,CYP2J2,CYP3A5 |
| Pyridoxal 5'-phosphate Salvage Pathway | 0.00E+00 | 4.62E-02 | NaN | MAP3K9,MAPK7,NEK2 |
| Pyrimidine Deoxyribonucleotides De Novo Biosynthesis I | 0.00E+00 | 4.35E-02 | NaN | AK4 |
| Triacylglycerol Degradation | 0.00E+00 | 4.76E-02 | NaN | FAAH,MGLL |
| Chondroitin Sulfate Biosynthesis | 0.00E+00 | 5.66E-02 | NaN | B3GAT2,CHST1,CHST15 |
| Valine Degradation I | 0.00E+00 | 4.76E-02 | NaN | BCAT1 |
| Serotonin Degradation | 0.00E+00 | 5.26E-02 | NaN | ALDH3A1,ALDH3A2,ALDH4A1 |
| Retinol Biosynthesis | 0.00E+00 | 4.88E-02 | NaN | RDH12,RDH13 |
| Salvage Pathways of Pyrimidine Ribonucleotides | 0.00E+00 | 4.17E-02 | -2 | AK4,MAP3K9,MAPK7,NEK2 |
| Pyrimidine Ribonucleotides Interconversion | 0.00E+00 | 3.03E-02 | NaN | AK4 |
| Glycolysis I | 0.00E+00 | 4.17E-02 | NaN | PFKP |
| Gluconeogenesis I | 0.00E+00 | 4.17E-02 | NaN | ME1 |
| Chondroitin Sulfate Biosynthesis (Late Stages) | 0.00E+00 | 4.44E-02 | NaN | CHST1,CHST15 |
| Superpathway of Melatonin Degradation | 0.00E+00 | 5.08E-02 | NaN | CYP19A1,CYP2J2,CYP3A5 |
| Histidine Degradation VI | 0.00E+00 | 5.56E-02 | NaN | HAL |
| Fatty Acid Œ≤-oxidation I | 0.00E+00 | 5.88E-02 | NaN | ACSL4,SLC27A4 |
| GŒ±i Signaling | 0.00E+00 | 5.71E-02 | 0.816 | APLNR,GNA11,GUCY1B1,PRKACB,PRKAR1B,RGS12,S1PR3,SRC |
| GŒ±q Signaling | 0.00E+00 | 4.85E-02 | -1.414 | AVPR1A,GNA11,IKBKE,PIK3R2,PLD2,RGS18,RGS2,RHOQ |
| Remodeling of Epithelial Adherens Junctions | 0.00E+00 | 6.06E-02 | NaN | CDH1,SRC,TUBB2B,TUBB6 |
| Role of p14/p19ARF in Tumor Suppression | 0.00E+00 | 3.57E-02 | NaN | PIK3R2 |
| TEC Kinase Signaling | 0.00E+00 | 3.73E-02 | 0 | FCER1G,GNA11,ITGA1,ITGA4,ITGAM,ITGAX,LYN,PIK3R2,RHOQ,SRC,STAT1,TLR4,TNFRSF25,VAV3 |
| UVB-Induced MAPK Signaling | 0.00E+00 | 5.77E-02 | NaN | EGFR,MAPK13,PIK3R2 |
| Oxidative Phosphorylation | 0.00E+00 | 2.83E-02 | NaN | COX4I2,MT-ND3,MT-ND4L |
| Estrogen Receptor Signaling | 0.00E+00 | 6.02E-02 | -0.688 | BCL2,CACNA2D3,CACNB4,CCND1,CREB5,EGFR,FOXO1,GNA11,GUCY1B1,HES1,IGF1,MMP7,MMP9,MT-ND3,MT-ND4L,PIK3R2,PLCD1,PLCD3,PLCH2,PLCL2,PRKACB,PRKAR1B,SNAI1,SRC |
| Nucleotide Excision Repair Pathway | 0.00E+00 | 2.86E-02 | NaN | GTF2H2 |
| SAPK/JNK Signaling | 0.00E+00 | 1.66E-02 | -0.447 | FCER1G,MAP3K9,MAP4K1,MINK1,PIK3R2 |
| Protein Ubiquitination Pathway | 0.00E+00 | 3.33E-02 | NaN | DNAJB11,DNAJC1,DNAJC10,DNAJC25,HSPA2,HSPB1,PAN2,SMO,USP2 |
| IL-2 Signaling | 0.00E+00 | 1.64E-02 | NaN | PIK3R2 |
| FGF Signaling | 0.00E+00 | 5.95E-02 | -0.447 | CREB5,FGF7,FGFR3,MAPK13,PIK3R2 |
| JAK/STAT Signaling | 0.00E+00 | 2.44E-02 | NaN | PIK3R2,STAT1 |
| Cell Cycle: G2/M DNA Damage Checkpoint Regulation | 0.00E+00 | 4.00E-02 | NaN | HIPK2,PKMYT1 |
| GABA Receptor Signaling | 0.00E+00 | 3.79E-02 | NaN | CACNA2D3,CACNB4,GABRQ,GNA11,GUCY1B1 |
| IL-4 Signaling | 0.00E+00 | 4.44E-02 | NaN | HLA-DRB5,IL4R,INPP5J,PIK3R2 |
| Antigen Presentation Pathway | 0.00E+00 | 2.63E-02 | NaN | HLA-DRB5 |
| Serotonin Receptor Signaling | 0.00E+00 | 4.55E-02 | NaN | GUCY1B1,SLC6A4 |
| Neurotrophin/TRK Signaling | 0.00E+00 | 3.95E-02 | NaN | BDNF,CREB5,PIK3R2 |
| Integrin Signaling | 0.00E+00 | 5.85E-02 | 0.632 | ARF4,CAPN1,GSN,ITGA1,ITGA4,ITGAM,ITGAX,PIK3R2,RHOQ,SRC,TSPAN2,WIPF1 |
| Death Receptor Signaling | 0.00E+00 | 5.32E-02 | -0.447 | BCL2,CASP3,HSPB1,IKBKE,TNFRSF25 |
| IGF-1 Signaling | 0.00E+00 | 5.77E-02 | 0 | CCN2,FOXO1,IGF1,PIK3R2,PRKACB,PRKAR1B |
| Glutamate Receptor Signaling | 0.00E+00 | 4.62E-02 | NaN | GLS,GRIP1,HOMER2 |
| Apoptosis Signaling | 0.00E+00 | 4.90E-02 | -0.447 | BCL2,BCL2A1,CAPN1,CASP3,IKBKE |
| NF-Œ∫B Signaling | 0.00E+00 | 3.81E-02 | 0 | EGFR,FCER1G,FGFR3,FLT1,IL1A,IL1RN,IL36A,IRAK3,LTBR,PIK3R2,PRKACB,TLR2,TLR4,TLR8 |
| VEGF Signaling | 0.00E+00 | 5.15E-02 | 0 | BCL2,FLT1,FOXO1,PIK3R2,SRC |
| Hypoxia Signaling in the Cardiovascular System | 0.00E+00 | 1.39E-02 | NaN | CREB5 |
| T Cell Receptor Signaling | 0.00E+00 | 1.86E-02 | -1.89 | CDKN1B,FCER1G,HLA-DRB5,IKBKE,MAPK13,PIK3R2,VAV3 |
| PDGF Signaling | 0.00E+00 | 4.65E-02 | -2 | INPP5J,PIK3R2,SRC,STAT1 |
| Phagosome Maturation | 0.00E+00 | 4.03E-02 | NaN | CTSH,CTSS,HLA-DRB5,RAB7B,TUBB2B,TUBB6 |
| Macropinocytosis Signaling | 0.00E+00 | 2.63E-02 | NaN | PIK3R2,SRC |
| PD-1, PD-L1 cancer immunotherapy pathway | 0.00E+00 | 2.91E-02 | NaN | CDKN1B,HLA-DRB5,PIK3R2 |
| Cancer Drug Resistance By Drug Efflux | 0.00E+00 | 5.17E-02 | NaN | FOXO1,PIK3R2,PTGS2 |
| Sumoylation Pathway | 0.00E+00 | 4.95E-02 | -1 | CDH1,RANGAP1,RHOQ,SP3,ZEB1 |
| Sirtuin Signaling Pathway | 0.00E+00 | 5.63E-02 | 0.277 | ATG9B,CDH1,CXCL8,FOXO1,GLS,H1-0,MAPK15,MAPK7,MT-ND3,MT-ND4L,NOS2,PPARG,PPIF,SCNN1A,SREBF1,TIMM13 |
| Iron homeostasis signaling pathway | 0.00E+00 | 5.26E-02 | NaN | BMP7,BMP8A,EGFR,FTL,GDF15,IL6R,SLC46A1 |
| Th17 Activation Pathway | 0.00E+00 | 1.78E-02 | 0.447 | DEFB4A/DEFB4B,FCER1G,IL12RB2,IL6R,IRAK3 |
| NER (Nucleotide Excision Repair, Enhanced Pathway) | 0.00E+00 | 1.96E-02 | NaN | CHAF1A,GTF2H2 |
| SPINK1 General Cancer Pathway | 0.00E+00 | 4.92E-02 | NaN | EGFR,IL6R,PIK3R2 |
| Apelin Liver Signaling Pathway | 0.00E+00 | 3.85E-02 | NaN | APLNR |
| Apelin Endothelial Signaling Pathway | 0.00E+00 | 4.29E-02 | 0 | APLNR,GNA11,GUCY1B1,KLF2,PIK3R2,TEK |
| Apelin Muscle Signaling Pathway | 0.00E+00 | 4.17E-02 | NaN | APLNR,GNA11 |
| BAG2 Signaling Pathway | 0.00E+00 | 3.70E-02 | NaN | BAG2,CASP3,HSPA2 |
| FAT10 Cancer Signaling Pathway | 0.00E+00 | 6.00E-02 | NaN | ACVR1B,CXCR4,IKBKE |
| T Cell Exhaustion Signaling Pathway | 0.00E+00 | 3.33E-02 | -0.302 | ACVR1B,FCER1G,FOXO1,GZMB,HLA-DRB5,IL12RB2,IL6R,PIK3R2,PPM1J,PPP2R2B,PPP2R3A,STAT1 |
| IL-23 Signaling Pathway | 0.00E+00 | 4.35E-02 | NaN | IL12RB2,PIK3R2 |
| Systemic Lupus Erythematosus In T Cell Signaling Pathway | 0.00E+00 | 3.23E-02 | -0.832 | CASP3,CREB5,CREM,FCER1G,HLA-DRB5,NOS2,PIK3R2,PPM1J,PPP2R2B,PPP2R3A,RHOQ,S1PR3,SELPLG |
| HOTAIR Regulatory Pathway | 0.00E+00 | 4.40E-02 | 1.134 | CDH1,MMP7,MMP9,PIK3R2,SPP1,TLR4,WNT4 |
| Calcium Signaling | 0.00E+00 | 4.33E-02 | -1.134 | CACNA2D3,CACNB4,CAMK2A,CAMKK1,CREB5,HDAC11,PRKACB,PRKAR1B,TRPV6 |
|  |  |  |  |  |
|  |  |  |  |  |
