# Supplementary material for: Distinctive genes and signaling pathways associated with type 2 diabetes-related periodontitis: Preliminary study
Source: PLoS One. 2024 Jan 19;19(1):e0296925. doi: 10.1371/journal.pone.0296925 (PMC10798476; doi:10.1371/journal.pone.0296925)
Supplement: Attachment — Submitted filename: PONE-D-23-19661_revised.pdf [file pone.0296925.s008.pdf]

PLOS ONE

Distinctive genes and signaling pathways associated with type 2 diabetes-related periodontitis: a preliminary study

--Manuscript Draft--

**Manuscript Number:**	PONE-D-23-19661
**Article Type:**	Research Article
**Full Title:**	Distinctive genes and signaling pathways associated with type 2 diabetes-related periodontitis: a preliminary study
**Short Title:**	Transcriptome analyses of diabetes-related periodontitis
**Corresponding Author:**	Poliana Mendes Duarte<br>University of Florida College of Dentistry<br>Gainesville, UNITED STATES
**Keywords:**	
**Abstract:**	The biological mechanisms underlying the pathogenesis of type 2 diabetes (T2DM)-related periodontitis remain unclear. This cross-sectional study evaluated the distinctive transcriptomic changes between tissues with periodontal health and with periodontitis in patients with T2DM. In this cross-sectional study, whole transcriptome sequencing was performed on gingival biopsies from non-periodontitis and periodontitis tissues from non-diabetic and diabetic patients. A differentially expressed gene (DEG) analysis and Ingenuity Pathway Analysis (IPA) assessed the genes and signaling pathways associated with T2DM-related periodontitis. Immunohistochemistry was performed to validate selected DEGs possibly involved in T2DM-related periodontitis. Four hundred and twenty and one thousand five hundred and sixty-three DEGs (fold change ≥ 2) were uniquely identified in the diseased tissues of non-diabetic and diabetic patients, respectively. The IPA predicted the activation of Phagosome Formation, Cardiac β-adrenergic, tRNA Splicing, and PI3K/AKT pathways. The IPA also predicted the inhibition of Cholesterol Biosynthesis, Adrenomedullin, and Inositol Phosphate Compounds pathways in T2DM-related periodontitis. Validation of DEGs confirmed changes in protein expression of PTPN2, PTPN13, DHCR24, PIK3R2, CALCRL, IL1RN, IL-6R and ITGA4 in diseased tissues in diabetic subjects. Thus, these preliminary findings indicate that there are specific genes and functional pathways that may be involved in the pathogenesis of T2DM-related periodontitis.
**Order of Authors:**	Poliana Mendes Duarte
	Bruno César de Vasconcelos Gurgel
	Tamires Szeremeske Miranda
	Juliana Sardenberg
	Tongjun Gu
	Ikramuddin Aukhil
**Additional Information:**	
**Question**	**Response**
**Financial Disclosure**<br><br>Enter a financial disclosure statement that describes the sources of funding for the work included in this submission. Review the submission guidelines for detailed requirements. View published research articles from *PLOS ONE* for specific examples.	PMD received a University of Florida College of Dentistry Faculty grant. The funders had no role in study design, data collection and analysis, decision to publish, or preparation of the manuscript.

This statement is required for submission and **will appear in the published article** if the submission is accepted. Please make sure it is accurate.<br><br>**Unfunded studies**<br>Enter: *The author(s) received no specific funding for this work.*<br><br>**Funded studies**<br>Enter a statement with the following details:<br>• Initials of the authors who received each award<br>• Grant numbers awarded to each author<br>• The full name of each funder<br>• URL of each funder website<br>• Did the sponsors or funders play any role in the study design, data collection and analysis, decision to publish, or preparation of the manuscript?<br>• **NO** - Include this sentence at the end of your statement: *The funders had no role in study design, data collection and analysis, decision to publish, or preparation of the manuscript.*<br>• **YES** - Specify the role(s) played.<br><br>* typeset	
**Competing Interests**<br><br>Use the instructions below to enter a competing interest statement for this submission. On behalf of all authors, disclose any competing interests that could be perceived to bias this work—acknowledging all financial support and any other relevant financial or non-financial competing interests.<br><br>This statement is required for submission and **will appear in the published article** if the submission is accepted. Please make sure it is accurate and that any funding sources listed in your Funding Information later in the submission form are also declared in your Financial Disclosure statement.<br><br>View published research articles from *PLOS ONE* for specific examples.	The authors have declared that no competing interests exist.

Powered by Editorial Manager® and ProduXion Manager® from Aries Systems Corporation

**NO authors have competing interests**<br><br>Enter: *The authors have declared that no competing interests exist.*<br><br>**Authors with competing interests**<br><br>Enter competing interest details beginning with this statement:<br><br>*I have read the journal's policy and the authors of this manuscript have the following competing interests: [insert competing interests here]*<br><br>* typeset	
**Ethics Statement**<br><br>Enter an ethics statement for this submission. This statement is required if the study involved:<br><br>• Human participants<br>• Human specimens or tissue<br>• Vertebrate animals or cephalopods<br>• Vertebrate embryos or tissues<br>• Field research<br><br>Write "N/A" if the submission does not require an ethics statement.<br><br>General guidance is provided below. Consult the submission guidelines for detailed instructions. **Make sure that all information entered here is included in the Methods section of the manuscript.**	The study was approved by the University of Florida's Institutional Review Board (IRB201901176)

**Format for specific study types**<br><br>**Human Subject Research (involving human participants and/or tissue)**<br>• Give the name of the institutional review board or ethics committee that approved the study<br>• Include the approval number and/or a statement indicating approval of this research<br>• Indicate the form of consent obtained (written/oral) or the reason that consent was not obtained (e.g. the data were analyzed anonymously)<br><br>**Animal Research (involving vertebrate animals, embryos or tissues)**<br>• Provide the name of the Institutional Animal Care and Use Committee (IACUC) or other relevant ethics board that reviewed the study protocol, and indicate whether they approved this research or granted a formal waiver of ethical approval<br>• Include an approval number if one was obtained<br>• If the study involved *non-human primates*, add *additional details* about animal welfare and steps taken to ameliorate suffering<br>• If anesthesia, euthanasia, or any kind of animal sacrifice is part of the study, include briefly which substances and/or methods were applied<br><br>**Field Research**<br><br>Include the following details if this study involves the collection of plant, animal, or other materials from a natural setting:<br>• Field permit number<br>• Name of the institution or relevant body that granted permission	
**Data Availability**<br><br>Authors are required to make all data underlying the findings described fully available, without restriction, and from the time of publication. PLOS allows rare exceptions to address legal and ethical concerns. See the PLOS Data Policy and FAQ for detailed information.	Yes - all data are fully available without restriction

Powered by Editorial Manager® and ProduXion Manager® from Aries Systems Corporation

A Data Availability Statement describing where the data can be found is required at submission. Your answers to this question constitute the Data Availability Statement and **will be published in the article**, if accepted.<br><br>**Important:** Stating 'data available on request from the author' is not sufficient. If your data are only available upon request, select 'No' for the first question and explain your exceptional situation in the text box.<br><br>Do the authors confirm that all data underlying the findings described in their manuscript are fully available without restriction?	
**Describe where the data may be found in full sentences. If you are copying our sample text, replace any instances of XXX with the appropriate details.**<br><br>• If the data are **held or will be held in a public repository**, include URLs, accession numbers or DOIs. If this information will only be available after acceptance, indicate this by ticking the box below. For example: *All XXX files are available from the XXX database (accession number(s) XXX, XXX.)*.<br>• If the data are all contained **within the manuscript and/or Supporting Information files**, enter the following: *All relevant data are within the manuscript and its Supporting Information files.*<br>• If neither of these applies but you are able to provide **details of access elsewhere**, with or without limitations, please do so. For example:<br><br>*Data cannot be shared publicly because of [XXX]. Data are available from the XXX Institutional Data Access / Ethics Committee (contact via XXX) for researchers who meet the criteria for access to confidential data.*<br><br>*The data underlying the results presented in the study are available from (include the name of the third party*	All relevant data are within the manuscript and its Supporting Information files.

and contact information or URL).<br>• This text is appropriate if the data are owned by a third party and authors do not have permission to share the data.<br><br>* typeset	
Additional data availability information:	

Powered by Editorial Manager® and ProduXion Manager® from Aries Systems Corporation

Manuscript Click here to access/download;Manuscript;Duarte PM Plos One.docx

Distinctive genes and signaling pathways associated with type 2 diabetes-related periodontitis: a preliminary study

Running title: Transcriptome analyses of diabetes-related periodontitis

Poliana Mendes Duarte[1*], Bruno César de Vasconcelos Gurgel[2], Tamires Szeremeske Miranda[3], Juliana Sardenberg[1], Tongjun Gu[4], Ikramuddin Aukhil[1]

*** Corresponding authors**

Poliana Mendes Duarte – pmendesduarte@dental.ufl.edu

University of Florida
Department of Periodontology
1600 SW Archer Rd.
Gainesville, FL, USA
Zip Code: 32610
Phone: +1 352- 273-6192 / Fax: Fax: +1 352-273-6192

[1]Department of Periodontology, University of Florida College of Dentistry; Gainesville, FL, USA, 32610

[2] Department of Dentistry, Federal University of Rio Grande do Norte; Natal, RN, Brazil, 59072-970

[3] Department of Dentistry, São Judas Tadeu University; São Paulo, SP, Brazil, 03166-000

[4] ICBR Bioinformatics, University of Florida; Gainesville, FL, USA, 32610

Number of tables: 2 tables; 6 supplemental tables
Number of figures: 5 figures; 1 supplemental figure

Abstract

The biological mechanisms underlying the pathogenesis of type 2 diabetes (T2DM)-related periodontitis remain unclear. This cross-sectional study evaluated the distinctive transcriptomic changes between tissues with periodontal health and with periodontitis in patients with T2DM. In this cross-sectional study, whole transcriptome sequencing was performed on gingival biopsies from non-periodontitis and periodontitis tissues from non-diabetic and diabetic patients. A differentially expressed gene (DEG) analysis and Ingenuity Pathway Analysis (IPA) assessed the genes and signaling pathways associated with T2DM-related periodontitis. Immunohistochemistry was performed to validate selected DEGs possibly involved in T2DM-related periodontitis. Four hundred and twenty and one thousand five hundred and sixty-three DEGs (fold change ≥ 2) were uniquely identified in the diseased tissues of non-diabetic and diabetic patients, respectively. The IPA predicted the activation of Phagosome Formation, Cardiac β-adrenergic, tRNA Splicing, and PI3K/AKT pathways. The IPA also predicted the inhibition of Cholesterol Biosynthesis, Adrenomedullin, and Inositol Phosphate Compounds pathways in T2DM-related periodontitis. Validation of DEGs confirmed changes in protein expression of PTPN2, PTPN13, DHCR24, PIK3R2, CALCRL, IL1RN, IL-6R and ITGA4 in diseased tissues in diabetic subjects. Thus, these preliminary findings indicate that there are specific genes and functional pathways that may be involved in the pathogenesis of T2DM-related periodontitis.

Introduction

Diabetes mellitus (DM) has long been documented as a risk factor for periodontitis, a highly prevalent infectious-inflammatory disease that can cause periodontal tissue breakdown and, ultimately, tooth loss [1,2]. DM is considered a disease grade modifier by the classification system of periodontal diseases, used as a critical indicator of periodontitis progression [3].

Previous studies have attempted to ascertain the changes in specific transcripts and proteins associated with the pathogenesis of DM-related periodontitis [4]. Overall evidence indicates that chronic hyperglycemia may stimulate the pathophysiological determinants of periodontitis onset and progression, including microvascular dysfunction, oxidative stress, hyperinflammation, and dysregulation of cell dynamics [5]. Nevertheless, the specific genes and biological pathways underlying the pathogenesis of type 2 DM (T2DM)-related periodontitis are still undetermined. To date, most of the studies focusing on the pathogenesis of the T2DM-related periodontitis had restricted gene or protein coverage due to the use of low- to mid-plex or microarray-based techniques, thus, failing to assess the biological processes involved in T2DM-related periodontitis comprehensively.

The emergence of high-throughput technologies and bioinformatic approaches enhanced the possibility of understanding the complexity of the eukaryotic transcriptome related to several diseases and identifying broader gene expression profiles, specific signaling pathways, and novel transcript variants [6]. To the best of our knowledge, there are no previous studies on whole transcriptome datasets from

human gingival biopsies of patients with periodontitis and T2DM. Thus, the aim of this study is to assess the changes in the whole transcriptome that occur from periodontal health to periodontitis in patients with T2DM. We hypothesize that diabetic and non-diabetic patients exhibit significant differences in gene expression profiles that may translate into different immunoinflammatory responses and, ultimately, distinct susceptibility to periodontal breakdown. The transcriptional characterization of the T2DM-related periodontitis is important to identify key genes and signaling pathways that can be used as targets for diagnosis, preventive, and therapeutic strategies in the future.

Results

The mean age and frequency of sex did not differ among the groups ($P > 0.05$; table S1). The periodontal parameters were significantly higher in both groups with periodontitis compared with the groups without periodontitis ($P < 0.05$; table S1). There were no differences between non-diabetic patients without periodontitis (HH) and T2DM patients without periodontitis (DH) and between non-diabetic patients with periodontitis (HP) and T2DM patients with periodontitis (DP) in relation to the periodontal parameters ($P > 0.05$; table S1)

Differentially expressed genes (DEGs) analysis

Fig. S1 presents the Venn diagram of the differentially expressed genes (DEGs) in the non-diabetic (HH vs. HP) and diabetic (DH vs. DP) patients exclusively and the overlapping DEGs between the two groups. Four hundred and twenty DEGs (table

S2; 211 upregulated and 209 downregulated) were identified in tissues with periodontitis compared with those with periodontal health in non-diabetic patients from the 16,164 background genes. One thousand five hundred and sixty-three DEGs (table S3; 654 upregulated and 909 downregulated) were identified in tissues with periodontitis compared with those with periodontal health in diabetic patients from the 16,299 background genes. One thousand two hundred and forty-two DEGs were concomitantly expressed in tissues with periodontitis in the non-diabetic and diabetic patients (table S4; 708 upregulated and 534 downregulated).

The top 15 up and downregulated DEGs in the non-diabetic (HP vs. HH) and diabetic (DP vs. DH) groups are listed in Table 1. Overall, immunoglobulin (Ig)-related genes were highly expressed in the non-diabetic and diabetic patients. Excluding the Ig-related DEGs, inhibin βE (INHBE) and C-X-C motif chemokine ligand 5 (CXCL5) were the most upregulated DEGs in non-diabetic and diabetic patients, respectively. Adhesion G protein-coupled receptor G7 (ADGRG7) and AL356488.2 were the DEGs with the strongest downregulation in non-diabetic and diabetic patients, respectively. Five genes from the late cornified envelope (LCE) family were amongst the top 15 downregulated DEGs in tissues with periodontitis in diabetic patients.

Table 1 - Top 15 downregulated and upregulated DEGs in periodontitis in relation to periodontal health in non-diabetic and in patients with T2DM exclusively.

	Downregulated				Upregulated			
	Gene	Q value	P value	Log2 FC	Gene	Q value	P value	Log2 FC
DEGs exclusively in non-diabetics (HP vs HH)	ADGRG7	6.08E-05	1.47E-06	-9.65	IGKV1D-17	0.0071315	0.000790428	4.80
	AADAC	0.00024201	9.12E-06	-7.02	IGLV10-54	0.002906844	0.000228862	4.67
	TYRP1	8.23E-06	1.17E-07	-5.22	IGKV5-2	9.14E-06	1.34E-07	4.39
	CCER2	0.0001552	4.92E-06	-4.77	MTND2P28 (lnc) ENSG00000225630	0.001346243	8.27E-05	3.75
	AC036176.3 (lnc) ENSG00000269989	3.60E-05	7.64E-07	-4.10	IGKV6D-21	1.07E-07	4.19E-10	3.54
	DCT	0.00022835	8.50E-06	-3.76	IGHV4-31	0.000355956	1.51E-05	3.24
	PAX1	0.00143649	8.98E-05	-3.32	INHBE	0.000189732	6.60E-06	3.20
	AC073896.1	0.00016101	5.27E-06	-3.20	AC026202.2 (lnc) ENSG00000233912	1.74E-06	1.65E-08	2.99
	TM4SF19	0.00018363	6.28E-06	-3.20	CBSL	0.009032748	0.001093897	2.97
	FOSB	1.74E-06	1.64E-08	-2.93	IGLV3-9	0.003098993	0.000247676	2.89
	ARC	0.00528802	0.00051847	-2.92	CU633967.1 (lnc) ENSG00000274333	2.61E-07	1.48E-09	2.79

	NPIPA8	0.03595036	0.00706707	-2.86	AMPD1	1.23E-05	1.94E-07	2.77
	SMARCA5-AS1 (lnc) ENSG00000245112	0.00016881	5.61E-06	-2.82	IGKV2D-30	0.00849601	0.00100104	2.73
	AC126773.2 (lnc) ENSG00000260577	0.0007186	3.67E-05	-2.77	MAP3K19	3.52E-05	7.38E-07	2.70
	AC106886.2 (lnc) ENSG00000260899	0.0001565	4.99E-06	-2.71	JCHAIN	8.20E-05	2.19E-06	2.60
DEGs exclusivaly in diabetics (DP vs DH)	SCARNA2 (lnc) ENSG00000270066	5.91E-26	2.18E-29	-10.8	IGHV3-20	4.21E-09	8.12E-11	7.12
	LCE2C	7.91E-08	2.34E-09	-9.89	IGHV1-69	0.00040398	4.51E-05	5.46
	AC105001.2	6.80E-13	4.26E-15	-9.88	IGHV3-72	1.36E-08	3.08E-10	5.44
	LCE2A	9.99E-06	5.82E-07	-9.05	IGHV3-33	5.08E-10	7.47E-12	4.80
	LCE2B	2.43E-06	1.15E-07	-8.93	IGKV1D-16	0.0001974	1.94E-05	4.66
	AC138811.2	1.47E-07	4.67E-09	-8.73	IGHV3-48	3.36E-13	1.94E-15	4.22
	LCE2D	3.71E-05	2.71E-06	-8.688	IGHV3-74	4.01E-16	8.12E-19	4.04
	SMAD1-AS1(lnc) ENSG00000250902	0.00034329	3.72E-05	-8.63	IGKV2D-29	1.29E-05	7.76E-07	3.48

SENP3-EIF4A1(lnc) ENSG00000277957	1.47E-05	9.02E-07	-8.30	AC244226.1	0.00201771	0.00029938	3.46
ATP12A	5.27E-06	2.79E-07	-8.25	CXCL5	0.0001365	1.25E-05	3.45
KLRG2	1.98E-05	1.29E-06	-7.96	IGHV3-66	0.028498749	0.007819436	3.21
AP000350.6 (lnc) ENSG00000273295	6.86E-06	3.79E-07	-6.57	IGHV1-46	2.04E-05	1.33E-06	3.18
LCE3C	2.03E-06	9.40E-08	-5.79	IGLV3-21	0.003652181	0.000613812	3.18
KRT76	0.00013426	1.23E-05	-5.19	P2RX5	7.01E-16	1.55E-18	3.15
PLA2G2F	3.51E-05	2.54E-06	-4.97	GDF15	5.80E-07	2.25E-08	3.02

Ingenuity Pathway Analysis (IPA)

The list of ingenuity canonical pathways related to the DEGs that were uniquely expressed in non-diabetic patients is presented in table S5. Twenty-one ingenuity canonical pathways were identified in non-diabetics by applying a −log (*P*-value) threshold of greater than two. The top ten representative pathways ranked according to the −log (*P* - value) are presented in Fig. 1A. B Cell Receptor Signaling was the highest-ranking signaling pathway in the non-diabetic patients. None of the significant signaling pathways in non-diabetic patients presented a Z-score over 2 or under −2. Considering a Z-score of less than −1.3 as significant, the Tumor Microenvironment and PI3K Signaling in B Lymphocytes pathways were significantly inhibited (Fig. 1, B and C). Within these enriched pathways, FOS, JUN, activating transcription factor 3 (ATF3), and matrix metalloproteinase (MMP)-13 were the DEGs with a log2 fold change (FC) less than or equal to −1.5.

Fig. 1. IPA analysis of the canonical pathways related to the DEGs altered in tissues with periodontitis compared with tissues with periodontal health in non-diabetic patients. (A) The top ten dysregulated canonical signaling pathways are presented in descending order of statistical significance of −log(*P*-values). (B) The canonical pathways that were predicted to be modulated in the non-diabetic patients, as defined by a −log(*P*-value) >2 and Z-score <−1.3 (inhibited). (C) The number of

upregulated and downregulated DEGs involved in each of the representative canonical pathways.

The list of ingenuity canonical pathways related to the DEGs exclusively expressed in diabetic patients is presented in table S6. Forty-three ingenuity canonical pathways were identified by applying a −log (*P*-value) threshold of greater than two. The top ten signaling pathways are presented in Fig. 2A. Granulocyte Adhesion and Diapedesis was the highest-ranking pathway (−log [*P*-value] = 5.55), followed by Phagosome Formation (−log [*P*-value] = 4.75) and Atherosclerosis Signaling (−log [*P*-value] = 4.55). Taking Z-scores greater than 2 and less than −2, Phagosome Formation, Cardiac β-adrenergic, tRNA, and PI3K/AKT signaling pathways were significantly activated. In contrast, the Superpathway of Cholesterol Biosynthesis, Adrenomedullin (ADM), and Superpathway of Inositol Phosphate Compounds (SIPC) were inhibited in tissues with periodontitis in the diabetic patients (Fig. 2, B and C).

Fig. 2. IPA analysis of the canonical pathways related to the DEGs altered in tissues with periodontitis compared with tissues with periodontal health in patients with T2DM. (A) The top ten dysregulated canonical signaling pathways are presented in descending order of statistical significance of −log(*P*-values). (B) The canonical pathways that were predicted to be modulated in the non-diabetic patients, as defined by a −log(*P*-value) >2 and Z-score >2 (activated) or <−2 (inhibited). (C)

The number of upregulated and downregulated DEGs involved in each of the representative canonical pathways.

Table 2 presents the DEGs that function within the abovementioned canonical pathways in diabetic patients. The upregulated DEGs that function in the Phagosome Formation pathway included mostly adhesion G protein-coupled receptors (GPCRs), complement-related factors, chemokine, pathogen pattern-recognition and Ig receptors, and integrin (ITG) subunits. The downregulated DEGs involved in this pathway were comprised mainly of GPCRs, mitogen-activated protein kinase (MAPK), and phospholipases (PL). ITG subunits were the predominant upregulated gene group, while interleukin (IL) receptors and phosphatase-related components were the leading downregulated DEGs functioning in PI3K/AKT signaling. Most of the upregulated genes in the Cardiac β-Adrenergic and tRNA Splicing pathways were phosphodiesterases. Of the 12 genes downregulated in Cardiac β-Adrenergic Signaling, six were phosphatase-related, and two were calcium voltage-gated channel auxiliary-related genes. The primary downregulated genes observed in the ADM pathway were comprised of MAPKs, Phospholipase C (PLC), and transcription factors. Notably, in the ADM pathway, IL1A was upregulated while IL1 receptor antagonist (IL1RN) was downregulated in the diabetic patients. The mRNA for different enzyme functional classes were downregulated in Superpathway of Cholesterol Biosynthesis signaling. Phosphatase and PL genes were generally downregulated in SIPC signaling.

Table 2 - The DEGs that are a part of the most critical canonical pathways in patients with T2DM and periodontitis compared with the patients with T2DM and without periodontitis.

Upregulated				Downregulated			
Gene symbol	Gene name	Q value	Log2 FC	Gene symbol	Gene name	Q value	Log2 FC
**Phagosome Formation: -log (p-value) = 4.75; z-score = 3.25**							
ABHD3	Abhydrolase Domain Containing 3, Phospholipase	8.31E-05	1.23	ADGRF4	Adhesion G Protein-Coupled Receptor F4	0.001502735	-1.34
ADGRD1	Adhesion G Protein-Coupled Receptor D1	0.000221199	1.49	ADGRG1	Adhesion G Protein-Coupled Receptor G1	7.43E-05	-1.14
ADGRE5	Adhesion G Protein-Coupled Receptor E5	0.000103337	1.01	AP1M2	Adaptor Related Protein Complex 1 Subunit Mu 2	0.01001205	-1.10
ADGRF5	Adhesion G Protein-Coupled Receptor F5	2.95E-10	1.47	CELSR1	Cadherin EGF LAG Seven-Pass G-Type Receptor 1	2.25E-05	-1.23
ADGRL4	Adhesion G Protein-Coupled Receptor L4	7.13E-08	1.69	CCR7	C-C Motif Chemokine Receptor 7	0.020276507	-1.20
AP1S2	Adaptor Related Protein Complex 1 Subunit Sigma 2	1.46E-06	1.35	F2RL1	F2R Like Trypsin Receptor 1	0.000861457	-2.03
APBB1IP	Amyloid Beta Precursor Protein Binding Family B Member 1 Interacting Protein	2.58E-05	1.41	LGR4:	Leucine Rich Repeat Containing G Protein-Coupled Receptor 4	3.95E-05	-1.08
APLNR	Apelin Receptor	1.22E-05	1.43	MAPK15	Mitogen-Activated Protein Kinase 15	0.010420729	-1.52
AVPR1A	Arginine Vasopressin Receptor 1A	0.000286265	1.55	MAPK7	Mitogen-Activated Protein Kinase 7	6.82E-05	-1.17
C3	Complement C3	0.000197404	1.44	MCHR1	Melanin Concentrating Hormone Receptor 1	0.036117633	-1.69
C5AR1	Complement C5a Receptor 1	0.000391725	1.57	P2RY11	Purinergic Receptor P2Y11	0.015989797	-1.06
CALCRL	Calcitonin receptor-like receptor	9.96E-06	1.48	PIK3R2	Phosphoinositide-3-Kinase Regulatory Subunit 2	3.02E-07	-1.24

CCR1	C-C Motif Chemokine Receptor 1	0.006780284	1.15	P2RY2	Purinergic Receptor P2Y2	4.27E-07	-1.71
CCR2	C-C Motif Chemokine Receptor 2	0.000328093	1.40	PLA2G2F	Phospholipase A2 Group IIF	3.51E-05	-4.97
CLEC4E	C-Type Lectin Domain Family 4 Member E	0.005497415	1.83	PLA2G4B	Phospholipase A2 Group IVB	0.001044935	-1.68
CR1	Complement receptor 1	8.19E-05	1.73	PLD2	Phospholipase D2	0.000178173	-1.11
CR2	Complement C3d Receptor 2	0.035193615	1.61	SMO	Smoothened, Frizzled Class Receptor	2.48E-05	-1.38
EDNRB	Endothelin receptor type B	0.002427503	1.06	SRC	SRC Proto-Oncogene, Non-Receptor Tyrosine Kinase	3.84E-05	-1.08
F2RL2	Coagulation Factor II Thrombin Receptor Like 2	3.37E-05	1.47	VAV3	Vav Guanine Nucleotide Exchange Factor 3	0.000301819	-1.16
FCER1G	Fc Fragment of IgE Receptor Ig	0.02219775	1.01	VIPR1	Vasoactive Intestinal Peptide Receptor 1	0.01962491	-1.15
FCGR1A	Fc Fragment of IgG Receptor Ia	0.004828328	1.27				
FCGR1B	Fc Fragment of IgG Receptor Ib	0.008226445	1.73				
FCGR2C	Fc Fragment of IgG Receptor IIc (Gene/Pseudogene)	0.010786066	1.33				
FZD3	Frizzled Class Receptor 3	1.49E-07	1.38				
GPR162	G Protein-Coupled Receptor 162	0.023897665	1.15				
GPR182	G Protein-Coupled Receptor 182	0.00076005	1.70				
GPR180	G Protein-Coupled Receptor 180	0.00169883	1.06				
GPR22	G Protein-Coupled Receptor 22	0.008043412	1.10				
GPR34	G Protein-Coupled Receptor 34	0.001864425	1.29				
GPR4	G Protein-Coupled Receptor 4	0.02343798	1.00				
GPR52	G Protein-Coupled Receptor 52	0.008604626	1.24				
GPR65	G Protein-Coupled Receptor 65	0.001368858	1.60				
HRH2	Histamine Receptor H2	0.006393526	1.35				
ITGA1	Integrin Subunit Alpha 1	3.91E-05	1.21				
ITGA4	Integrin Subunit Alpha 4	0.003952353	1.05				

ITGAM	Integrin Subunit Alpha M	0.002551803	1.29				
ITGAX	Integrin Subunit Alpha X	0.007791008	1.07				
LBP	Lipopolysaccharide Binding Protein	0.011434944	2.43				
LYN	LYN Proto-Oncogene, Src Family Tyrosine Kinase	0.000863943	1.01				
MSR1	Macrophage Scavenger Receptor 1	0.001405248	1.24				
PLA2G4C	Phospholipase A2 Group IVC	0.004463955	1.23				
PTGFR	Prostaglandin F Receptor	0.00014384	1.41				
S1PR3	Sphingosine-1-Phosphate Receptor 3	0.002548839	1.33				
S1PR4	Sphingosine-1-Phosphate Receptor 4	0.000299743	1.45				
TLR2	Toll Like Receptor 2	0.00426382	1.01				
TLR4	Toll Like Receptor 4	5.63E-07	1.66				
TLR8	Toll Like Receptor 8	0.013887157	1.39				
WIPF1	WAS/WASL Interacting Protein Family Member 1	0.000146684	1.13				
**PI3K/AKT Signaling: -log (p-value) = 3.69; z-score = 2.11**							
BCL2A1	BCL2 Related Protein A1	0.001296469	1.66	CCND1	Cyclin D1	0.000369428	-1.00
CDKN1B	Cyclin Dependent Kinase Inhibitor 1B	3.28E-07	1.22	IKBKE	Inhibitor Of Nuclear Factor Kappa B Kinase Subunit Epsilon	4.78E-06	-1.19
FOXO1	Forkhead Box O1	1.69E-05	1.01	IL12RB2	Interleukin 12 Receptor Subunit Beta 2	0.000404413	-1.79
GDF15	Growth Differentiation Factor 15	5.80E-07	3.02	IL17RC	Interleukin 17 Receptor C	1.80E-05	-1.36
IL18RAP	Interleukin 18 Receptor Accessory Protein	0.00028751	2.10	IL17RD	Interleukin 17 Receptor D	0.000389797	-1.15
IL6R	Interleukin 6 Receptor	2.05E-08	1.30	IL20RA	Interleukin 20 Receptor Subunit Alpha	0.015230566	-1.06
ITGA1	Integrin Subunit Alpha 1	3.91E-05	1.21	IL20RB	Interleukin 20 Receptor Subunit Beta	0.000300872	-1.12
ITGA4	Integrin Subunit Alpha 4	0.003952353	1.05	IL22RA1	Interleukin 22 Receptor Subunit Alpha 1	5.43E-07	-2.26

ITGAM	Integrin Subunit Alpha M	0.002551803	1.29	IL4R	Interleukin 4 Receptor	6.36E-06	-1.14
ITGAX	Integrin Subunit Alpha X	0.007791008	1.07	IL9R	Interleukin 9 Receptor	0.00360178	-2.34
PTGS2	Prostaglandin-Endoperoxide Synthase 2	0.005655989	1.44	INPP5J	Inositol Polyphosphate-5-Phosphatase J	0.00134853	-1.78
				PIK3R2	Phosphoinositide-3-Kinase Regulatory Subunit 2	3.02E-07	-1.29
				PPM1J	Protein Phosphatase, Mg2+/Mn2+ Dependent 1J	7.81E-05	-2.40
				PPP2R2B	Protein Phosphatase 2 Regulatory Subunit Bbeta	0.006000897	-1.74
				PPP2R3A	Protein Phosphatase 2 Regulatory Subunit B"Alpha	1.06E-05	-1.02
**Cardiac Œ≤-adrenergic Signaling: -log (p-value) =2.90; z-score=3.0**							
AKAP12	A-Kinase Anchoring Protein 12	4.23E-06	1.37	CACNA2D 3	Calcium Voltage-Gated Channel Auxiliary Subunit Alpha2delta 3	3.29E-06	-1.85
PDE10A	Phosphodiesterase 10A	0.000450127	1.18	CACNB4	Calcium Voltage-Gated Channel Auxiliary Subunit Beta 4	0.001979528	-1.39
PDE1A	Phosphodiesterase 1A	2.77E-06	2.26	GDPD3	Glycerophosphodiester Phosphodiesterase Domain Containing 3	0.005953332	-1.33
PDE4A	Phosphodiesterase 4A	0.000197404	1.05	GNA11	G Protein Subunit Alpha 11	9.91E-07	-1.15
PDE4B	Phosphodiesterase 4B	3.64E-05	1.30	PDE6A	Phosphodiesterase 6A	0.032414648	-1.44
PDE4D	Phosphodiesterase 4D	0.000401816	1.02	PPM1J	Protein Phosphatase, Mg2+/Mn2+ Dependent 1J	7.81E-05	-2.40
PDE5A	Phosphodiesterase 5A	2.69E-05	1.05	PPP1R14B	Protein Phosphatase 1 Regulatory Inhibitor Subunit 14B	1.36E-05	-1.12
PDE7B	Phosphodiesterase 7B	0.002560175	1.27	PPP1R14C	Protein Phosphatase 1 Regulatory Inhibitor Subunit 14C	0.000537905	-1.43
PRKACB	Protein Kinase CAMP-Activated Catalytic Subunit Beta	1.02E-06	1.50	PPP1R3C	Protein Phosphatase 1 Regulatory Subunit 3C	2.72E-05	-1.48

				PPP2R2B	Protein Phosphatase 2 Regulatory Subunit B beta:	0.006000897	-1.74
				PPP2R3A	Protein Phosphatase 2 Regulatory Subunit B"Alpha:	1.06E-05	-1.02
				PRKAR1B	Protein Kinase CAMP-Dependent Type I Regulatory Subunit Beta	0.005500148	-1.03
**tRNA Splicing: -log (p-value) = 2.83; z-score = 2.12**							
PDE10A	Phosphodiesterase 10A	0.000450127	1.18	GDPD3	Glycerophosphodiester Phosphodiesterase Domain Containing 3	0.005953332	-1.33
PDE1A	Phosphodiesterase 1A	2.77E-06	2.26	PDE6A	Phosphodiesterase 6A	0.032414648	-1.44
PDE4A	Phosphodiesterase 4A	0.000197404	1.05				
PDE4B	Phosphodiesterase 4B	3.64E-05	1.30				
PDE4D	Phosphodiesterase 4D	0.000401816	1.02				
PDE5A	Phosphodiesterase 5A	2.69E-05	1.05				
PDE7B	Phosphodiesterase 7B	0.002560175	1.27				
**Adrenomedullin signaling pathway: -log (p-value) = 3.80; z-score = -2.2**							
BCL2	BCL2 Apoptosis Regulator	3.64E-05	1.16	GNA11	G Protein Subunit Alpha 11	9.91E-07	-1.15
C3	Complement C3	0.000197404	1.44	GUCY2C	Guanylate Cyclase 2C	0.006407274	-1.22
CALCRL	Calcitonin receptor-like receptor	9.96E-06	1.48	IL1RN	Interleukin 1 Receptor Antagonist	0.029831597	-1.01
CASP3	Caspase 3	4.78E-07	1.22	IL36A	Interleukin 36 Alpha	0.002517721	-1.52
GPR182	G Protein-Coupled Receptor 182	0.000760052	1.70	MAPK13	Mitogen-Activated Protein Kinase 13	5.38E-07	-1.38
IL1A	Interleukin 1 Alpha	9.31E-05	1.22	MAPK15	Mitogen-Activated Protein Kinase 15	0.010420729	-1.52
PLCL2	Phospholipase C Like 2	2.00E-07	1.42	MAPK7	Mitogen-Activated Protein Kinase 7	6.82E-05	-1.17

PRKACB	Protein Kinase CAMP-Activated Catalytic Subunit Beta	1.02E-06	1.50	PIK3R2	Phosphoinositide-3-Kinase Regulatory Subunit 2	3.02E-07	-1.24
RAMP2	Receptor Activity Modifying Protein 2	4.27E-05	1.35	PLCD1	Phospholipase C Delta 1	3.66E-05	-1.38
				PLCD3	Phospholipase C Delta 3	0.000111424	-1.22
				PLCH2	Phospholipase C Eta 2	2.34E-08	-1.75
				PPARG	Peroxisome Proliferator Activated Receptor Gamma	0.033030069	-1.31
				PRKAR1B	Protein Kinase CAMP-Dependent Type I Regulatory Subunit Beta	0.005500148	-1.03
				RXRA	Retinoid X receptor alpha	9.18E-05	-1.08
				TFAP2A	Transcription Factor AP-2 Alpha	7.30E-08	-1.73
				TFAP2C	Transcription factor AP-2 gamma	8.19E-08	-1.37
**Superpathway of Cholesterol Biosynthesis: -log (p-value) = 3.68; z-score = -2.82**							
				ACAT2	Acetyl-CoA Acetyltransferase 2	2.67E-06	-1.61
				DHCR24	24-Dehydrocholesterol Reductase	0.00019843	-1.44
				DHCR7	7-Dehydrocholesterol Reductase	0.00073273	-1.04
				MVD	Mevalonate Diphosphate Decarboxylase	8.06E-06	-1.53
				MVK	Mevalonate Kinase	2.19E-05	-1.47
				PMVK	Phosphomevalonate kinase	3.19E-06	-1.10
				SQLE	Squalene Epoxidase	2.76E-05	-1.28
				TM7SF2	Transmembrane 7 Superfamily Member 2	5.64E-06	-1.48
**Superpathway of Inositol Phosphate Compounds: -log (p-value) = 2.73; z-score = -2.13**							
ALPL	Alkaline Phosphatase, Biomineralization Associated	2.25E-08	1.49	DUSP14	Dual Specificity Phosphatase 14	0.004823952	-1.20

EYA4	EYA Transcriptional Coactivator And Phosphatase 4	0.003920438	1.25	EPHX2	Epoxide Hydrolase 2	0.001017046	-1.06
NUDT4	Nudix Hydrolase 4	7.46E-07	1.12	INPP5J	Inositol Polyphosphate-5-Phosphatase J	0.00134853	-1.78
PPP1R16B	Protein Phosphatase 1 Regulatory Subunit 16B	0.001087609	1.02	IPPK	Inositol-Pentakisphosphate 2-Kinase	2.59E-06	-1.37
PTPN2	Protein Tyrosine Phosphatase Non-Receptor Type 2	8.70E-06	1.03	ITPKC	Inositol-Trisphosphate 3-Kinase C	0.001218582	-1.25
				NUDT1	Nudix Hydrolase 1	0.003150706	-1.09
				PIK3R2	Phosphoinositide-3-Kinase Regulatory Subunit 2	3.02E-07	-1.24
				PLCD1	Phospholipase C Delta 1	3.66E-05	-1.38
				PLCD3	Phospholipase C Delta 3	0.000111424	-1.22
				PLCH2	Phospholipase C Eta 2	2.34E-08	-1.75
				PPFIA3	PTPRF interacting protein alpha 3	0.000150788	-1.36
				PPP1R14B	Protein Phosphatase 1 Regulatory Inhibitor Subunit 14B	1.36E-05	-1.12
				PPP1R14C	Protein Phosphatase 1 Regulatory Inhibitor Subunit 14C	0.000537905	-1.43
				PPP1R3C	Protein Phosphatase 1 Regulatory Subunit 3C	2.72E-05	-1.47
				PPP2R2B	Protein Phosphatase 2 Regulatory Subunit B beta	0.006000897	-1.74
				PPP2R3A	Protein Phosphatase 2 Regulatory Subunit B"Alpha	1.06E-05	-1.02
				PTPN13	Protein Tyrosine Phosphatase Non-Receptor Type 13	0.00191405	-1.24
				SGPP2	Sphingosine-1-Phosphate Phosphatase 2	0.000186653	-1.26
				SSH3	Slingshot Protein Phosphatase 3	1.47E-07	-1.45
				PPM1J	Protein Phosphatase, Mg2+/Mn2+ Dependent 1J	7.81E-05	-2.40

179

Validation of transcriptome data by immunohistochemistry (IHC)

The immunohistochemistry (IHC) are shown in Figs. 3 and 4.

Protein tyrosine phosphatase non-receptor (PTPN) 2 showed slightly decreased expression in the DH group compared with the HH group. PTPN2 expression was increased in both periodontitis groups but was more pronounced in the DP group, particularly in the connective tissue (Fig. 3). PTPN13 had a noticeable nuclear and cytoplasmatic protein expression reduction in the DP group (Fig. 3). DHCR24 presented with considerable expression in the cytoplasm and nucleus in the basal and supra-basal layers of epithelial and endothelial cells in the HH group. However, its expression was significantly reduced in the DH group, especially in the basal cell layer. The DP group showed the lowest expression levels of the DHCR24 protein in intensity and distribution (Fig. 3). The DH group presented noticeably lower Phosphoinositide-3-Kinase Regulatory Subunit 2 (PIK3R2) protein expression than the HH group. While the PIK3R2 levels decreased in the HP group (mainly in the basal cell layer), the DP group showed increased cytoplasmatic and nuclear PIK3R2 expression in the epithelium and connective tissues (Fig. 3).

Fig. 3. The immunohistochemical staining using antibodies against PTPN2, PTPN13, DHCR24 (Seladin), and PIK3R2 in representative gingival biopsies from the HH, HP, DH, and DP groups. Bar length = 50 µm, original magnification × 20. HH:

non-diabetic patients without periodontitis; HP: non-diabetic patients with periodontitis; DH: T2DM patients without periodontitis; DP: T2DM patients with periodontitis.

The calcitonin receptor-like receptor (CALCRL) protein expression was overall stronger in diseased tissues than in healthy tissues (particularly in epithelial and endothelial cells). However, it was even stronger in the DP group, with nuclear and cytoplasmatic distribution. Notably, while CALCRL expression was clearly noticed in the basal cell layers in the HH group, it was barely expressed in the basal layers in the DH group. IL1RN showed intense staining in the cytoplasm and nucleus of all the epithelial cell layers in both periodontally-healthy groups; however, it had an even stronger staining in the DH group (Fig. 4). The IL1RN protein expression was considerably reduced in the cytoplasm and was more prominent in the nucleus of epithelial cells in the diseased tissues, especially in the DP group (Fig. 4). IL6R expression was overall stronger in the periodontitis groups than the non-periodontitis groups; however, it was even more intense in the DP group, particularly in the basal layer of the epithelium and connective tissue (Fig. 4). There was a robust ITGA4 expression in the cytoplasm and the membrane of supra-basal cell layers of the epithelium in the HH, HP, and DH groups. Remarkably, the basal cell layer in the DH group exhibited no or mild ITGA4 protein expression. Moreover, the DP group presented a more noticeable ITGA4 expression even in the basal layers of the epithelium and underlying connective tissue cells (Fig. 4).

Fig. 4. The immunohistochemical staining using antibodies against CALCRL, IL1RN, IL6R, and ITGA4 in representative gingival biopsies from the HH, HP, DH, and DP groups. Bar length = 50 μm, original magnification × 20. HH: non-diabetic patients without periodontitis; HP: non-diabetic patients with periodontitis; DH: T2DM patients without periodontitis; DP: T2DM patients with periodontitis.

Discussion

We show here that periodontitis in non-diabetic and diabetic patients is a distinct process at the whole transcript level. T2DM significantly alters the expression of specific genes and signaling pathways involved in immune and inflammatory responses that may affect the course of periodontitis.

Some of the most upregulated and downregulated genes are worthy of attention. CXCL5 was among the top 15 upregulated DEGs, while LCE2 and LCE3 were among the top 15 downregulated DEGs observed in T2DM-related periodontitis. CXCL5 is a chemokine ligand of the IL-8 receptor type 2 that has proinflammatory activities, including chemotaxis and angiogenesis [7,8]. In support of our findings, previous studies observed an association between increased CXCL5 levels and DM complications [8,9]. Noteworthy, lipopolysaccharide (LPS) from *Porphyromonas gingivalis,* in combination with advanced glycation end products (AGE), induced greater CXCL5 expressions from monocyte and oral keratinocyte cells [10]. Although the biological functions of LCE are

not entirely understood, they seem to have antibacterial activities and a role in the epithelial physical barrier [11,12]. The lack of LCE3B/C causes dysbiotic changes in the oral and cutaneous microbiota in patients with psoriasis [12]. Other possibly relevant downregulated genes to the pathogenesis of T2DM-related periodontitis are keratin 76, critical for epidermal integrity [13], and the PLA2G2F, as PLA2s are involved in host defense and barrier function [14].

The IPA of the DEGs that were exclusively expressed in periodontitis in diabetic patients revealed a significant upregulation of the Phagosome Formation pathway through the alterations in the expression of genes required for actin cytoskeleton rearrangements, membrane ruffle formation, and phagosome closure (e.g., complement, complement receptor (CR), ITGs, GPCRs, scavenger receptor, toll-like receptor, LPS binding protein, Fc receptor, C-type lectin domain family 4 member E, chemokine receptor, and PL) [15]. Noteworthily, some of the phagosome formation-associated genes that were upregulated in T2DM-related periodontitis (e.g., C3a, C5ar1, and LYN) were also found to be upregulated in other DM complications [16,17], functioning against microbial invasion and as proinflammatory factors and regulators of immunometabolism [17].

Despite the significant activation of the Phagosome Formation pathway in T2DM-related periodontitis, some critical pathways in response to infections may be impaired in diabetic hosts, including the completion of phagocytosis [18,19]. Hyperglycemia/DM can affect the expression of complement-related factors required for bacterial opsonization

and internalization [18], and reduce the phagosome maturation which succeeds phagosome formation [19]. Finally, the dysregulation of PI3Ks, herein represented by PIK3R2, may also affect phagocytosis [20]. Thus, despite the effort to recruit immune cells and form phagosomes in disease tissues in patients with T2DM, under hyperglycemia, microorganisms may evade phagosome maturation and degradation in the phagolysosomes, leading to persistent infection and inflammation. The impact of DM on different stages of phagocytosis represents an interesting area for further research in the context of periodontitis.

DEGs uniquely expressed in T2DM-related periodontitis were related to the activation of the PI3K/AKT signaling pathway that is activated through various stimuli (e.g., cytokines, growth factors, cellular stresses, and insulin) and regulates several biological processes (e.g., cellular survival, apoptosis, autophagy, proliferation, growth, motility, transcription, and translation) involved in periodontitis and other diseases [21,22]. Additionally, the PI3K/AKT pathway is reported to regulate insulin and hyperglycemia, and the insulin resistance [21] and the AGE-RAGE axis can activate PI3K/AKT signaling [23]. Finally, the activation of the PI3K/AKT signaling pathway has been associated with DM-related complications [24] and with osteoclastogenesis and bone resorption, an important hallmark of periodontitis [25].

The relevant genes that were involved in the PI3K/AKT pathway activation observed in the T2DM-related periodontitis included those that affect apoptosis (e.g., FOXO1, BCL2,

and growth differentiation factor 15 [GDF15]) and cell cycle passage (e.g., CDKN1B and CCND1). Also abundant were the dysregulated expression of pro- and anti-inflammatory cytokine receptors (e.g., IL18RAP, IL6R, IL12RB2, IL17RC, IL17RD, IL20RA, IL20RB, IL22RA1, IL4R, and IL9R), supporting the notion that cytokines play pivotal roles in the pathogenesis of T2DM-related periodontitis, a typical infectious-inflammatory disorder. The concurrent increased expression of IL-6R at the protein level observed in diseased tissues in diabetic patients indicates the role of the pro-inflammatory IL-6/IL-6R axis in disease pathogenesis. ITGs, which mediate cell-matrix interactions and leukocyte migration and adhesion [26], were consistently overexpressed in T2DM-related periodontitis, and the ITGA4 upregulation was confirmed at the protein level. T2DM-related periodontitis also exhibited marked upregulation of GDF15 (also known as macrophage inhibitory cytokine-1) (Table 1), which was reported to play a role in regulating inflammatory and apoptotic pathways and whose expression was found to be significantly higher in multiple tissues of diabetic patients [27]. Finally, the downregulation of kinases and phosphatase-related genes in diseased tissues in diabetic patients is consistent with the role of the PI3K/AKT signaling as a classical phosphorylation cascade, where the protein kinases regulate phosphorylation and the phosphatases (e.g., PPP family) control dephosphorylation reactions. Remarkably, PIK3R2, a PI3K subunit involved in multiple canonical pathways, was downregulated at the mRNA level but was upregulated at the protein level in T2DM-related periodontitis. This downregulation could be due to the low stability of mRNA and/or the reduced

proteasomal degradation of PIK3R2 since PTPN13, which dephosphorylates PIK3R2 at Tyr-655 [28], was decreased at the mRNA and protein levels. However, further studies are needed to confirm this assumption.

The IPA also predicted the activation of the Cardiac β-adrenergic and tRNA Splicing pathways in the T2DM-related periodontitis, which were predominantly driven by the upregulation of phosphodiesterases (PDE 1, 4, 5, 7, and 10), a superfamily of cAMP/cGMP hydrolyzing enzymes. The upregulation of PDEs has been linked to the cellular decrease of cAMP and cGMP and to the increase of oxidative stress and pro-inflammatory factors. Hence, the dysregulation of PDEs has been associated with inflammation, DM, DM complications, and imbalanced bone homeostasis [29] whereas the inhibition of PDEs has been suggested as a strategy for treating infectious and inflammatory conditions [29-31]. Thus, the use of PDE inhibitors as an add-on treatment for T2DM-related periodontitis seems to be an attractive research topic.

The inactivation of the ADM signaling pathway was predicted in T2DM-related periodontitis. The downregulated genes associated with the ADM pathway included MAPKs, PLCs, the PIK3R2, the guanylate cyclase C, implicated in infection and inflammation control and the IL1RN, which encodes the anti-inflammatory protein IL-1Ra. Worthy of note, IL1RN mRNA downregulation was validated at the protein level by IHC. The ADM signaling pathway was also related to the upregulation of BCL2 and caspase-3, regulators of apoptosis, C3, an activator of the complement system, and IL1A,

a pro-inflammatory mediator. Importantly, ADM has antimicrobial properties in the oral mucosal epithelia [32] and acts as an angiogenic, anti-apoptotic, and anti-inflammatory factor through the G protein-coupled receptor 182 (GPR182) and the receptor activity modifying protein 2 (RAMP2)/CALCRL complex [33]. While ADM2 was upregulated in periodontitis in the diabetic and non-diabetic patients (table S4), GPR182, CALCRL (validated at the protein level), and RAMP2 were significantly upregulated in the diabetic patients only, suggesting that ADM may perform its biological functions mainly in T2DM-related periodontitis. This hypothesis needs to be tested in further studies.

The superpathway of Cholesterol Biosynthesis and some of its related DEGs were downregulated in the T2DM-related periodontitis, suggesting the existence of a transcriptional regulation connecting this metabolic pathway with the immune system. DM and insulin deficiency have been reported to suppress the production of cholesterol synthesis enzymes and the transcriptional regulator sterol regulatory element-binding protein-2 (SREBF2; here, downregulated in the diabetic patients only; table S3) [34]. Overall, the reduction of cholesterol at a cellular level and the dysregulation of its precursors might affect cell proliferation, cycle progression, apoptosis, inflammation, and phagocytosis [35]. Notably, cholesterol intermediates may play biological functions besides cholesterol biosynthesis, which may affect inflammation and immunity [36]. Dehydrocholesterol reductase (DHCR) 7, for example, is involved in vitamin D synthesis, macrophage polarization, and regulation of IL-10 expression. Transmembrane

7 superfamily member 2 (TM7SF2) acts on the cell cycle, differentiation, and apoptosis and directs an anti-inflammatory loop [35-36]. DHCR24, here, downregulated at the mRNA and protein levels in the T2DM-related periodontitis, has antiapoptotic functions and inhibits the generation of intracellular reactive oxygen species [35-36] and LPS-induced osteoclastogenesis [37]. Future in-depth studies are required to identify the actual role of cholesterol biosynthesis-related genes on the pathogenesis of T2DM-related periodontitis.

The inhibition of SIPC was predicted in the T2DM-related periodontitis. Inositol phosphates are membrane components that have functions in cell growth, differentiation, migration, apoptosis, and endocytosis. Inositols are considered possible mediators of insulin signaling, and their insufficiency may contribute to insulin resistance, T2DM, and DM complications [38]. Among the proteins encoded by the downregulated DEGs associated with SIPC observed in the T2DM-related periodontitis were predominantly kinases and phosphatases, which not only control phosphorylation but also have further biological functions that can affect periodontitis progression. Dual-specificity phosphatase 14 (DUSP14) and inositol 1,4,5-trisphosphate 3-kinase C (ITPKC), for example, are both negative regulators of T-cell activation [39]. DUSP14 also has inhibitory effects on inflammation and osteoclastogenesis [37]. PTPN13, downregulated in diseased tissues in the diabetic patients at the mRNA and protein levels, is involved in apoptosis, cell migration, epithelial cell junction stabilization, and epithelial barrier

regulation [40]. PTPN2, a vitamin-D-responsive tyrosine-specific phosphatase, acts as a protection factor controlling glucose homeostasis and pro-inflammatory conditions [40]. Therefore, the transcript-level and protein-level overexpression of PTPN2 observed in this study may represent an attempt to control the periodontal breakdown in the diabetic patients. Lastly, the observed downregulation of PLCs may also have implications for the pathogenesis of T2DM-related periodontitis. For instance, the lack or reduction of PLCD1 and PLCD3 was associated with increased cell apoptosis and the upregulation of LPS-induced proinflammation [41].

The IPA on the DEGs in the non-diabetic patients revealed less of an impact on the canonical pathways (there were no pathways with a Z- score >2 or <−2). Among the dysregulated genes associated with the Tumor Microenvironment pathway observed in the non-diabetic patients were those that encoded MMPs, which play well-known roles in inflammation and immunity [42]. Importantly, ATF3, FOS, and JUN, that function in Tumor Microenvironment and PI3K Signaling in B Lymphocytes pathways, were exclusively downregulated in periodontitis in the non-diabetic patients. JUN, FOS, ATF, and FOSB (among the top ten downregulated genes in the non-diabetic patients; Table 1) are members of the activator protein-1 (AP-1) transcription factor complex, which is involved in cell proliferation, migration, apoptosis, inflammation, and bone remodeling [43]. The analysis of the transcriptomic datasets of human periodontitis (GSE16134 and GSE10334) and the immunosuppression genes by deep learning-based autoencoder

techniques suggested that FOS and JUN were downregulated transcription factors with possible roles in periodontitis [44]. Using the same transcriptomic database (GESE16134), FOSB was identified as an upregulated DEG with potential participation in periodontitis [45]. Future studies are needed to explore the actual involvement of the AP-1 complex-related genes in periodontitis.

Long non-coding RNAs (lncRNAs) may have coding potential and affect the transcription, post-transcription, and translation of other genes and the activation or suppression of various encoding molecules and signaling pathways [46-47]. In this study, tissues with periodontitis in diabetic and non-diabetic patients exhibited dysregulation in the expression of several lncRNAs, supporting the findings of previous investigations [48-49]. lncRNAs were also suggested to play critical roles in DM complications [50-51]. Intriguingly, two lncRNAs ranked in the top 15 downregulated DEGs (SCARNA2 and SMAD1-AS1) were only expressed in the healthy tissues in diabetic patients (Table 1). SCARNA2 has been reported to participate in DNA repair [52], whereas the biological functions of SMAD1-AS1 are still unknown. To understand the roles of lncRNAs in the pathogenesis of T2DM-related periodontitis is a future challenge.

A strength of this study is to be the first RNA-seq experiment evaluating the exclusively DEGs in T2DM-related periodontitis. Moreover, data were expanded by using IPA to distinguish the associated signaling pathways. A possible limitation is the small sample size. However, a previous study indorsed a minimum of five samples per group for RNA-

Seq datasets [53]. Another limitation is the analysis of the entire gingival transcriptomic profiles from a mix of cells that tends to reflect the gene expression patterns of predominant cell types (e.g., epithelial cells and fibroblasts). Thus, additional studies are needed to elucidate individual cell transcription profile involved in T2DM-related periodontitis. Also, the cross-sectional nature of this study does not allow for causal and temporal inferences on the effects of a given gene/pathway on disease progression.

Conclusion

Periodontitis in non-diabetic and T2DM patients are distinct disease events at the transcript level. T2DM-related periodontitis has unique transcriptomic signatures, which lead to the dysregulation of functional pathways that may negatively affect periodontal tissues. These findings provide new insights into the pathogenesis of T2DM-related periodontitis and reveal pathways and genes that could be targeted for diagnosis, preventive and therapeutic approaches. Moreover, a valuable database for the future functional validation of DEGs and signaling pathways was generated.

Material and Methods

Population

In this cross-sectional study, 36 patients (45-85 years old) were selected from a pool of patients seeking treatment at the University of Florida’s Dental Clinics from January 2020 to January 2022. The study was approved by the Institutional Review Board (IRB201901176), and written informed consent was obtained from all eligible

individuals. All experiments were performed in accordance with the Declaration of Helsinki (Ethical Principles for Medical Research Involving Human Subjects) and relevant guidelines and regulations.

Patients were assigned into one of the following four groups: non-diabetic patients without periodontitis (HH; n=9), non-diabetic patients with periodontitis (HP; n=9), T2DM patients without periodontitis (DH; n=9), and T2DM patients with periodontitis (DP: n=9). The periodontally-healthy patients had no history of periodontitis, <10% of sites with marginal bleeding (MB) or bleeding on probing (BoP), and probing depths (PD) ≤3mm [54]. Furthermore, the periodontally-healthy patients should have needed at least one crown lengthening surgery or tooth extraction due to non-periodontitis reasons. The periodontitis patients had localized or generalized stage III or IV grade B or C periodontitis [55] and a referral for at least one tooth extraction due to severe untreated periodontitis. The diabetic patients were required to have a diagnosis of T2DM confirmed by a physician, dating from at least the last three years, and confirmed by HbA1c levels ≥7% and a fasting plasma glucose (FPG) >99 mg/dl in last two months. The non-diabetic patients were required to have no history of pre-DM or DM, confirmed by an HbA1c <5.5 % and an FPG <99 mg/dl.

The exclusion criteria were history of smoking (current or past); pregnancy; lactation; use of antibiotics, anti-inflammatory, immunosuppressant, or other immune regulators within six months; use of bone metabolism-related medications (e.g., bisphosphonates, or

denosumab); subgengival periodontal treatment in the past 12 months; regular use of mouth rinses containing antimicrobials; and other systemic conditions that could affect periodontitis progression [56].

The clinical data of the participants were obtained from their records, including age, sex, HbA1C levels, duration of DM, and periodontal parameters.

Tissue collection

Gingival biopsies, including oral, junctional, and sulcular epitheliums, and connective tissue were obtained from the waste specimens of surgical procedures. In patients with periodontitis, the biopsies were obtained from sites with a PD and clinical attachment level (CAL) ≥5 mm and with BoP, mobility, and/or bone loss of more than half of the root. In the periodontally-healthy patients, the biopsies were obtained from teeth with a PD ≤3 mm, no bone loss, and no BoP or marginal bleeding. After removing the blood from the biopsies by washing them in a saline solution, samples were immediately stored in an RNA later solution (Ambion Inc., Austin, TX, USA) at −80oC.

RNA extraction

Total RNA was isolated from the biopsies using the Trizol method (TRIzol™ Reagent, Thermo Fisher Scientific, Waltham, MA, USA), according to the manufacturer's instructions. RNA samples were resuspended in diethylpyrocarbonate-treated (DEPC) water and stored at −80oC. The RNA concentration was determined by the optical density using a micro-volume spectrophotometer (Nanodrop 1000, Nanodrop

Technologies LLC, Wilmington, NC, USA). The total RNA was DNase treated (Turbo DNA-free, Ambion Inc., Austin, TX, USA) to rid the RNA samples of contaminating DNA.

Library construction, sequencing, and bioinformatics

Illumina Sequencing Library Construction

The RNA-seq library construction was performed at the University of Florida's ICBR Gene Expression and Genotyping Core Facility (RRID: SCR_019145). The RNA samples were measured by the QUBIT fluorescent method (Invitrogen, Waltham, MA, USA) and Agilent Bioanalyzer (Agilent, Santa Clara, CA, USA). An amount of 500 ng of protein-free, total RNA was used for the library construction using the reagents provided in the NEBNext rRNA depletion kit (catalog # E6350) and the Ultra Directional RNA Library Prep Kit for Illumina (New England Biolabs, Ipswich, MA, USA), following the manufacturer's recommendations. Two microliters of probe hybridization buffer were added to the total RNA samples, followed by RNase H and DNAase I digestion. Subsequently, the RNA was purified using NEBNext RNA purification beads (New England Biolabs, catalog # E6350). This step was followed by the RNA library construction using the NEBNext Ultra II Directional Lib Prep (New England Biolabs, catalog # E7760), according to the manufacturer's user guide. Briefly, RNA was fragmented in a solution containing divalent cations and incubated at 94oC, followed by first-strand cDNA synthesis using reverse transcriptase and oligo dT primers. The

synthesis of the ds-cDNA was done using the 2nd strand master mix provided in the kit, followed by end-repair and dA-tailing. Next, Illumina adaptors were ligated to the samples. Finally, the library was amplified, followed by purification with AMPure beads (Beckman Coulter, Pasadena, CA, USA, catalog # A63881). The library size and mass were assessed by analysis in an Agilent TapeStation using a DNA5000 Screen Tape (Agilent, Santa Clara, CA, USA). A 200–1000 broad library peak was observed, with the highest peak at around 500 bp. Quantitative PCR was used to validate the library's functionality, using the KAPA library quantification kit (Kapa Biosystems, Wilmington, MA, USA catalog # KK4824) and monitored on a BioRad CFX 96 real-time PCR system (Bio-Rad Laboratories, Hercules, CA, USA). Eighteen barcoded samples were pooled equimolarly for simultaneous sequencing, as described below.

Illumina NovaSeq6000 Sequencing

The sequencing run was performed at the University of Florida’s ICBR NextGen DNA Sequencing Core Facility (RRID: SCR_019152). Briefly, normalized libraries were submitted to the Illumina Free Adapter Blocking (FAB) Reagent protocol (Illumina, San Diego, CA, USA, catalog # 20024145) to minimize the presence of adaptor-dimers and index hopping rates. The library pool was diluted to 0.8 nM and sequenced on the one S4 flow cell lane (2×150 cycles) of an Illumina NovaSeq6000 (Illumina, San Diego, CA, USA). The instrument’s computer utilized NovaSeq Control Software v1.6. Cluster and SBS consumables were v1.5. The final loading concentration of the library was 120 pM

with a 1% PhiX spike-in control. One lane generated approximately 2.5 billion paired-end reads (~750 Gb) with an average Q30% greater or equal to 92.5% and a Cluster PF of 85.4%. FastQ files were generated using the BCL2fastQ function in the Illumina BaseSpace portal. An average of 75 million demultiplexed, paired-end reads were used for the data analyses.

Sequence quality control

The quality of the RNA-Seq sequence data was first evaluated using FastQC (FASQC) [57] prior to further downstream analysis. Low-quality sequences were trimmed, and poor-quality reads were removed using the Trimmomatic tool [58].

Differential gene expression analysis

Data analyses were performed at the University of Florida's ICBR Bioinformatics core. A Star Aligner [59] was used to map the high-quality single-end reads to the genome, GRCh38 (https://useast.ensembl.org/Homo_sapiens/Info/Index/). The gene expression was obtained using RSEM [60]. The expected read counts and fragments per kilobase of transcript per million mapped (FPKM) were extracted for further analysis. The estimated read counts were used as the input for edgeR [61] to perform the differential gene expression analysis. The exact test was developed to identify DEGs, and the thresholds were set at an FDR of 0.05 and a fold change of greater than or equal to 2. Pairwise comparisons were made between the healthy controls and periodontitis in the non-diabetic and diabetic patients (HP versus HH and DP versus DH). Prior to the DE

analysis, a PCA was performed to identify outlier samples. No obvious outlier samples were found.

Pathway analysis

An Ingenuity Pathway Analysis (IPA) (Qiagen, Redwood City, CA) was performed on the DEGs expressed exclusively in the diseased tissues in diabetic and non-diabetic patients to identify the canonical pathways dysregulated in periodontitis in the presence and absence of T2DM. The transcripts that were identified to be differently expressed in the tissues with periodontitis compared with the tissues with periodontal heath in non-diabetic (HP versus HH) and diabetic patients (DP versus DH), along with their respective fold changes, were input into the IPA, for subsequent bioinformatics analysis. The Canonical Pathways were used for interpreting the functions of the DEGs. For the Ingenuity Canonical Pathway analysis, a −log (P-value) greater than 2 was taken as the significant threshold for the diabetic and non-diabetic groups. A Z-score over 2 was defined as the threshold of significant activation, while a Z-score under −2 was defined as the threshold of significant inhibition for patients with T2DM. No significant signaling pathways in the non-diabetic patients presented a Z-score over 2 or under −2. Therefore, a Z-score under −1.3 was defined as the threshold of significant inhibition for the non-diabetic patients.

Clinical and demographic data analyses

The mean percentages of sites with plaque and BoP, the mean full-mouth PD and CAL, the mean PD and CAL of the sampled teeth, the HbA1c levels, and the duration of DM were computed for each subject and, subsequently, across the groups. Data were examined for normality by the Shapiro-Wilk test, and parametric methods were used as data achieved a normal distribution. The significance of differences for age, HbA1c, and clinical parameters was compared by a one-way ANOVA, followed by a Tukey test. A t-test was used to compare the duration of DM between both diabetic groups. The significance of differences for sex was compared by a Chi-square test. The level of significance for all these analyses was set at 5%.

IHC

IHC experiments on four tissues per group were performed in an independent set of patients to confirm the transcriptome findings at the protein level. The selection of the genes for validation was performed based on their biological relevance within the representative signaling pathways observed in the diseased tissues of patients with T2DM and the magnitude of difference in mRNA levels between healthy and disease tissues in diabetic and non-diabetic patients. Four micrometers-thick FFPE sections were set on positively charged glass slides. All the staining steps were performed on a Leica RX Autostainer (Leica Biosystems, Wetzlar, Germany), following the vendor’s instructions. The slides were de-paraffinized, re-hydrated, and heat-induced antigen retrieval treated with either epitope retrieval (ER)1 or ER2 buffers for 20 minutes. The slides were treated

with hydrogen peroxide to eliminate endogenous peroxidases for five minutes. Afterward, the sections were incubated with primary antibodies against PTPN2 (1:500, polyclonal, rabbit, PA582516, ThermoFisher Scientific, Waltham, MA, USA), PTPN13 (1:500, polyclonal, rabbit, 25944-1-AP, Proteintech, Rosemont, IL, USA), DHCR24 (1:750, polyclonal, rabbit, 10471-1-AP), PIK3R2 (1:100, monoclonal, mouse, 67644-1-Ig, Proteintech, Rosemont, IL, USA), CALCRL (1:400, polyclonal, rabbit, PA550644, ThermoFisher Scientific, Waltham, MA, USA), IL1RN (1:2000, monoclonal, mouse, CF803396, ThermoFisher Scientific, Waltham, MA, USA), IL-6R alpha (1:350, polyclonal, rabbit, 23457-1-AP, Proteintech, Rosemont, IL, USA), and ITGA4 (1:500, polyclonal, rabbit, 10471-1-AP, ThermoFisher Scientific, Waltham, MA, USA) for 30 minutes. The sections were then treated with an HRP Polymer (Leica Biosystems, Wetzlar, Germany) for eight minutes. The specific reaction for each antibody was observed through the application of 3,3'diaminobenzidine (DAB) for ten minutes. The sections were counter-stained with hematoxylin (Leica Biosystems, Wetzlar, Germany), dehydrated through graded ethanol, cleared in xylene, and mounted on slides with the aid of Cytoseal mounting media (Richard-Allan Scientific, San Diego, CA, USA). The negative controls were obtained by the omission of the primary antibodies. Results are presented in a descriptive form, including the description of the histological picture regarding which cells or tissue components were immunopositive, patterns of staining (membrane, cytoplasm, or nuclear), and intensity of IHC expression (weak, moderate, or strong).

Acknowledgments: All funding was provided by University of Florida College of Dentistry Faculty Seed Grant. We thank Dr. Ann Fu Dongtao for her technical assistance with immunohistochemistry.

Author contributions: PMD and IA contributed to the study conceptualization, funding acquisition and project administration. PMD, BCVG, TSM and JS contributed to the sample collections and IHC analyses. PMD, BCVG and IA contributed to the RNA-seq. IG, PMD, BCVG and IA contributed to data analyses and interpretation. PMD, BCVG and IA contributed to the original draft. All authors contributed to manuscript review and editing.

Data and materials availability: All data are available in the main text or the supplementary materials.

Ethics declaration

Competing interests: Authors declare that they have no competing interests.

Human experimentation: The study was approved by the University of Florida's Institutional Review Board (IRB201901176)

References

1. Emrich LJ, Shlossman M, Genco RJ. Periodontal disease in non-insulin-dependent diabetes mellitus. J Periodontol. 1991;62: 123-131.

2. Graziani F, Gennai S, Solini A, Petrini M. A systematic review and meta-analysis of epidemiologic observational evidence on the effect of periodontitis on diabetes An update of the EFP-AAP review. J Clin Periodontol. 2018;45: 167-187.

3. Tonetti MS, Greenwell H, Kornman KS. Staging and grading of periodontitis: Framework and proposal of a new classification and case definition. J Clin Periodontol. 2018; 45 Suppl 20: S149-S161.

4. Sonnenschein SK, Meyle J. Local inflammatory reactions in patients with diabetes and periodontitis. Periodontol 2000. 2015;69: 221-254.

5. Graves DT, Ding Z, Yang Y. The impact of diabetes on periodontal diseases. Periodontol 2000. 2020;82: 214-224.

6. Park SJ, Saito-Adachi M, Komiyama Y, Nakai K. Advances, practice, and clinical perspectives in high-throughput sequencing. Oral Dis. 2016;22: 353-364.

7. Koch AE. Chemokines and their receptors in rheumatoid arthritis: future targets? Arthritis Rheum. 2005;52:710-721.

8. Chavey C, Fajas L. CXCL5 drives obesity to diabetes, and further. Aging (Albany NY). 2009;1: 674-677.

9. Zychowska M, Rojewska E, Pilat D, Mika J. The role of some chemokines from the CXC subfamily in a mouse model of diabetic neuropathy. J Diabetes Res. 2015;2015: 750182.

10. Lappin DF, Robertson D, Hodge P, Treagus D, Awang RA, Ramage G, et al. The Influence of Glycated Hemoglobin on the Cross Susceptibility Between Type 1 Diabetes Mellitus and Periodontal Disease. J Periodontol. 2015;86:1249-1259.

11. de Koning HD, van den Bogaard EH, Bergboer JG, Kamsteeg M, van Vlijmen-Willems IM, Hitomi K, et al. Expression profile of cornified envelope structural proteins and keratinocyte differentiation-regulating proteins during skin barrier repair. Br J Dermatol. 2012; 166: 1245-1254.

12. Niehues H, van der Krieken DA, Ederveen THA, Jansen PAM, van Niftrik L, Mesman R, et al. Antimicrobial Late Cornified Envelope Proteins: The Psoriasis Risk Factor Deletion of LCE3B/C Genes Affects Microbiota Composition. J Invest Dermatol. 2022;142: 1947-1955.e6.

13. DiTommaso T, Cottle DL, Pearson HB, Schlüter H, Kaur P, Humbert PO, et al. Keratin 76 is required for tight junction function and maintenance of the skin barrier. PLoS Genet. 2014;10: e1004706.

14. Shao S, Chen J, Swindell WR, Tsoi LC, Xing X, Ma F, et al. Phospholipase A2 enzymes represent a shared pathogenic pathway in psoriasis and pityriasis rubra pilaris. JCI Insight. 2021;6: e151911.

15. Lee HJ, Woo Y, Hahn TW, Jung YM, Jung YJ. Formation and Maturation of the Phagosome: A Key Mechanism in Innate Immunity against Intracellular Bacterial Infection. Microorganisms. 2020;8: 1298.

16. Tang S, Wang X, Deng T, Ge H, Xiao X. Identification of C3 as a therapeutic target for diabetic nephropathy by bioinformatics analysis. Sci Rep. 2020 10(1): 13468.

17. Tan SM, Snelson M, Østergaard JA, Coughlan MT. The Complement Pathway: New Insights into Immunometabolic Signaling in Diabetic Kidney Disease. Antioxid Redox Signal. 2022;37:781-801.

18. Hodgson K, Morris J, Bridson T, Govan B, Rush C, Ketheesan N. Immunological mechanisms contributing to the double burden of diabetes and intracellular bacterial infections. Immunology. 2015;144: 171-185.

19. Dowey R, Iqbal A, Heller SR, Sabroe I, Prince LR. A Bittersweet Response to Infection in Diabetes; Targeting Neutrophils to Modify Inflammation and Improve Host Immunity. Front Immunol. 2021;12: 678771.

20. Gillooly DJ, Simonsen A, Stenmark H. Phosphoinositides and phagocytosis. J Cell Biol. 2001;155: 15-17.

21. Huang X, Liu G, Guo J, Su Z. The PI3K/AKT pathway in obesity and type 2 diabetes. Int J Biol Sci. 2018;14:1483-1496.

22. Kumar M, Bansal N. Implications of Phosphoinositide 3-Kinase-Akt (PI3K-Akt) Pathway in the Pathogenesis of Alzheimer's Disease. Mol Neurobiol. 2022;59: 354-385.

23. Dariya B, Nagaraju GP. Advanced glycation end products in diabetes, cancer and phytochemical therapy. Drug Discov Today. 2020;25: 1614-1623.

24. Xing X, Jiang Y, Wang H, Zhang Y, Niu T, Qu Y, et al. Identification of novel differentially expressed genes in retinas of STZ-induced long-term diabetic rats through RNA sequencing. Mol Genet Genomic Med. 2020;8: e1115.

25. Leibbrandt A, Penninger JM. RANK/RANKL: regulators of immune responses and bone physiology. Ann N Y Acad Sci. 2008;1143: 123-150.

26. Mezu-Ndubuisi OJ, Maheshwari A. The role of integrins in inflammation and angiogenesis. Pediatr Res. 2021;89: 1619-1626.

27. Lu J, Zhang Y, Dong X, Lu J, Zhang C, Liu J, et al. Association between MIC-1 and Type 2 Diabetes: A Combined Analysis. Dis Markers. 2019;2019: 7284691.

28. Kuchay S, Duan S, Schenkein E, Peschiaroli A, Saraf A, Florens L, et al. FBXL2- and PTPL1-mediated degradation of p110-free p85β regulatory subunit controls the PI(3)K signalling cascade. Nat Cell Biol. 2013;15:472-480.

29. Kilanowska A, Ziółkowska A. Role of Phosphodiesterase in the Biology and Pathology of Diabetes. Int J Mol Sci. 2020;21: 8244.

30. Su H, Ma C, Li H. Anti-infection mechanism of phosphodiesterase-5 inhibitors and their roles in coronavirus disease 2019 (Review). Exp Ther Med. 2021 Apr;21(4):320.

31. Ookawara M, Nio Y. Phosphodiesterase 4 inhibitors in diabetic nephropathy. Cell Signal. 2022;90: 110185.

32. Hinson JP, Kapas S, Smith DM. Adrenomedullin, a multifunctional regulatory peptide. Endocr Rev. 2000;21: 138-167

33. Xu M, Qi F, Zhang S, Ma X, Wang S, Wang C, Fu Y, Luo Y. Adrenomedullin promotes the growth of pancreatic ductal adenocarcinoma through recruitment of myelomonocytic cells. Oncotarget. 2016;7: 55043-55056.

34. Suzuki R, Lee K, Jing E, Biddinger SB, McDonald JG, Montine TJ, et al. Diabetes and insulin in regulation of brain cholesterol metabolism. Cell Metab. 2010;12: 567-579.

35. Bah SY, Dickinson P, Forster T, Kampmann B, Ghazal P. Immune oxysterols: Role in mycobacterial infection and inflammation. J Steroid Biochem Mol Biol. 2017;169: 152-163.

36. Brown AJ, Ikonen E, Olkkonen VM. Cholesterol precursors: more than mere markers of biosynthesis. Curr Opin Lipidol. 2014; 25:133-139.

37. Khuda II, Koide N, Noman AS, Dagvadorj J, Tumurkhuu G, Naiki Y, et al. Seladin-1 is a novel lipopolysaccharide (LPS)-responsive gene and inhibits the tumour necrosis factor-alpha production and osteoclast formation in response to LPS. Immunology. 2010;131:59-66.

38. Caputo M, Bona E, Leone I, Samà MT, Nuzzo A, Ferrero A, et al. Inositols and metabolic disorders: From farm to bedside. J Tradit Complement Med. 2020;10: 252-259.

39. Zhao Z, Yang J, Zhang L, Zhou Y. Enhancement of DUSP14 (dual specificity phosphatase 14) limits osteoarthritis progression by alleviating chondrocyte injury, inflammation and metabolic homeostasis. Bioengineered. 2021;12: 7495-7507.

40. Tang X, Qi C, Zhou H, Liu Y. Critical roles of PTPN family members regulated by non-coding RNAs in tumorigenesis and immunotherapy. Front Oncol. 2022;12: 972906.

41. Kudo K, Uchida T, Sawada M, Nakamura Y, Yoneda A, Fukami K. Phospholipase C δ1 in macrophages negatively regulates TLR4-induced proinflammatory cytokine production and Fcγ receptor-mediated phagocytosis. Adv Biol Regul. 2016;61: 68-79.

42. Cui N, Hu M, Khalil RA. Biochemical and Biological Attributes of Matrix Metalloproteinases. Prog Mol Biol Transl Sci. 2017;147: 1-73

43. Cai M, Chen N. The Roles of IRF-8 in Regulating IL-9-Mediated Immunologic Mechanisms in the Development of DLBCL: A State-of-the-Art Literature Review. Front Oncol. 2022;12: 817069.

44. Ning W, Acharya A, Sun Z, Ogbuehi AC, Li C, Hua S, et al. Deep Learning Reveals Key Immunosuppression Genes and Distinct Immunotypes in Periodontitis. Front Genet. 2021;12: 648329.

45. Song L, Yao J, He Z, Xu B. Genes related to inflammation and bone loss process in periodontitis suggested by bioinformatics methods. BMC Oral Health. 2015 Sep 4;15: 105.

46. Statello L, Guo CJ, Chen LL, Huarte M. Gene regulation by long non-coding RNAs and its biological functions. Nat Rev Mol Cell Biol. 2021; 22: 96-118.

Della Bella E, Koch J, Baerenfaller K. Translation and emerging functions of non-coding RNAs in inflammation and immunity. Allergy. 2022;77: 2025-2037.

47. Sayad A, Mirzajani S, Gholami L, Razzaghi P, Ghafouri-Fard S, Taheri M. Emerging role of long non-coding RNAs in the pathogenesis of periodontitis. Biomed Pharmacother. 2020;129: 110362.

48. Xu J, Yin Y, Lin Y, Tian M, Liu T, Li X, et al. Long non-coding RNAs: Emerging roles in periodontitis. J Periodontal Res. 2021; 56: 848-862.

49. Chang W, Wang M, Zhang Y, Yu F, Hu B, Goljanek-Whysall K, et al. Roles of long noncoding RNAs and small extracellular vesicle-long noncoding RNAs in type 2 diabetes. Traffic. 2022; 23: 526-537.

50. Chen Y, He Y, Zhou H. The potential role of lncRNAs in diabetes and diabetic microvascular complications. Endocr J. 2020 Jul 28;67(7):659-668.

51. Tanwar VS, Reddy MA, Natarajan R. Emerging Role of Long Non-Coding RNAs in Diabetic Vascular Complications. Front Endocrinol (Lausanne). 2021;12: 665811.

52. Bergstrand S, O'Brien EM, Coucoravas C, Hrossova D, Peirasmaki D, Schmidli S, et al. Small Cajal body-associated RNA 2 (scaRNA2) regulates DNA repair pathway choice by inhibiting DNA-PK. Nat Commun. 2022;13: 1015.

53. Ching T, Huang S, Garmire LX. Power analysis and sample size estimation for RNA-Seq differential expression. RNA. 2014;20:1684-1696.

54. Chapple ILC, Mealey BL, Van Dyke TE, Bartold PM, Dommisch H, Eickholz P, et al. Periodontal health and gingival diseases and conditions on an intact and a reduced periodontium: Consensus report of workgroup 1 of the 2017 World Workshop on the Classification of Periodontal and Peri-Implant Diseases and Conditions. J Clin Periodontol. 2018;45 Suppl 20: S68-S77.

55. Papapanou PN, Sanz M, Buduneli N, Dietrich T, Feres M, Fine DH, et al. Periodontitis: Consensus report of workgroup 2 of the 2017 World Workshop on the Classification of Periodontal and Peri-Implant Diseases and Conditions. J Clin Periodontol. 2018;45 Suppl 20: S162-S170.

56. Jepsen S, Caton JG, Albandar JM, Bissada NF, Bouchard P, Cortellini P, et al. Periodontal manifestations of systemic diseases and developmental and acquired conditions: Consensus report of workgroup 3 of the 2017 World Workshop on the Classification of Periodontal and Peri-Implant Diseases and Conditions. J Periodontol. 2018; 89 Suppl 1: S237-S248.

57. FASQC, http://www.bioinformatics.babraham.ac.uk/projects/fastqc/.

58. Bolger AM, Lohse M, Usadel B. Trimmomatic: a flexible trimmer for Illumina sequence data. Bioinformatics. 2014; 30: 2114-2120.

59. Dobin A, Davis CA, Schlesinger F, Drenkow J, Zaleski C, Jha S, et al. STAR: ultrafast universal RNA-seq aligner. Bioinformatics. 2013; 29:15-21.

60. Li B, Dewey CN. RSEM: accurate transcript quantification from RNA-Seq data with or without a reference genome. BMC Bioinformatics. 2011;12: 323.

61. Robinson MD, McCarthy DJ, Smyth GK. edgeR: a Bioconductor package for differential expression analysis of digital gene expression data. Bioinformatics. 2010;26:139-40.

Supplemental/ Supporting information

Table S1. Demographic characteristics, glycemic and periodontal parameters (mean ± SD) of the study population.

Table S2. DEGs in tissues with periodontitis in non-diabetics relative to healthy tissues in non-diabetics exclusively.

Table S3. DEGs in tissues with periodontitis relative to healthy tissues observed in patients with T2DM exclusively.

Table S4. DEGs in tissues with periodontitis in relation to healthy tissues observed in non-diabetic patients and in patients with T2DM concurrently.

Table S5. Ingenuity canonical pathways related to DEGs expressed exclusively in non-diabetic patients and the respective genes that function within each of these signaling pathways.

Table S6. Ingenuity canonical pathways related to the DEGs expressed exclusively in patients with T2DM and the respective genes that function within these signaling pathways.

Fig. S1. The DEGs expressed in the non-diabetic patients (yellow), in patients with T2DM (blue) and the commonly expressed in both the non-diabetic and diabetic patients. HH: non-diabetic patients without periodontitis; HP: non-

diabetic patients with periodontitis; DH: T2DM patients without periodontitis; DP: T2DM patients with periodontitis.

Figure

Click here to access/download;Figure;Fig 1.jpeg

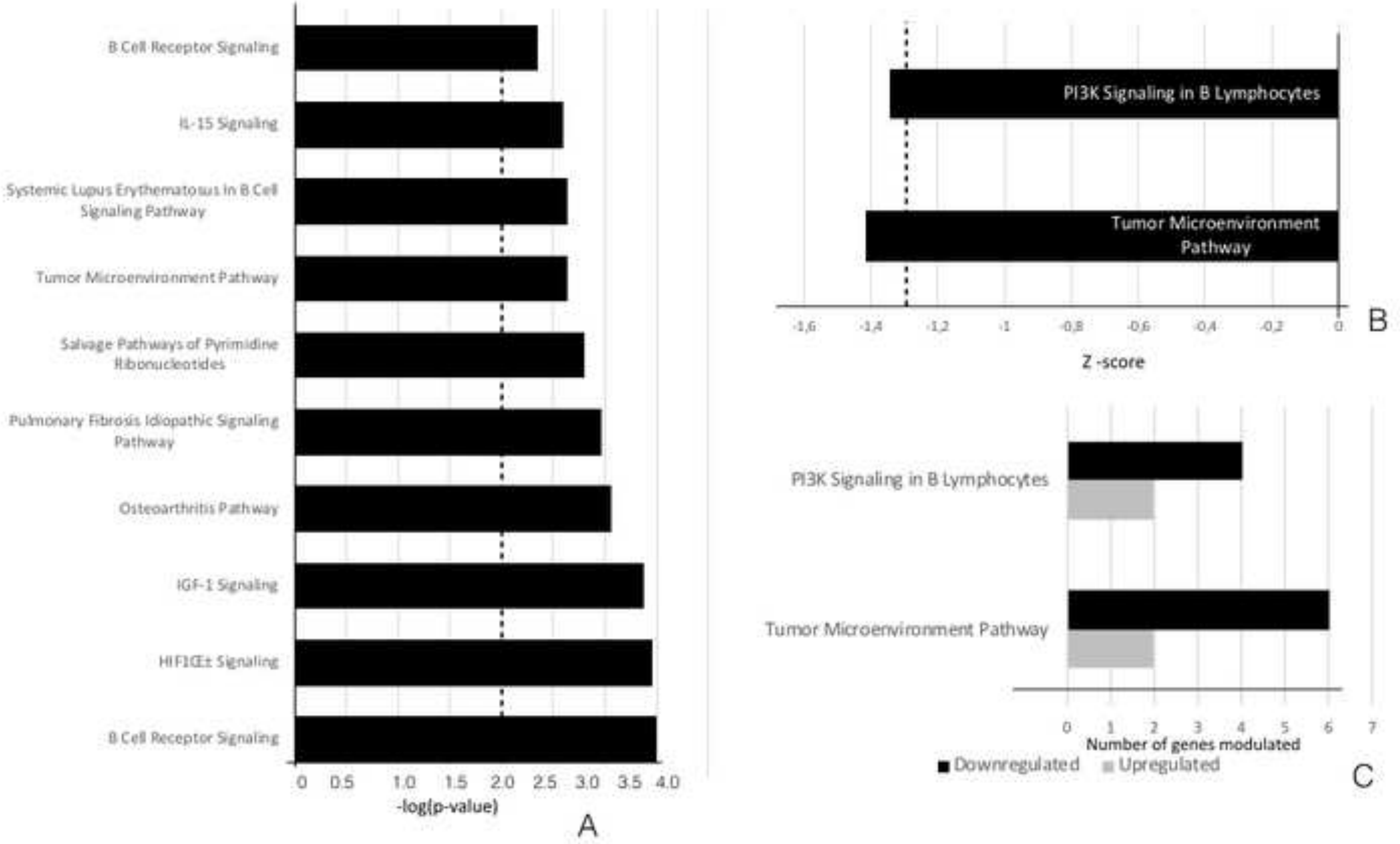

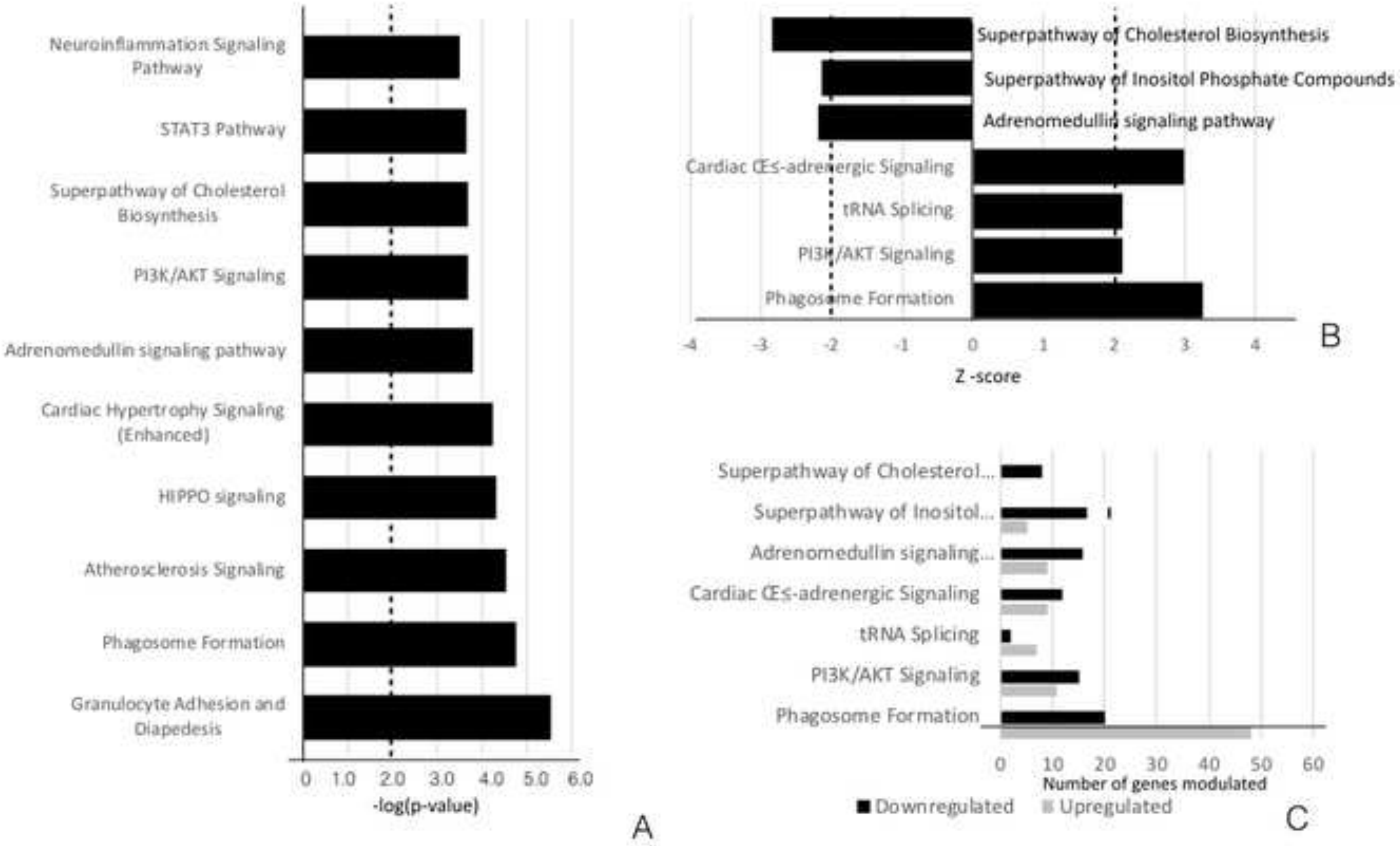
Neuroinflammation Signaling Pathway
STAT3 Pathway
Superpathway of Cholesterol Biosynthesis
PI3K/AKT Signaling
Adrenomedullin signaling pathway
Cardiac Hypertrophy Signaling (Enhanced)
HIPPO signaling
Atherosclerosis Signaling
Phagosome Formation
Granulocyte Adhesion and Diapedesis
0 1.0 2.0 3.0 4.0 5.0 6.0
-log(p-value)
A
Superpathway of Cholesterol Biosynthesis
Superpathway of Inositol Phosphate Compounds
Adrenomedullin signaling pathway
Cardiac Œ≤-adrenergic Signaling
tRNA Splicing
PI3K/AKT Signaling
Phagosome Formation
-4 -3 -2 -1 0 1 2 3 4
Z -score
B
Superpathway of Cholesterol...
Superpathway of Inositol...
Adrenomedullin signaling...
Cardiac Œ≤-adrenergic Signaling
tRNA Splicing
PI3K/AKT Signaling
Phagosome Formation
0 10 20 30 40 50 60
Number of genes modulated
Downregulated
Upregulated
C

Figure

Click here to access/download;Figure;Fig. 3.tiff

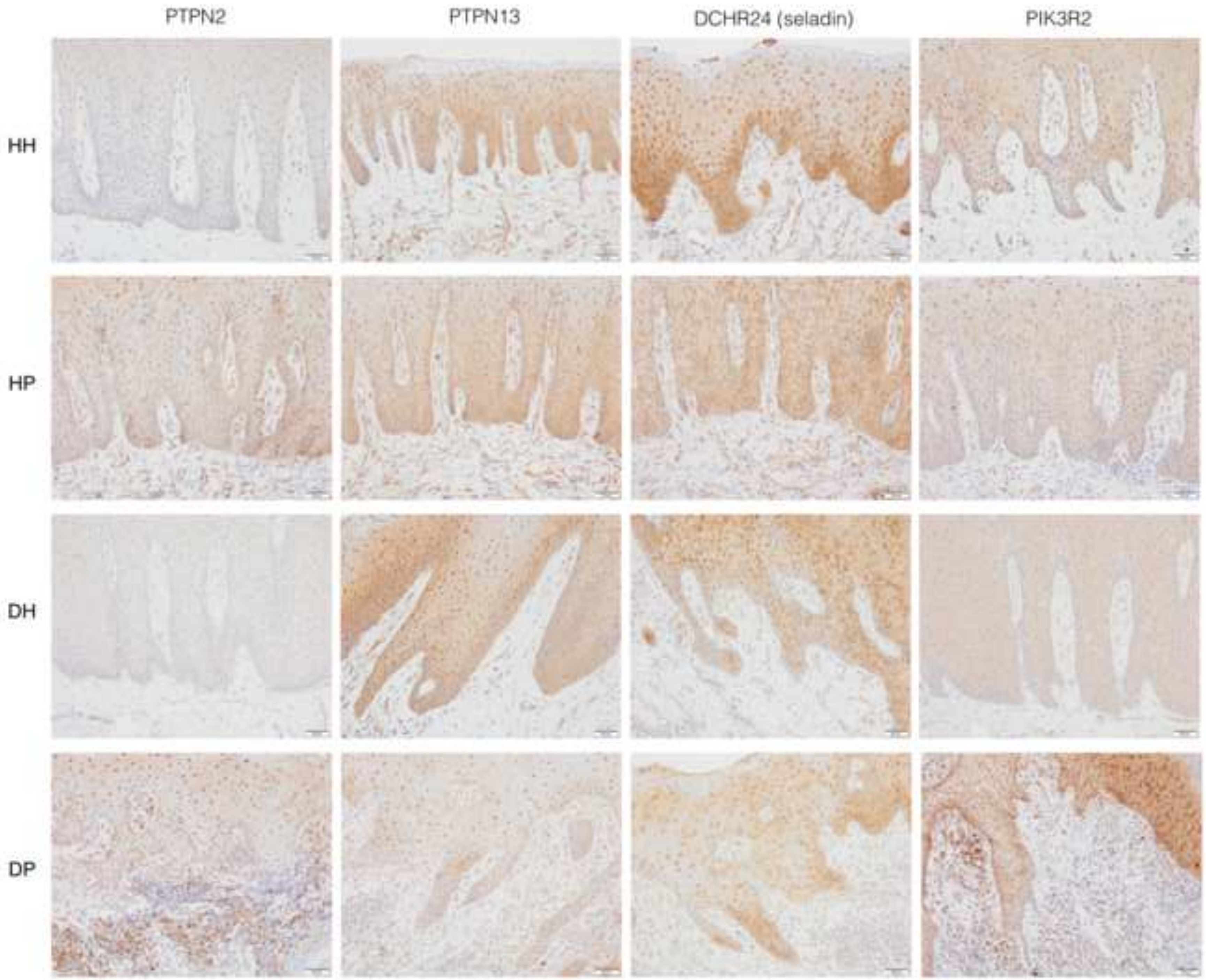

Figure
Click here to access/download;Figure;Fig. 4.tiff

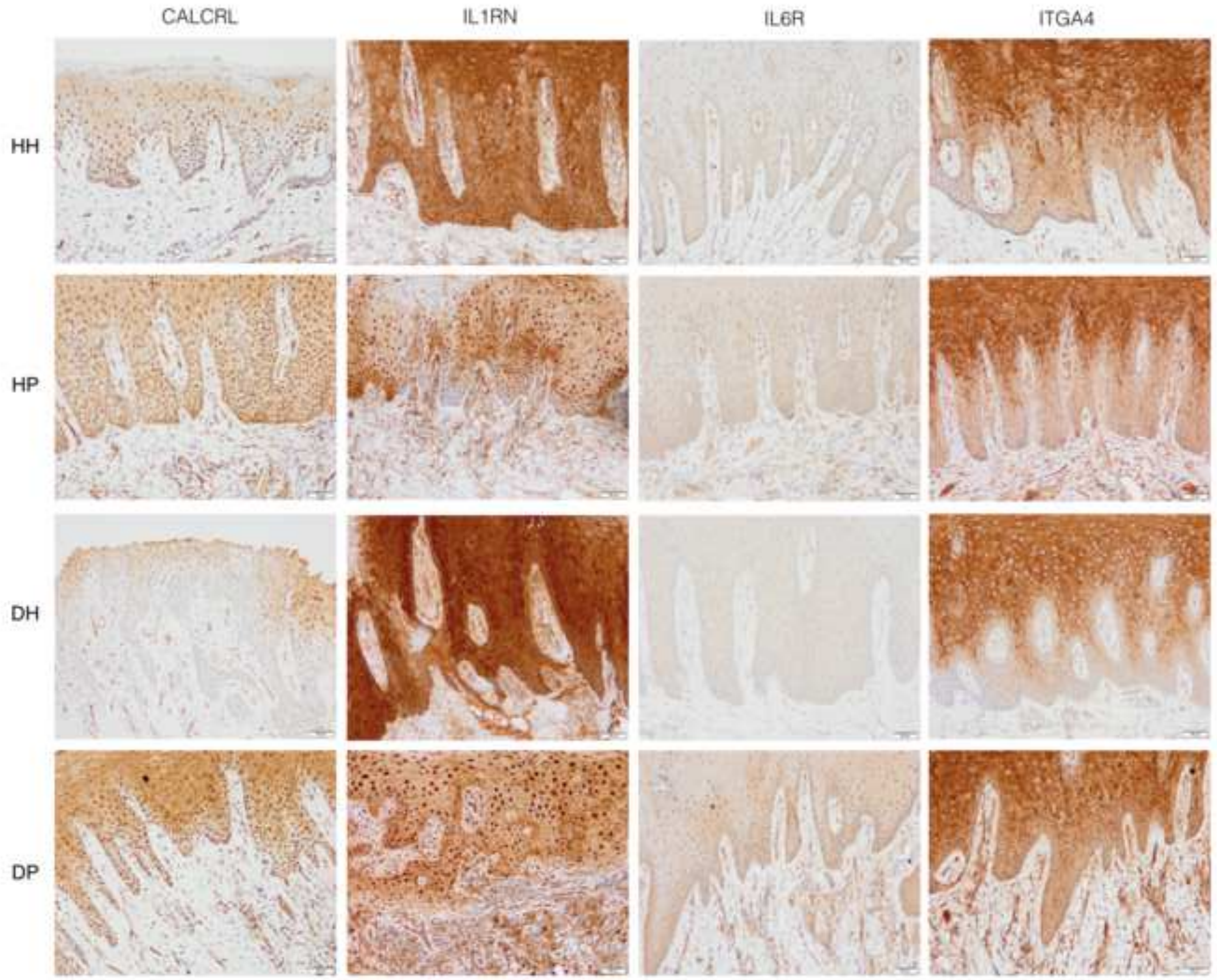

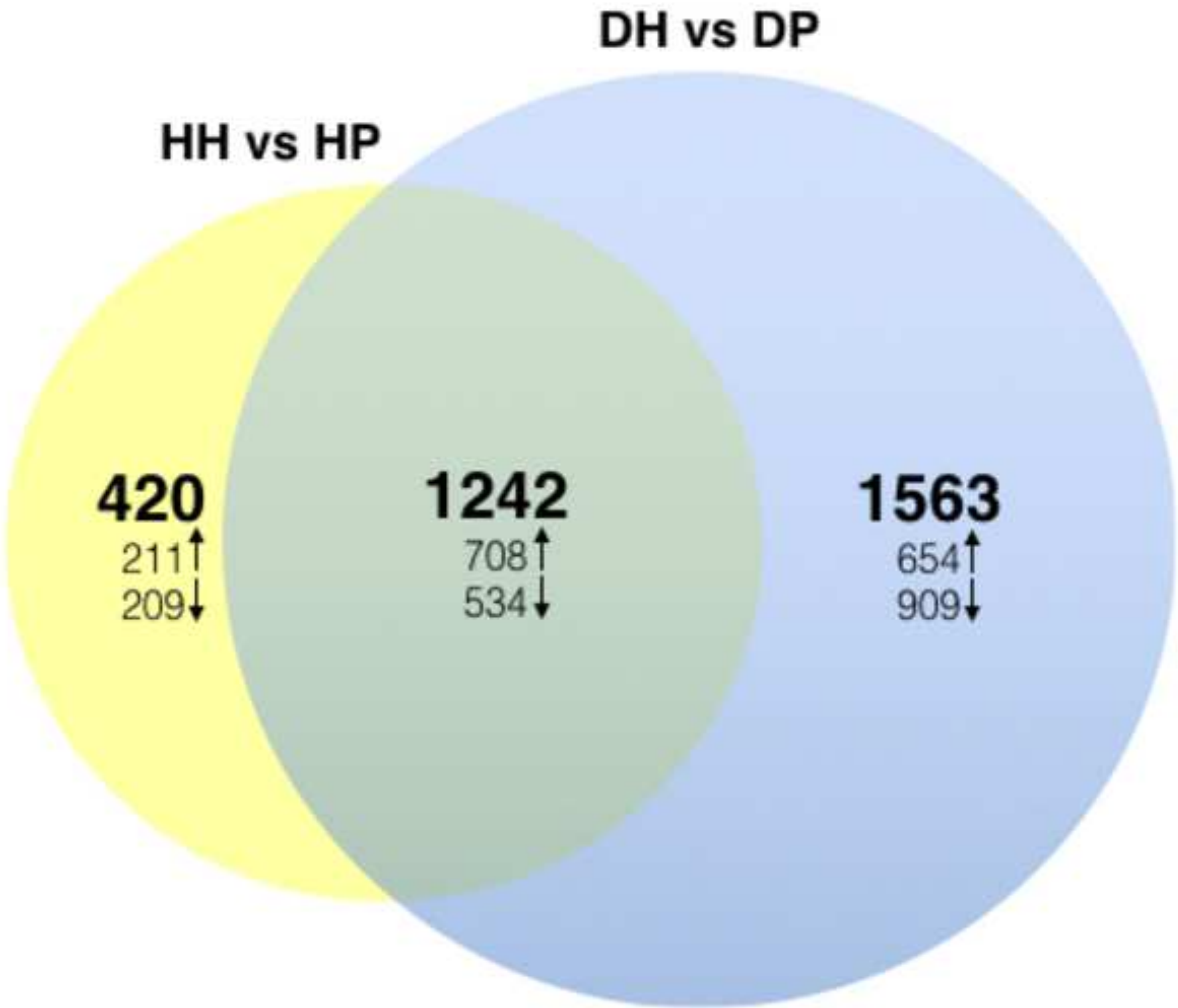
DH vs DP
HH vs HP
420
211↑
209↓
1242
708↑
534↓
1563
654↑
909↓

Figure

Click here to access/download
Supporting Information
Table S1.docx

Click here to access/download
Supporting Information
Table S2.xlsx

Click here to access/download
Supporting Information
Table S3.xlsx

Click here to access/download
Supporting Information
Table S4.xlsx

Click here to access/download
Supporting Information
Table S5.docx

Click here to access/download
Supporting Information
Table S6.docx
