# Supplementary material for: Distinctive genes and signaling pathways associated with type 2 diabetes-related periodontitis: Preliminary study
Source: PLoS One. 2024 Jan 19;19(1):e0296925. doi: 10.1371/journal.pone.0296925 (PMC10798476; doi:10.1371/journal.pone.0296925)
Supplement: Attachment — Submitted filename: Review.docx [file pone.0296925.s009.docx]

Dear Editor,

The authors evaluated the whole transcriptome profile and related histopathological analysis of the periodontitis patients with and without T2DM and non-periodontitis patients with and without T2DM from gingival tissue samples.

The determination of the numbers of study groups, collection of samples during which surgical procedures, how the samples for IHC were stored, the reason why 4 samples were selected from each group for IHC, and why proteomics was not conducted have not been explained.

I believe that the results of this study necessitate researchers to write a more specific conclusion. What is expected to be served by the results of this study?

I hope I could provide some support.

Kind Regards,

Dr. Esra Guzeldemir
