# Supplementary material for: Distinctive genes and signaling pathways associated with type 2 diabetes-related periodontitis: Preliminary study
Source: PLoS One. 2024 Jan 19;19(1):e0296925. doi: 10.1371/journal.pone.0296925 (PMC10798476; doi:10.1371/journal.pone.0296925)
Supplement: Attachment — Submitted filename: Review1_Editor.docx [file pone.0296925.s011.docx]

Dear Editor,

I have reviewed the responses on the paper. It would be helpful if track changes were enabled to facilitate easier tracking of the modifications.

I recommend accepting the paper this time.

I trust my feedback proves supportive.

Wishing you a wonderful holiday season and a Merry Christmas.

Kind Regards,

Dr. Esra Guzeldemir
